# Stereochemistry-dependent thermotropic liquid crystalline phases of monosaccharide-based amphiphiles†

**DOI:** 10.1039/d3sm00939d

**Published:** 2023-10-24

**Authors:** Ida Mattsson, Johanna Majoinen, Manu Lahtinen, Thomas Sandberg, Anna Fogde, Tiina Saloranta-Simell, Orlando J. Rojas, Olli Ikkala, Reko Leino

**Affiliations:** a Laboratory of Molecular Science and Engineering, Johan Gadolin Process Chemistry Centre, Åbo Akademi University FI-20500 Finland reko.leino@abo.fi; b Department of Bioproducts and Biosystems, School of Chemical Engineering, Aalto University FI-00076 Aalto Finland; c Department of Chemistry, University of Jyväskylä FI-40014 Finland; d Bioproducts Institute, Department of Chemical and Biological Engineering, Department of Chemistry and Department of Wood Science, University of British Columbia 2360 East Mall Vancouver BC V6T 1Z4 Canada; e Department of Applied Physics, Aalto University Espoo FI-00076 Finland; f VTT Technical Research Centre of Finland Ltd FI-02150 Finland johanna.majoinen@vtt.fi

## Abstract

Conformational rigidity controls the bulk self-assembly and liquid crystallinity from amphiphilic block molecules to copolymers. The effects of block stereochemistry on the self-assembly have, however, been less explored. Here, we have investigated amphiphilic block molecules involving eight open-chain monosaccharide-based polyol units possessing different stereochemistries, derived from d-glucose, d-galactose, l-arabinose, d-mannose and l-rhamnose (allylated monosaccharides *t*-Glc*, *e*-Glc*, *t*-Gal*, *e*-Gal*, *t*-Ara*, *e*-Ara*, *t*-Man*, and *t*-Rha*), end-functionalized with repulsive tetradecyl alkyl chain blocks to form well-defined amphiphiles with block molecule structures. All compounds studied showed low temperature crystalline phases due to polyol crystallization, and smectic (lamellar) and isotropic phases upon heating in bulk. Hexagonal cylindrical phase was additionally observed for the composition involving *t*-Man*. Cubic phases were observed for *e*-Glc*, *e*-Gal*, *e*-Ara*, and *t*-Rha* derived compounds. Therein, the rich array of WAXS-reflections suggested that the crystalline polyol domains are not ultra-confined in spheres as in classic cubic phases but instead show network-like phase continuity, which is rare in bulk liquid crystals. Importantly, the transition temperatures of the self-assemblies were observed to depend strongly on the polyol stereochemistry. The findings underpin that the stereochemistry in carbohydrate-based assemblies involves complexity, which is an important parameter to be considered in material design when developing self-assemblies for different functions.

## Abstract 2

Stereochemistry of glyco-based high-*χ* block molecules should not be neglected in their assemblies in materials design, especially at the interfaces between the nanophase segregated domains, to master the structural complexity.

## Graphical abstract

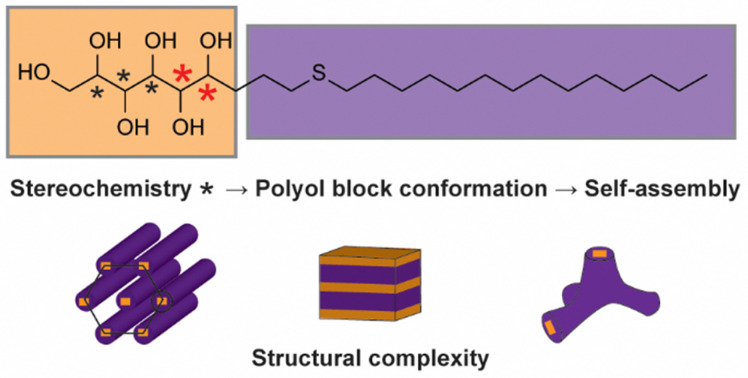

## Introduction

**Scheme 1:**
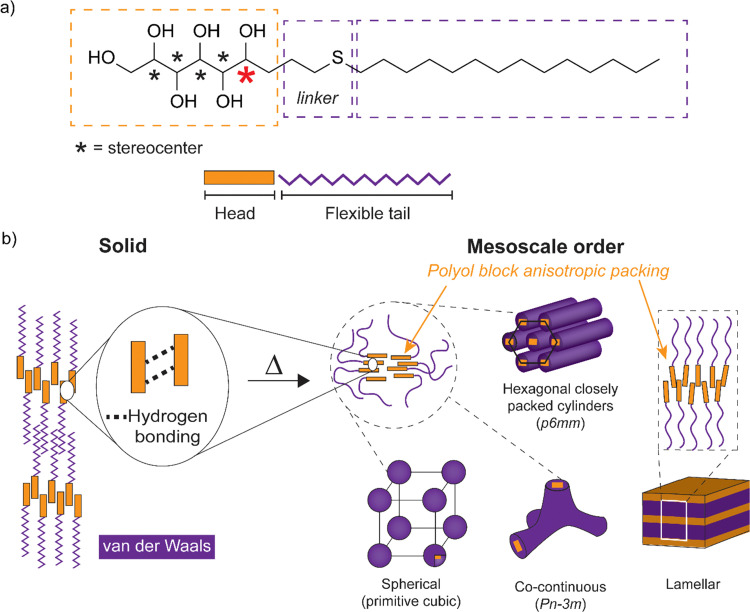
(a) Schematics for the amphiphilic block molecule with monosaccharide-based open-chain hydrogen bonding polyol block connected *via* a linker to a flexible tetradecyl tail block. The five polyol stereocenters are indicated with an asterisk. The stereocenter nearest to the interface separating the head and the tail is marked in red color. (b) Simplified illustration of the expected hydrogen bonding interactions and the nanosegregation between the polyol sections and the alkyl tails. Schemes for the mesoscale order comprising cubic spherical/bicontinuous, hexagonal closely packed cylinders and lamellar structures (with dynamic hydrogen bonding) are obtained upon heating. Example space groups for lattice systems are given in brackets.

Control of self-assembled soft matter structures from the mesoscale down to a few nanometer scale is relevant in science and for various fields of technology, from lithography to functional materials.^1–4^ Therein, the coil-coil diblock copolymers (BCPs) have been the classic enablers for lamellar, bicontinuous gyroid, cylindrical, and spherical structures.^5,6^ Such structures depend on the polymer block volume fractions, the number of monomeric units (*N, i.e.*, the molecular weight), and the repulsion between the blocks, *i.e.*, the Flory–Huggins parameter (*χ*). The self-assembled nanophase segregation is obtained only if *χN* is sufficiently large. This limits classic coil-block self-assemblies to relatively large molecular weights (large *N*) which, in turn, results in relatively large self-assembled periodicities. Several approaches have been pursued aiming at smaller self-assembly length scales towards higher repulsion (*χ*) between the blocks, denoted as high-*χ* block copolymers.^4,7^ Rigid rods in the form of protein secondary helical structures, π-conjugated polyacetylene or aromatic group containing molecules have been used to combine with coiled blocks, denoted as rod-coil BCPs.^8^ They allow rod block anisotropic packings leading to liquid crystalline nanodomains.^9^ Carbohydrates, capable of multiple hydrogen bonding, and consequently polyols, also express rod-like nature with anisotropic packing and liquid crystalline properties for their amphiphilic block molecule derivatives (Scheme 1). Therein, hydrogen bonding either stabilizes the rigid-rod structure and the forming liquid crystal or it enhances the phase segregation of the hydrophilic and hydrophobic blocks inducing the liquid crystal formation.^10^ Chemical tailoring of the nanodomain interfaces promotes the tendency for self-assemblies, for example using ionic groups.^11,12^ Carbohydrate-based amphiphilic block molecules^13–16^ allow the increase of *χ* and further decrease in self-assembled bulk domain sizes *via* transformation from BCP building block dimensions to small molecules where unique properties such as liquid crystallinity emerge for utilization.^17^ Upon sufficiently reducing the molecular weight in the self-assembling bulk high-*χ* block copolymers, the compositions can also be considered in the perspectives of solvent-free amphiphiles and thermotropic liquid crystals.

Carbohydrates form a particularly attractive platform for the design of self-assembling structures due to their natural abundance, biodegradability, interactions, and structural diversity. At the same time, they provide significant molecular complexity due to their rich stereochemistry. Consequently, it becomes possible to study the influence of configuration on the self-assembly and liquid crystalline properties.^18,19^ A number of monosaccharide-derived amphiphiles have been shown to possess liquid crystalline properties,^19–23^ typically consisting of an aliphatic flexible alkyl chain coupled to one of the hydroxyl groups in the cyclic or open-chain monosaccharide (polyol) unit through a molecular linker. Carbohydrate-derived liquid crystals exhibit both hexagonal and cubic structures.^20,24–37^ However, novel phases with cubic networks have been shown *via* specific bolapolyphile molecular design, which utilizes π-conjugated glycerol end capped benzene rings as rod blocks.^38,39^ Hydrogen bonding of the carbohydrate head block plays a crucial role in demonstrating cubic liquid crystalline phases.^38,40,41^ Thermotropic cubic phases have remained poorly understood, since their structures cannot be easily predicted from the shape and configuration of the molecules involved in their formation.^40,42,43^ Concerning carbohydrate-derived amphiphiles, cubic mesophases can be obtained in polar solvents,^44–48^ while appearing to be much less common under purely thermotropic conditions.^41^ The monosaccharide ring structure is known to stabilize the liquid crystal state with primarily equatorially oriented hydroxyl groups within the ring.^36^ However, conformational effects of the open-chain carbohydrate derivatives on the formation of the liquid crystal state have remained relatively unexplored. In this study, attention is paid in particular to the effects of the polyol stereochemistry near the phase separating interface of carbohydrate based amphiphilic block molecules.

**Fig. 1:**
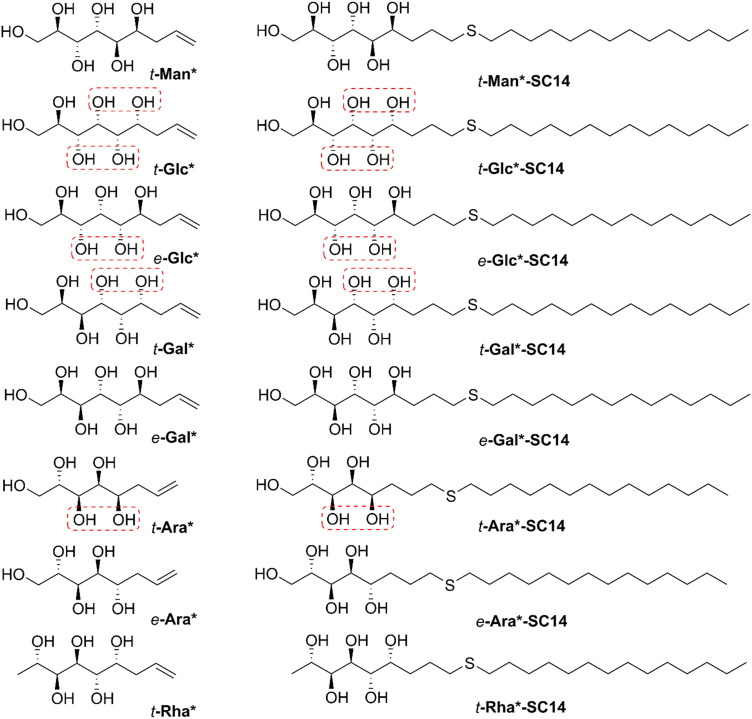
Chemical structures of the allylated monosaccharides and their corresponding amphiphilic block molecules studied in this work. The red squares indicate 1,3-*syn* relationship of the hydroxyl groups within the polyol head block (*t* corresponds to the *threo* and *e* to the *erythro* diastereomeric forms, resulting from the new stereocenter formed in the allylation reaction).

**Scheme 2:**
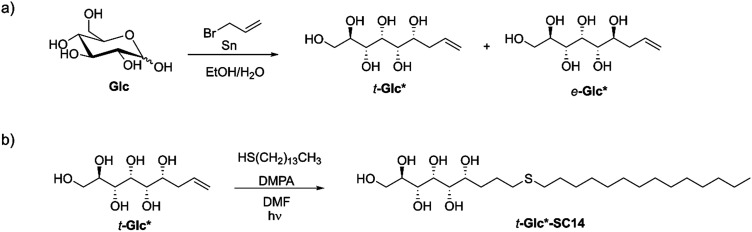
(a) An example of synthesis of allylated monosaccharides *t*-Glc* and *e*-Glc* by tin-mediated allylation of d-glucose (Glc) as the parent monosaccharide. (b) Synthesis of the liquid crystalline compound *t*-Glc*-SC14 from *t*-Glc* by thiol–ene click-reaction.

Herein, to explore the effects of stereochemistry on the self-assembling properties (Scheme 1), we have prepared a series of open-chain monosaccharide-based amphiphilic block molecules with different stereochemistries by tin-mediated allylation of monosaccharides, followed by UV-induced thiol–ene reaction (for structures see Fig. 1; for reactions see Scheme 2). The first part of the work consists of characterization of the parent allylated polyols in detail. The second part comprises amphiphilic block molecule synthesis and self-assembly studies. The molecular compositions studied are based on d-glucose, d-galactose, l-arabinose, d-mannose, and l-rhamnose (allylated monosaccharides *t*-Glc*, *e*-Glc*, *t*-Gal*, *e*-Gal*, *t*-Ara*, *e*-Ara*, *t*-Man*, and *t*-Rha*), end-functionalized with alkyl chain blocks. The compounds prepared were thoroughly characterized by nuclear magnetic resonance (NMR) spectroscopy, differential scanning calorimetry (DSC), thermogravimetric analysis (TGA), polarized optical microscopy (POM), and small/wide-angle X-ray scattering (SWAXS).

## Experimental section

### Materials

All reagents and solvents were received from commercial suppliers and used without further purification.

### Molecular characterization

NMR spectra were recorded at 298 K on a Bruker Avance-III HD 500 MHz spectrometer equipped with a Bruker SmartProbe™. Deuterated DMSO with 0.03% tetramethylsilane (TMS) as internal standard or D_2_O were used as solvents. HRMS were recorded with a Bruker Daltonics micro-QToF instrument in positive mode using ESI-ionization.

### General method for allylation of unprotected monosaccharides

Allylation of the unprotected monosaccharides was conducted in aqueous media according to previously published protocols.^49–52^ The analytical data is given in the ESI† and was for the previously reported compounds *t*-Man*, *t*-Glc*, *t*-Gal* in accordance with literature.^52,53^ In a typical procedure, the corresponding monosaccharide (27.8 mmol, 1 eq.), tin powder (55.6 mmol, 2 eq.) and allyl bromide (83.4 mmol, 3 eq.) were dispersed in EtOH : H_2_O 11 : 1. The mixture was heated to 60 °C and stirred rigorously overnight. The mixture was allowed to cool to room temperature and subsequently neutralized with 5 M NaOH (aq). Next, the solution was filtered through Celite and the solvent evaporated to dryness. The diastereomers formed by allylation of d-glucose (*t*-Glc* and *e*-Glc*), d-galactose (*t*-Gal* and *e*-Gal*) and l-arabinose (*t*-Ara* and *e*-Ara*) were separated by acetylation followed by column chromatography and deacetylation, while the major diastereomers formed by allylation of d-mannose (*t*-Man*) and l-rhamnose (*t*-Rha*) were separated by direct recrystallization. Further details on the allylation procedure are provided in the ESI.†

### General method for UV-induced thiol–ene reactions

For further transformation of the allylated monosaccharides into diblock-type compounds, the terminal double bond of the polyol chain can be reacted *e.g.*, in a UV-induced thiol–ene click-reaction.^54^ Here, the corresponding allylated monosaccharide (1 eq.), thiol (2 eq.) and 2,2-dimethoxy-2-phenylacetophenone (0.1 eq.) were dissolved in DMF. The solution was irradiated with 356 nm 125 W UV-light for 1 h. The solvent was removed under reduced pressure and the residual initiator and thiol were removed by washing multiple times with hexane. The solids were separated as pure compounds (72–94% yield) by centrifugation/decantation and drying *in vacuo*. Further details on the thiol–ene click reactions are provided in the ESI.† All UV-induced reactions were conducted in a 75 ml immersion well reactor equipped with a 125 W lamp emitting UV light at 365 nm wavelength. The reactor system was purchased from Photochemical Reactors LTD.

### Thermogravimetric analysis

Thermogravimetric data were acquired on a PerkinElmer STA 6000 simultaneous thermogravimetric-calorimetric TG/DSC analyser. Each sample was placed in an open platinum crucible and heated under air atmosphere (flow rate of 40 ml min^−1^) using a heating rate of 10 °C min^−1^ in the temperature range between 20 and 600 °C. Melting points of indium and zinc metal standards were used to calibrate the device temperature and a standard weight of 50 mg was used to calibrate the weight. Sample weights used in the measurements were in the range of 4–5 mg. Fig. S13 (ESI†).

### Differential scanning calorimetry

Thermal transitions of *t*-Man*, *t*-Glc*, *e*-Glc*, *t*-Gal*, *e*-Gal*, *t*-Ara*, *e*-Ara*, *t*-Rha*, *t*-Man*-SC6 and *t*-Man*-SC10 were obtained using a TA Instruments Discovery DSC250 differential scanning calorimeter. All measurements were conducted under nitrogen atmosphere (50 ml min^−1^ flow rate) in standard TZero aluminium cups. TZero calibration was performed with sapphire discs and cell constant calibration against an indium standard. The samples were heated (10 °C min^−1^) once from 20 °C to a sample-dependent maximum temperature approx. 10–20 °C higher than the melting temperature. For the samples *t*-Man*-SC6 and *t*-Man*-SC10, a heat-cool ramp was repeated twice with a rate of 10 °C min^−1^. The thermal transitions were determined as an extrapolated onset. Sample weights were approx. 1–3 mg.

Thermal transitions of the compounds *t*-Man*-SC14, *t*-Glc*-SC14, *e*-Glc*-SC14, *t*-Gal*-SC14, *e*-Gal*-SC14, *t*-Ara*-SC14, *e*-Ara*-SC14 and *t*-Rha*-SC14 were measured using a power compensation type PerkinElmer 8500 series differential scanning calorimeter. All heating/cooling scans were carried out under nitrogen atmosphere (flow rate 50 ml min^−1^) using 50 μl aluminium pans sealed by 30 μl aluminium pan with pinholes (resulting 20 μl free volume for a sample). Temperature calibration was performed using two standard materials (*n*-decane and indium) and the energy calibration by using an indium standard. Each sample was typically heated and cooled twice from 20 to about 10 °C above the pre-determined isotropization temperature (based on TG/DSC runs), with a heating and cooling rate of 10 °C min^−1^. The thermal transitions were determined as extrapolated onsets. Sample weights used on the measurements ranged from 1–2 mg.

### Polarized optical microscopy (POM)

POM images were obtained using an OLYMPUS BX51 stereo microscope (typical magnification: 100×) equipped with a Linkam LTS 420E temperature-controlled microscope stage, LN95-LTS liquid N_2_ cooling unit, OLYMPUS DP-26 high-resolution color CCD-camera, and an OLYMPUS Stream image analysis program. Temperature calibration of the hot stage was carried out using the melting of an indium standard (156.6 °C). For analysis, a small amount of the sample (<1 mg) was formed as a thin layer on a standard microscope slide covered with a thin cover glass slide. Different heating/cooling rates were used (typically 1–10 °C min^−1^), depending on the sample, and used to observe the thermal events indicated by the DSC analyses. Alternatively, imaging was conducted following similar sample preparation and using a Leica DM450 polarized optical microscope (mag 20×) equipped with a temperature-controlled stage and a mounted Canon EOS 60D digital camera.

### Variable temperature small/wide angle X-ray scattering

**Fig. 2:**
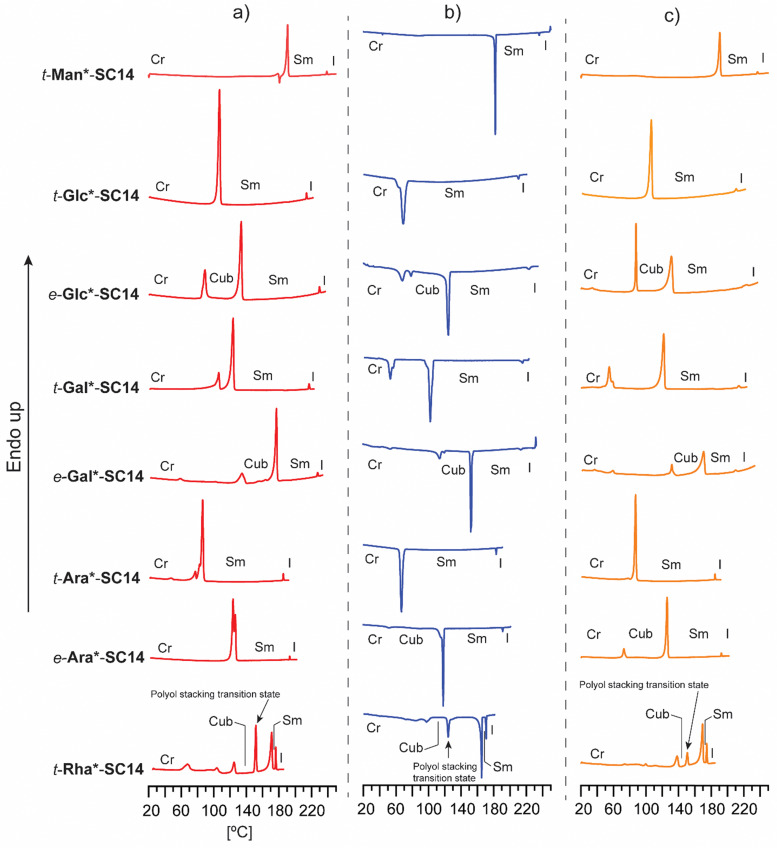
Full DSC analysis with (a) heating, (b) cooling (c) and second heating traces for *t*-Man*-SC14, *t*-Glc*-SC14 and *e*-Glc*-SC14, *t*-Gal*-SC14 and *e*-Gal*-SC14, *t*-Ara*-SC14 and *e*-Ara*-SC14, and *t*-Rha*-SC14. The identified phase transitions (Cr = crystalline, Cub = cubic, Sm = smectic and I = isotropic state) combining POM results are marked in the data.

X-ray analysis was performed using both small and wide angles (SWAXS) to obtain structural information of the liquid crystal phases (mesoscale structures 1–200 nm) and of the crystal structures (atom distances below 1 nm) of the individual blocks within the amphiphiles. The scattering angle (*θ*) defines the structure domain length as *d* = 2π/*q*, where *q* = 4π sin(*θ*)/*λ*. Therefore, scattering data from wider angle translate into small domain lengths compared to the data from smaller angles. The SWAXS data was obtained with a Xeuss 3.0 instrument using Cu Kα radiation (*λ* = 1. 5418 Å) as the X-ray source and a setup equipped with a heater. Sample to detector distance was set to 0.15 m. The compounds studied were thoroughly lyophilized prior to the SWAXS analysis to exclude the presence of water in the samples. As thermal analysis also showed no indication to the presence of water, we exclude the discussion of hydrate crystals^55^ from this work. Temperatures for data collection were selected according to the thermal transitions (DSC measurements, Table 2 and Fig. 2). Powdered samples were placed in measuring wells between thin Kapton foils. The powders were heated over the melting temperature (heating to ∼200 °C and cooling to 25 °C) to fill the well with optimal amount of sample (approx. 20 mg) before sealing. The magnitude of the scattering vector is given by *q* = (4π/*λ*) sin *θ*, where 2*θ* is the scattering angle. The *q* range was calibrated using an LaB_6_ standard. The data was collected after reaching the desired temperature.

## Results and discussion

### Synthesis and characterization of allylated monosaccharides

Tin-mediated allylation of unprotected monosaccharides in aqueous media, first disclosed by Whitesides and coworkers,^49,50^ provides convenient access to stereochemically pure alkene-terminated polyols in a single step. Due to formation of a new stereocenter in this carbon–carbon bond forming reaction, the allylated products are received as mixtures of *threo* (*t*) and *erythro* (*e*) diastereomers (Scheme 2a) in variable ratios, depending on the monosaccharide used, separable by direct crystallization^46,51,52^ or derivatization followed by column chromatography^43^ and subsequent deprotection.^52^ Here, the diastereomerically pure allylated monosaccharides *t*-Man*, *t*-Glc*, *e*-Glc*, *t*-Gal*, *e*-Gal*, *t*-Ara*, *e*-Ara* and *t*-Rha*, prepared by tin-mediated allylation of the corresponding parent monosaccharides d-mannose (Man), d-glucose (Glc), d-galactose (Gal), l-arabinose (Ara) or l-rhamnose (Rha), were investigated as possible starting material for novel diblock liquid crystalline compounds and materials.

For simplicity, the carbon chain of the polyol structures is typically drawn in planar zigzag conformation (Fig. 1 and Scheme 2), although the actual preferred conformations in aqueous solution depend on the relative stereochemistry of the hydroxyl groups. To exemplify, we have previously shown by NMR spectroscopic analysis and computational methods that the *threo*-diastereomer of allylated d-mannose (*t*-Man*), the corresponding propargylated analogue and the fully saturated congener adopt almost perfectly planar zigzag conformations both in solid state and in solution, whereas the corresponding d-glucose and d-galactose derivatives, *t*-Glc* and *t*-Gal* are non-planar.^51,52^ In this study, the series was extended to also include *e*-Glc*, *e*-Gal*, *t*-Ara*, *e*-Ara* and *t*-Rha* (for structures, see Fig. 1).

Typically, the driving forces behind the minimum energy conformations of polyols are the maximization of *gauche* effects between their vicinal hydroxyl groups, minimization of 1,3-*syn* interactions between the hydroxyl groups, and minimization of steric interactions through formation of a zigzag conformation. The latter, however, appears to be overruled by the former two, and in order to minimize the 1,3-*syn* interactions the polyol adopts a sickle-like conformation rather than a linear one.^56^ Conformations of acyclic carbohydrates can be elucidated by NMR spectroscopy, as shown earlier in the extensive studies by Lewis and coworkes.^57–60^ More recently, a more universal ^3^*J*_H,H_-coupling constant based conformational model has been developed by Murata *et al.*, concluding that for linear polyols in planar zigzag conformation, the ^3^*J*_H,H_ coupling constants should be either small or large.^61^ Small (<3 Hz) ^3^*J*_H,H_ coupling constants correspond to dihedral angles of around 60°, or *gauche* orientation, while large (7–10 Hz) coupling constants are consistent with *anti*-orientation, or a dihedral angle of 180°, of the vicinal protons. Consequently, intermediate (3–7 Hz) ^3^*J*_H,H_ coupling constants indicate the predominance of non-planar conformations. For polyol backbones in zigzag conformation, two adjacent protons in *threo* relationship generally occur in *gauche* orientation, resulting in a small coupling constant, while protons in *erythro* relationship are expected to favor an *anti*-orientation, resulting in a larger coupling constant.

For accurate interpretation of the NMR spectroscopic data in this study, ChemAdder/Spinadder software (ChemAdder/Spinadder Spin Discoveries Ltd.) with spin simulation/iteration techniques was utilized. The relevant coupling constants for all allylated monosaccharides studied are collected in Table S1 (ESI†). Compounds *e*-Gal*, *e*-Ara* and *t*-Rha* (Fig. 1) do not contain any hydroxyl groups in 1,3-*syn* relationship and can thus be assumed to adopt a planar zig-zag conformation in aqueous solution. This was further confirmed by the ^3^*J*_H,H_ coupling constants, which follow the pattern of being either small or large. In contrast, *e*-Glc* and *t*-Ara* (Fig. 1) possess one 1,3-*syn* interaction between the OH-groups and should therefore adopt a bent or sickle-like minimum energy conformation rather than planar. Here, the ^3^*J*_H,H_ coupling constants are consistent with what can be expected from compounds containing hydroxyl groups in 1,3-*syn* relationship, as all of the compounds with such configuration studied in the present work are characterized by at least one medium sized ^3^*J*_H,H_ coupling constant. It should be noted, however, that the coupling constants cannot be directly translated or applied to the corresponding conformations in solid state, with crystallization being a kinetic phenomenon and the conformation in the crystal state being determined also by molecular packing forces and interactions not necessarily related to minimum conformations in solution.

To further support the observations based on the conformational NMR spectroscopic studies, and to gain additional information on the relevant structural parameters, each allylated monosaccharide structure was optimized computationally. Geometry optimizations of the compounds *e*-Glc*, *e*-Gal*, *t*-Ara*, *e*-Ara* and *t*-Rha in aqueous solution were conducted by a multi-level deterministic structural optimization (the corresponding analysis of the compounds *t*-Man*, *t*-Glc* and *t*-Gal* has been reported previously).^45^ The optimized COSMO-solvated geometries of all allylated monosaccharides studied in this work are illustrated in Table S1 (ESI†). The calculations performed broadly agree with the experimentally observed planarity differences between the different compounds. For *e*-Gal* and *e*-Ara*, the planar conformations dominate and the angles between the vicinal protons in the optimized structures are well in line with the experimentally obtained coupling constants. The angles between the vicinal protons of the polyol part (C1–C6) of compound *t*-Rha* are, likewise, consistent with the experimentally obtained coupling constants. The C5–C6 bond of compound *t*-Rha* is, however, twisted, leading to the allyl moiety of the compound sticking out from the plane. This unexpected twist could possibly be explained by the ideal 1.81 Å distance of an intramolecular hydrogen bond between the OH-4 and OH-6. Turning the C5–C6 bond in compound *t*-Rha* 180°, to obtain a perfectly planar conformation, would result in a 6.6 kJ/mol higher relative energy. Compounds *e*-Glc* and *t*-Ara* prefer non-planar conformations in aqueous solution, which can also be observed in the vicinal coupling constant patterns as determined by NMR spectroscopy. It should, however, be noted, that the implicit solvation reduces the non-planarity or bend of all structures, and intramolecular hydrogen bonding also affects the planarity of the molecular backbone. In addition, the structures likely exist in multiple Boltzmann distributed conformations under conditions studied in the NMR-experiments. Furthermore, it should be emphasized that the computational studies in this work were conducted in solution and cannot be directly translated to the solid state.

### Thermal analysis

Detailed investigations on the thermal behavior of the allylated monosaccharides *t*-Man*, *t*-Glc* and *t*-Gal* were conducted already in our earlier work.^52^ In the present work, the series is extended to also include the allylated monosaccharides *e*-Glc*, *e*-Gal*, *t*-Ara*, *e*-Ara* and *t*-Rha* to gain further understanding of the relationships between the stereochemical configuration of the polyol chain and the melting point. For consistency, melting points of all the allylated monosaccharides, including those previously reported, were measured by DSC. The main thermal events are summarized in Table S2 (ESI†).

### Liquid crystalline amphiphiles derived from allylated monosaccharides

In order for an amphiphilic compound to exhibit liquid crystalline properties, the hydrophilic/hydrophobic ratio should be well-balanced. Mesomorphic behavior commences at a critical alkyl tail chain length and the clearing point (transition from liquid crystal state to the isotropic state) reaches its maximum temperature when the balance between the polar and non-polar parts of the molecule is optimal.^62^ The clearing temperature in a homologous series of amphiphilic liquid crystals depends on the length of the hydrophobic chain, whereas branching and number of the aliphatic chains influence more the mesomorphic behavior, *i.e.*, which mesophase is formed.^63–65^

Here, the polyol-derived amphiphiles were prepared by coupling the alkene-terminated polyols with aliphatic thiols using the UV-induced thiol–ene click-reaction. The optimal length for hydrophobic carbon chain to be coupled with the allylated monosaccharides was determined by coupling *t*-Man* with three thiols with increasing chain length: 1-hexanethiol (C6), 1-decanethiol (C10) and 1-tetradecanethiol (C14). All three coupling products (denoted as *t*-Man*-SC6, *t*-Man*-SC10 and *t*-Man*-SC14) were analyzed by DSC and POM to investigate the thermal events and potential mesomorphic behavior (Table S3, ESI†). The product obtained from the coupling reaction with 1-hexanethiol, *t*-Man*-SC6, displays liquid crystalline properties, but in a narrow span of only about 7 °C (the crystalline phase melts at 190.9 °C and the isotropization occurs at 198.3 °C). Increasing the chain length to C10 provides *t*-Man*-SC10 with a crystalline melting and clearing points of 186.3 °C and 224.5 °C, respectively. The largest molecule in the series with C14 chain, *t*-Man*-SC14, has the widest liquid crystalline window as the crystalline melting occurs at 183.3 °C and the clearing point is found at 231.5 °C. Based on the trend observed by increasing the chain length even further, the clearing point could shift close to the initiation of thermal degradation temperature, thereby limiting the full utilization of the liquid crystal phase. Traditionally, cyclic monosaccharide based compounds reach the clearing point plateau when the alkyl chain length is between C_14_ and C_16_.^18^ The 1-tetradecanethiol derived compound provides a sufficiently wide mesophase temperature range for all necessary analyses and 1-tetradecanethiol was thus selected as the thiol for coupling with all other allylated monosaccharides. The reaction products precipitated from the mixture during the reaction and were conveniently purified by washing-centrifugation-decantation protocol, providing amphiphilic liquid crystals in 76–94% isolated yields.^54^ The chemical structures and the corresponding (non-systematic) names of the amphiphilic compounds are shown in Fig. 1.

Solubilities of the obtained amphiphiles (*t*-Man*-SC14, *t*-Glc*-SC14, *e*-Glc*-SC14, *t*-Gal*-SC14, *e*-Gal*-SC14, *t*-Ara*-SC14, *e*-Ara*-SC14, *t*-Rha*-SC14) were low in most standard NMR-solvents, and while the spectral quality is sufficient for assignation of the signals, accurate analysis of the coupling constants could not be performed. Nevertheless, we have previously demonstrated that the planar zigzag conformation of the polyol part of *t*-Man* derivatives in the solid state and in solution is not affected when the unsaturated end of the molecule is modified.^54^ Also, the coupling constants, which can be manually extracted from the ^1^H NMR spectroscopic data of the compounds synthesized here, match well both size and pattern-wise with the corresponding coupling constants of the parent polyols (*t*-Man*, *t*-Glc*, *e*-Glc*, *t*-Gal*, *e*-Gal*, *t*-Ara*, *e*-Ara*, *t*-Rha*), indicating that no essential changes have occurred in the conformations of their polyol backbones in solution.

In order to elucidate how the structure of the polar head block affects the thermotropic liquid crystalline behavior, all compounds were analyzed by DSC and POM. The DSC traces from heating, cooling and second heating are collected in Fig. 2. The main thermal events are summarized in Table 1 and in more detail in Table S3 (ESI†). Melting, isotropization and recrystallization peaks were observed for all compounds. POM observations confirmed that all compounds formed smectic (Sm) liquid crystalline phases upon heating/cooling cycles.

**Table 1:** Summary of the thermal properties of the amphiphiles with transition temperatures, associated enthalpy changes [Δ*H*/kJ mol^−1^] and thermal degradation temperatures. Heating (H1), cooling (C1) and second heating (H2). Crystalline (Cr), smectic (Sm) and isotropic (Iso) state. *T*_degr._ is the degradation temperature obtained by TGA

Compound/parent polyol suggested conf.	Thermal events *T*/°C [Δ*H*/kJ mol^−1^]	*T*_degr._ (°C)
*t*-Man*-SC14 linear	H1: Cr-183.21 [113.07] Sm-231.49 [2.79] Iso	264
	C1: Iso-230.06 [−2.92] Sm-177.74 [−109.38] Cr	
	H2: Cr-183.0 [121.26] Sm-229.25 [2.81] Iso	
*t*-Glc*-SC14 non-linear	H1: Cr-104.28 [168.56] Sm-213.23 [3.18] Iso	237
	C1: Iso-211.53 [−3.15] Sm-104.28 [−122.01] Cr	
	H2: Cr-102.83 [131.64] Sm-208.99 [3.03] Iso	
*e*-Glc*-SC14 non-linear	H1: Cr-83.47 [41.53] Cub-125.94 [101.04] Sm-220.33 [3.01] Iso	241
	C1: Iso-217.68 [−2.89] Sm-122.10 [−100.69] Cub-69.46 [−23.36] Cr	
	H2: Cr-84.45 [38.34] Cub-121.93 [72.92] Sm-209.91 [2.76] Iso	
*t*-Gal*-SC14 non-linear	H1: Cr-120.52 [98.11] Sm-216.65 [3.03] Iso	240
	C1: Iso-216.06 [−3.02] Sm-104.10 [−90.0] Cr	
	H2: Cr-118.24 [91.57] Sm-212.04 [1.81] Iso	
*e*-Gal*-SC14 linear	H1: Cr-127.89 [33.83] Cub-173.69 [122.08] Sm-225.70 [2.84] Iso	243
	C1: Iso-216.56 [−2.34] Sm-154.17 [−78.40] Cub-116.73 [−25.04] Cr	
	H2: Cr-129.13 [20.48] Cub-164.68 [80.35] Sm-207.20 [2.17] Iso	
*t*-Ara*-SC14 non-linear	H1: Cr-83.72 [119.37] Sm-184.67 [3.32] Iso	238
	C1: Iso-183.64 [−2.83] Sm-68.96 [−100.95] Cr	
	H2: Cr-85.14 [95.74] Sm-184.0 [2.78] Iso	
*e*-Ara*-SC14 linear	H1: Cr-124.67 [86.13] Sm-192.47 [2.56] Iso	237
	C1: Iso-191.69 [−2.64]-Sm-119.14 [−116.91] Cr	
	H2: Cr-71.55 [20.55] Cub-124.24 [119.15] Sm-191.95 [2.49] Iso	
*t*-Rha*-SC14 linear	H1: Cr-119.83[19.74] Cub-148.49 [40.03] stacking transition-167.07 [57.01] Sm-174.40 [9.87] Iso	240
	C1: Iso-173.84 [−9.50] Sm-168.25 [−51.99] stacking transition-127.31[−25.19] Cub-103.60 [−31.45] Cr	
	H2: Cr-135.09 [16.45] Cub-148.89 [12.49] stacking transition-167.05 [57.77] Sm-173.89 [9.73] Iso	

**Table 2:** Summary of the effects of allylated polyol conformation, and the C5/C6 configuration on the corresponding amphiphilic derivatives liquid crystallinity and domain spacing of the smectic phases (*d*_Lam_)

Allylated polyol (acronym)	Polyol structure^a^	Polyol conf. appr.	C5 and C6 config.	*T*_degr._ (°C)	Thermal transitions^b^ (°C) for the amphiphilic derivatives	*d*_Lam_ (nm)
(2*R*,3*R*,4*R*,5*R*,6*S*)-Non-8-ene-1,2,3,4,5,6-hexaol (*t*-Man*)	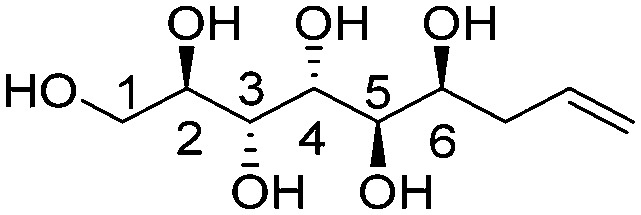	Linear	*R, S*	264	Cr–Hex–Sm–I	3.9
					170 → 183 → 229	
(2*R*,3*S*,4*R*,5*S*,6*R*)-Non-8-ene-1,2,3,4,5,6-hexaol (*t*-Gal*)	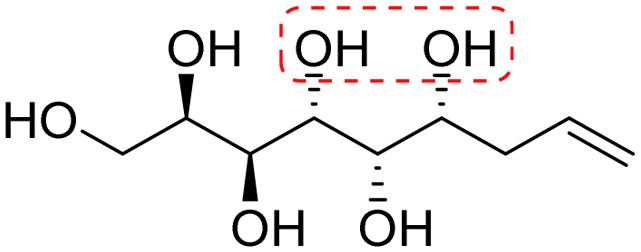	Non-linear	*S, R*	240	Cr–Sm–I	4.1
					118 → 218	
(2*R*,3*R*,4*R*,5*S*,6*R*)-Non-8-ene-1,2,3,4,5,6-hexaol (*t*-Glc*)	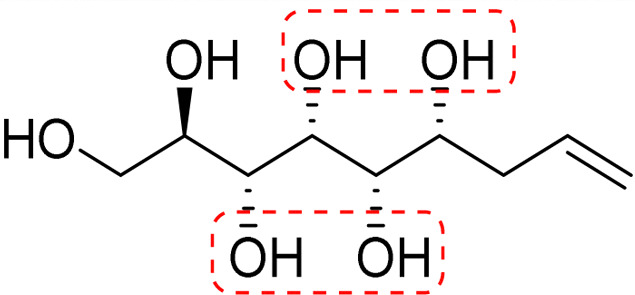	Non-linear	*S, R*	237	Cr–Sm–I	4.2
					103 → 209	
(2*S*,3*S*,4*S*,5*R*)-Oct-7-ene-1,2,3,4,5-pentaol (*t*-Ara*)	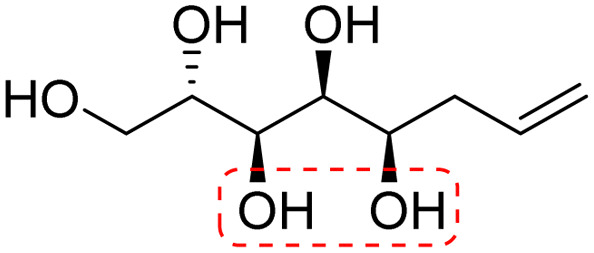	Non-linear	*S, R*	238	Cr–Sm–I	4.0
					85 → 184	
(2*R*,3*S*,4*R*,5*S*,6*S*)-Non-8-ene-1,2,3,4,5,6-hexaol (*e*-Gal*)	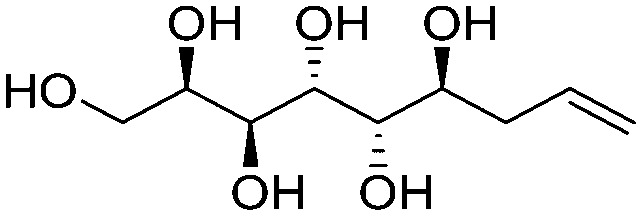	Linear	*S, S*	234	Cr–Cub–Sm–I	3.9
					129 → 165 → 207	
(2*R*,3*R*,4*R*,5*S*,6*S*)-Non-8-ene-1,2,3,4,5,6-hexaol (*e*-Glc*)	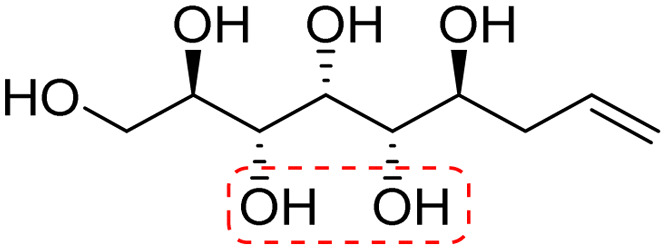	Non-linear	*S, S*	241	Cr–Cub–Sm–I	4.3
					84 → 122 → 210	
(2*S*,3*S*,4*S*,5*S*)-Oct-7-ene-1,2,3,4,5-pentaol (*e*-Ara*)	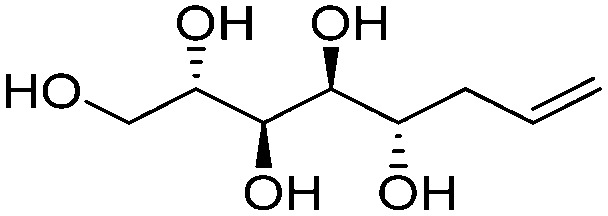	Linear	*S, S*	237	Cr–Cub–Sm–I	4.0
					72 → 124 → 192	
(2*S*,3*S*,4*R*,5*S*,6*R*)-Non-8-ene-2,3,4,5,6-pentaol (*t*-Rha*)	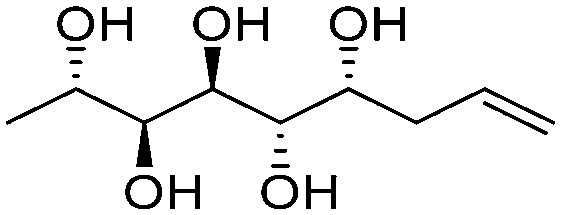	Linear	*S, R*	240	Cr–Cub–stacking transition–Sm–I	3.2
					138 → 149 → 167 → 174	

a 1,3-*syn* relationship of the hydroxyl groups is highlighted when present in the molecule.

b Determined from amphiphilic derivative DCS data, second heating and from the SWAXS data.

Regarding the varying polyol stereochemical configurations, compounds lacking hydroxyl groups in 1,3-*syn* relationship provide a narrower temperature range for the appearance of the Sm phase, whereas compounds containing polyol hydroxyl groups in 1,3-*syn* relationship result in a significantly wider Sm liquid crystalline window. These were verified as reversible isotropic phase transitions by POM, shown as losses of birefringence upon cooling and reheating over the isotropization transition temperature. Only one crystallization and corresponding melting peak upon reheating was observed for *t*-Man*-SC14, *t*-Glc*-SC14, *t*-Gal*-SC14 and *t*-Ara*-SC14. *t*-Gal*-SC14 demonstrates a solid–solid transition upon cooling/reheating at 55–60 °C (Fig. 2b and c). The C14 alkyl tail is considered fully melted at temperatures >80 °C.^66^*e*-Glc*-SC14, *e*-Gal*-SC14, *e*-Ara*-SC14 and *t*-Rha*-SC14 exhibited additional thermally-driven transitions before the Sm transition (Fig. 2). These transitions are reproducible during cooling and reheating. Rearrangements of the cohesive hydrogen-bond connectivity between the polyol blocks affects the Sm phase formation due to stereochemical configurations, and the transition state X-ray analysis discussed later further confirm the additional newly found transitions preceding the Sm phase.

The crystalline melting temperatures (transition temperature from the crystalline to liquid crystalline state) of the amphiphilic compounds follow a pattern similar to that of the melting points of the parent polyols. In this context, linear *versus* non-linear specification is used referring to the parent allylated polyol conformations discussed earlier in the text. The linear hexitol amphiphiles (*t*-Man*-SC14, *e*-Gal*-SC14) have higher crystalline melting points than the non-linear hexitol amphiphiles (*t*-Glc*-SC14, *e*-Glc*-SC14, *t*-Gal*-SC14). The difference in the melting points (transition from crystalline to liquid crystalline state for amphiphilic compounds, melting points for parent polyols) of the non-linear *t*-Ara*-SC14 and linear *e*-Ara*-SC14 derivatives is significantly larger than observed for the parent polyols (Δ*T* = 40.95 °C and Δ*T* = 1.39 °C, respectively). *e*-Ara*-SC14, with a linear parent polyol block, requires a higher temperature to assemble into liquid crystal phase compared to *t*-Ara*-SC14, with 1,3-*syn* positioned hydroxyl groups. The clearing point (isotropization from the liquid crystal state) appears to be much less sensitive to polyol stereochemical configuration than the crystalline melting point. This can be explained by a model developed by van Doren *et al.*,^32^ suggesting that the crystalline melting point corresponds to the collapsing of the original hydrogen bonded network following reformation. The structures (molecular dimers interacting *via* head-to-head hydrogen bonding) then constitute the liquid crystalline species and form smectic layers through stacking, facilitated by the remaining hydroxyl group interactions. At the clearing point, hydrogen bonds can no longer maintain the layers and an isotropic liquid forms.^32^ Thus, the configuration of the hydroxyl groups, together with the conformation of the polyol part, strongly influence the strength of the hydrogen bonding network. This, in turn, determines the melting point of the compound, whereas the clearing point is determined by the strength of the hydrogen bonds left for molecular stacking in Sm layers maintaining the liquid crystalline state. Similar conclusions for alkyl glycosides have been reported previously.^21,67^

In addition to verifying the influence of stereochemical configuration of the hydrophilic head group on solid to liquid crystal transition temperature, the results obtained suggest that the number of hydroxyl groups in the polyol block plays a more critical role on the clearing points than their relative configuration. *t*-Man*-SC14, *t*-Glc*-SC14, *e*-Glc*SC14, *t*-Gal*SC14 and *e*-Gal*-SC14, with six hydroxyl groups, have clearing points well above 200 °C while *t*-Ara*-SC14, *e*-Ara*-SC14 and *t*-Rha*-SC14, with five hydroxyl groups, have clearing points well below it. However, the relevance of the primary hydroxyl group for stability of the Sm liquid crystal phase is demonstrated by the narrow Sm liquid crystal window exhibited by the l-rhamnose derivative *t*-Rha*-SC14, which appears at a temperature range of only 5.1 °C. To our knowledge, no liquid crystal phases have been reported previously for structurally similar open-chain rhamnose derivatives.^20^ Kinetic effects due to dynamic hydrogen bonding of the polyol block are evident with reversible melting enthalpy peak (12.5 kJ mol^−1^) appearing of at 149 °C between the cubic and smectic structures (Fig. 2 and Table 1). Among the analyzed amphiphilic compounds, *t*-Rha*-SC14 has the highest Sm-to-isotropic melting enthalpy (9.7 kJ mol^−1^), indicating that the Sm phase of *t*-Rha*-SC14 is more ordered than in the other compounds studied.

In order to visualize the DSC thermal events, the amphiphilic compounds were subjected to analysis by thermal POM. Generally, thermotropic, single-alkyl tailed carbohydrate mesogens form Sm A phases in the liquid crystal state.^17,62,64,68–70^ The Sm A phase can be identified by observing the defects appearing in the texture, such as focal conics or Maltese crosses which form upon cooling from the isotropic melt.^71,72^ All compounds studied herein formed a Sm phase confirmed by POM before transition to isotropic fluid state (Fig. S2–S11, ESI†). For *t*-Glc*-SC14, *t*-Gal*-SC14 and *t*-Ara*-SC14, the Sm phase transitions were easily visualized by POM, both upon cooling and second heating. *t*-Glc*-SC14, *t*-Gal*-SC14 and *t*-Ara*-SC14 possess hydroxyl groups in 1,3-*syn* relationship within the polyol block structure, leading to non-planar polyol conformation, as discussed earlier in this article. The Sm phase of *t*-Man*-SC14 demonstrated cylindrical defect features at the edges of the sample droplet visualized by POM (Fig. 4c).^73^ The wormlike protrusions show alternating light–dark coloration lengthwise, which are the result of curved smectic layer structures. Curiously, these myelin-like tubular structures have been identified spontaneously forming for lyotropic aqueous 1-*O*-α-, 1-*O*-β- and 1-*S*-α-glucopyranoside systems with alkyl chains of twelve carbons and more, whereas we identify them also in bulk thermotropic system for *t*-Man*-SC14.^33^

**Fig. 3:**
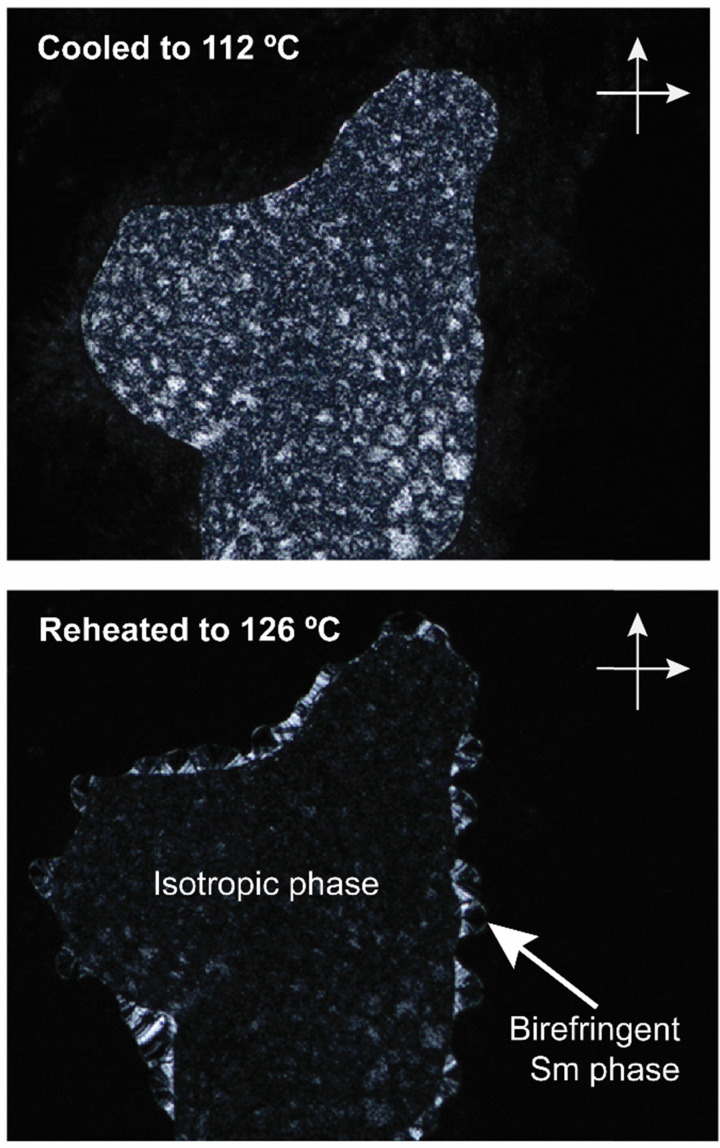
POM images (zoomed-in from ×100 magnification, crossed polarizers) of *e*-Ara*-SC14 upon cooling to 112 °C followed by reheating to 126 °C. Droplet spot-like birefringent texture disappears and the smectic phase starts to form from the edges.

Interestingly, *e*-Gal*-SC14 and *t*-Rha*-SC14 demonstrated viscous behavior upon POM sample handling during heating/cooling from the smectic phase (Fig. S8 and S11, ESI†). Such states did not show sufficient flow, yet the samples were sufficiently soft to be deformed under slight pressing. In addition, *e*-Glc*-SC14, *e*-Gal*-SC14, *e*-Ara*-SC14 and *t*-Rha*-SC14 demonstrated (thermally driven) additional transition peaks in DSC before the Sm transition (Fig. 2). Therefore, we conducted a careful POM analysis for visualization of the transitions at the temperatures indicated by the DSC results. Interestingly, transformation to an isotropic phase before the Sm could be identified for all compounds (*e*-Glc*-SC14, *e*-Gal*-SC14, *e*-Ara*-SC14 and *t*-Rha*-SC14). The POM results are summarized in the ESI.† As an example, compound *e*-Ara*-SC14 showed loss of birefringence upon reheating a spot-like texture from 112 °C to 126 °C (Fig. 3). The Sm phase appeared at 126 °C to the edges of the droplet and the sample melted fully to Sm liquid crystal at 135 °C. For *t*-Rha*-SC14, two different textures appear simultaneously at 165 °C upon cooling. Shearing the sample re-aligns the birefringent areas and, subsequently, a focal conic defect texture with isotropic regions is obtained (Fig. 7b). By cooling to 150 °C, the texture loses birefringence and becomes viscoelastic (Fig. S11, ESI†). Similar textural changes with isotropic appearance were found by Bault *et al.* for galactitol-6-*O*-decyl compound and concluded to result from sample degradation.^36^ The liquid crystal clearing point of galactitol-6-*O*-decyl is at similar low temperature *ca.* 170 °C as for *t*-Rha*-SC14. As TGA did not show any evidence of degradation (*T*_degr._ for *t*-Rha*-SC14 is 240 °C), we looked for an alternative explanation of the unique *t*-Rha*-SC14 liquid crystalline behavior by using SWAXS. The SWAXS results are discussed next for all amphiphilic compounds.

**Fig. 4:**
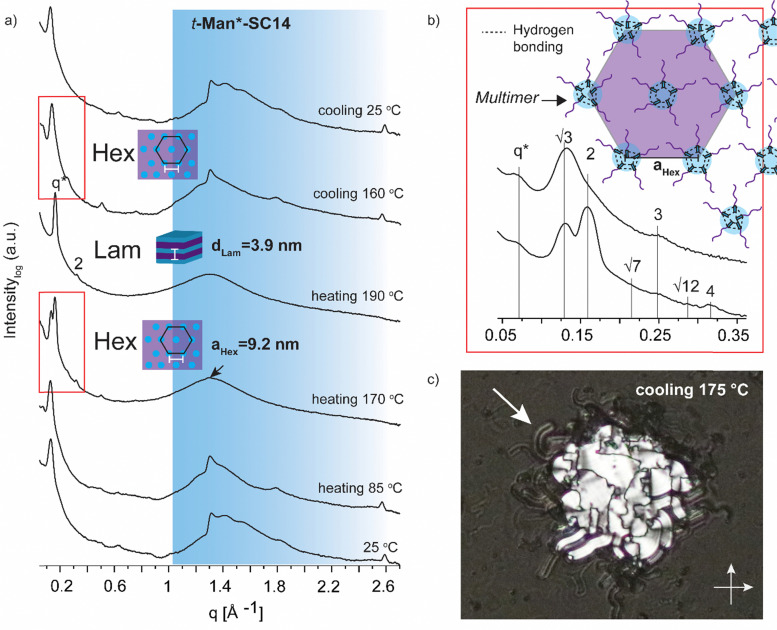
(a) Selected SWAXS profiles for *t*-Man*-SC14 (the black arrow indicates the position of the polyol block crystal lattice peak). Colored area represents the wide-angle region. (b) Zoom-in scattering profile for the hexagonal phase liquid crystal transition at heating to 170 °C with peak indexing and cooling to 160 °C. Schematics for suggested molecular arrangement of multimers is provided *via* polyol head block intermolecular hydrogen bonding. (c) POM (transmission mode, crossed polarizers, zoomed-in from ×20 magnification image) image during cooling indicating macroscopic cylindrical defects for the smectic liquid crystal (white arrow) at the sample droplet edges.

### Temperature dependent amphiphilic structure analysis

Variable temperature small (wide) angle X-ray scattering (SWAXS) was performed to gain insights on the temperature dependent morphological behavior of the liquid crystalline amphiphiles. In the next section, the SWAXS analyses of *t*-Man*-SC14, *t*-Glc*-SC14, *e*-Glc*-SC14, *t*-Gal*-SC14, *e*-Gal*-SC14, *t*-Ara*-SC14, *e*-Ara*-SC14 and *t*-Rha*-SC14 are discussed. Cubic lattice (primitive cubic) parameter calculations, where appropriate, were performed using the Scatter SWAXS data analyzing program (Tables S4–S7, ESI†).^74^

**Fig. 5:**
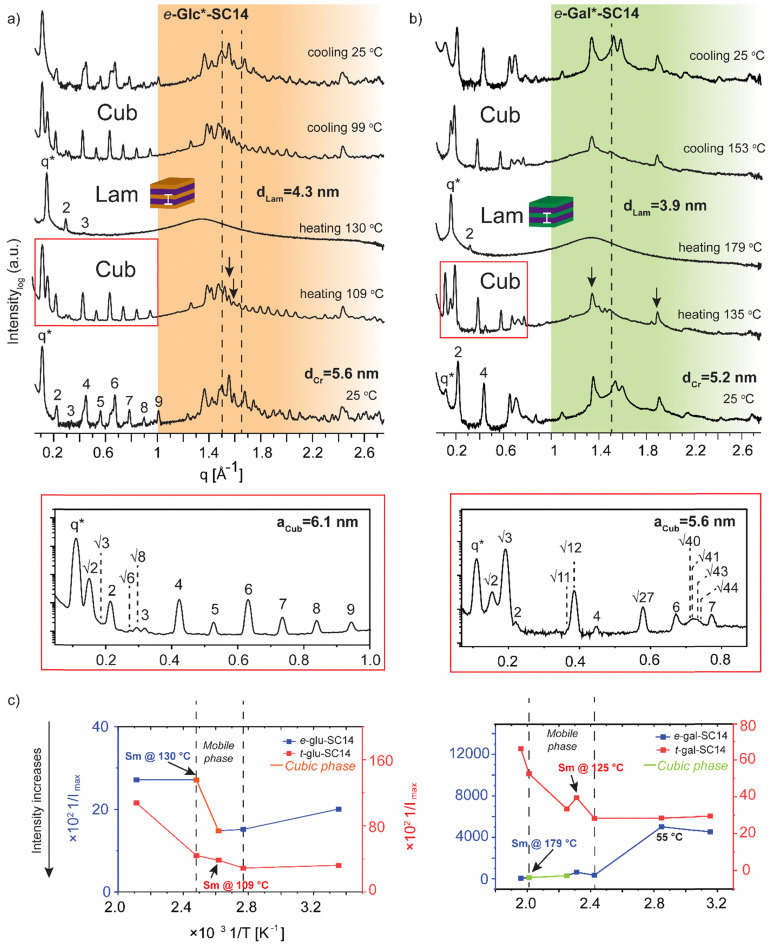
Selected SWAXS profiles for (a) *e*-Glc*-SC14 and (b) *e*-Gal*-SC14 compounds. The dashed lines indicate the position of the crystalline alkyl tail peaks. The black arrows indicate possible polyol block interactions withstanding temperature increase. Colored areas represent the wide-angle regions. (c) The effect of temperature (1/*T*, between 298–463 K) on the maximum scattering intensity (1/*I*_max_) of the main scattering peak for *t*-Glc*-SC14 and *e*-Glc*-SC14, and *t*-Gal*-SC14 and *e*-Gal*-SC14.

All the hexitol based amphiphilic compounds *t*-Man*-SC14, *t*-Glc*-SC14, *e*-Glc*-SC14, *t*-Gal*-SC14 and *e*-Gal*-SC14 studied herein formed the Sm liquid crystal phase with comparable lamella structure size, as confirmed by SWAXS measurements (Fig. 4, 5 and Fig. S12, ESI†). The d-mannose derivative *t*-Man*-SC14 crystallized poorly under the given time frame upon cooling and has a fairly disordered X-ray profile at 25 °C (Fig. 4a). Indications for alkyl tail crystallization at 25 °C are inconclusive with broad peaks in the wide-angle area and major changes in the scattering profile are not observed while heating to 85 °C. Likely, the C14 tail disrupts the polyol head group crystallization upon cooling, leaving the tails unable to crystallize during the process. A hexagonal cylindrical structure is obtained at 170 °C while approaching the Sm phase with Bragg peak series: q*, √3, 2, √7, 3, √12, 4 (Fig. 4b). Curiously, this transition did not show in the DSC experiment (Fig. 2). It reforms during cooling and is detectable with similar peak pattern at 160 °C. The cylinder lattice parameter (*a*_Hex_) is calculated as *a*_Hex_ = 9.2 nm. The exceptionally large domain period is suggested to origin from multimers composed of clustered hydrogen bonded polyol blocks (Fig. 4b). The multimers pack in columns with hexagonal arrangement in the transition state. A peak at 1.3 Å^−1^ at 170 °C is assigned to remaining mannose block hydrogen bonded network within the multimer core (Fig. 4a).^51^ Only Sm phases have been observed for mono alkylated polyol derivatives.^75^ Hexagonal phases have been detected for carbohydrate derivatives possessing dual carbon tails which lead to disk-like multimer formation and lack of Sm phase formation.^31^ With singular and long enough hydrocarbon tail to obtain the larger volume fraction (and free volume) from the polyol, *t*-Man*-SC14 forms a hexagonal cylinder transition structure at 170 °C (hydrogen bonds are highly dynamic at this high enough temperature). SAXS data supports the formation of phase separated polyol block core structure without order with non-interdigitated alkyl tails enabling the particularly large *a*_Hex_ lattice parameter. The Hex phase for carbohydrate derivatives has been described to form from disk-like multimers containing five molecules and this analogy is used in schematics in Fig. 4b illustrating the molecular arrangement of multimers in the transition structure.^31^ At 170 °C, the primary peak for hexagonal structure is already diminishing, and the tertiary peak obtains highest intensity indicating the merging lamella phase primary peak. At 190 °C, the Sm liquid crystal layered structure is confirmed by two Bragg peaks (q* and 2q*). Domain period for the lamella morphology can be calculated from the SAXS data using the expression *d* = 2π/q*, where q* is the position of the principal SAXS peak giving lamella spacing of *d*_Lam_ = 3.9 nm. A partly interdigitated molecular layering is suggested for the Sm phase, considering the reported approximate estimation of block sizes based on block melt densities.^17,36,76^

In contrast to *t*-Man*-SC14, compounds *t*-Glc*-SC14 and *t*-Gal*-SC14 show highly layered (crystalline) structures both at small and wide scattering angles at 25 °C (Fig. S12, ESI†). Compounds *t*-Glc*-SC14, *e*-Glc*-SC14 and *t*-Gal*-SC14 show two WAXS peaks (1.5 and 1.65 Å^−1^) corresponding to the alkyl tail crystallized orthorhombic structure (Fig. 5a and Fig. S12, ESI†).^77^ Only one peak at 1.51 Å^−1^ for *e*-Gal*-SC14 alkyl crystallization is detected, implying a hexagonal solid structure for the tail.^77^ The glucose-derived compound domain sizes are slightly larger than the corresponding mannose and galactose derivatives, both in solid and in Sm liquid crystal state (for example *e*-Glc*-SC14*d*_Lam_ = 4.3 nm and *e*-Gal*-SC14*d*_Lam_ = 3.9 nm). Interestingly, *e*-Glc*-SC14 forms an intermediate cubic structure (scattering peak series: q*, √2, √3, 2, √6, √8, 3, 4, 5, 6, 7, 8, 9) at 109 °C and *e*-Gal*-SC14 at 135 °C with peak spacing ratio series of q*, √2, √3, 2, √11, √12, 4, √27, 6, √40, √41, √43, √44, 7. To get an estimate value of the size of the cubic structures, we assume a primitive cubic lattice structure for both and calculate the lattice size of *a*_Cub_ = 6.1 nm for *e*-Glc*-SC14 and *a*_Cub_ = 5.6 nm for *e*-Gal*-SC14 for the compounds (Fig. 5a, b and Tables S4 and S5, ESI†). The WAXS area of the cubic transition shows rearranged peaks from polyol block hydrogen bonding, yet resistance to the temperature increase for breaking of polyol structure interactions is also seen with prevailing WAXS peaks, indicated in Fig. 5a for both *e*-Glc*-SC14 and *e*-Gal*-SC14. The earlier discussed POM observations confirm the cubic transitions for *e*-Glc*-SC14 and *e*-Gal*-SC14 with a loss of birefringence in the sample upon reheating. Also, DSC showed reproducible endothermic peaks before the Sm phase (Fig. 2b and c). Both POM and DSC (1st heating cycle) captured a highly colorful solid–solid phase transition at 108 °C for *t*-Gal*-SC14. Two high intensity wide angle peaks from alkyl tail orthorhombic crystalline structure disappear at 108 °C according to WAXS data (Fig. S12, ESI†). The polyol hydrogen bonded network is forced to rearrange, as evidenced by the apparent peak shuffling at both small and wide scattering angles. *e*-Gal*-SC14 reorganizes with a steep increase in *I*_max_ during heating to 108 °C (Fig. 5c). The stereochemistry of *e*-Gal* (Fig. 1) strongly influences the compound melting temperature, enabling the cubic phase formation with dynamic polyol block hydrogen bonding (indicated as a mobile phase in Fig. 5c), before melting into Sm phase. Prevailing polyol block crystalline peaks at 135 °C are seen in the corresponding scattering data in Fig. 5b. Interestingly, the *e*-Gal-SC14 primary peak at 25 °C is also low in intensity with disordered starting structures, as also observed for *t*-Man*-SC14. The linear polyol conformation of the *e*-Gal*-SC14 compound due to polyol block hydroxyl group 1,3-*syn* relations could induce similar solid phase behavior as seen with *t*-Man*-SC14 (Table S1, ESI†).

The influence of temperature on block copolymer structure formation can be monitored by plotting the inverse maximum intensity (*I*_max_) of the main scattering peak (q*) against the inverse temperature.^78^ By applying this method to the monosaccharide based amphiphilic compounds, a phase range between the melting of solid structures and the Sm liquid crystalline phase can be demonstrated (Fig. 5c). The glucose derived diastereomers have little variation in scattering intensity when melting into liquid crystal phase, but for galactose derived diastereomers the *e*-Gal*-SC14 rather disordered structure melts first into a cubic phase and further into Sm phase, with tenfold scattering intensity compared to *t*-Gal*-SC14.

**Fig. 6:**
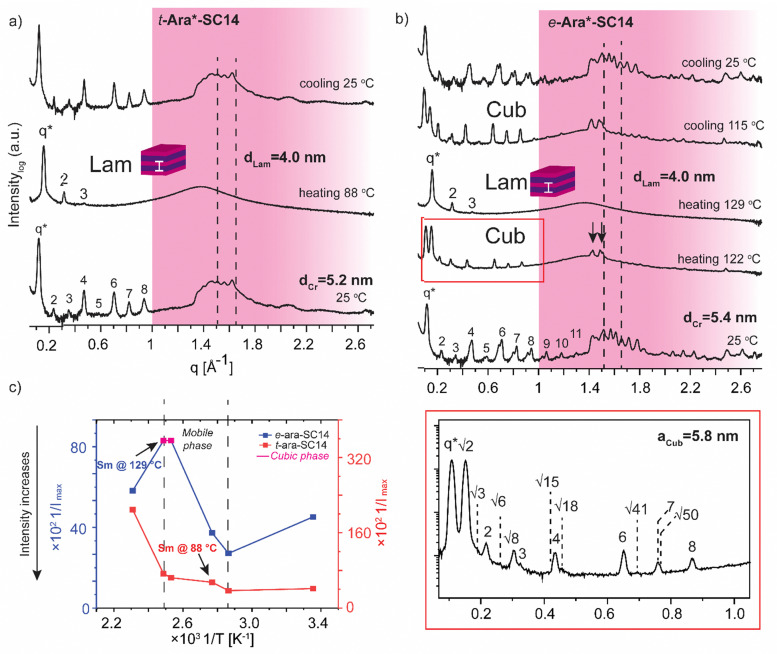
Selected SWAXS profiles of the arabitol diastereomers (a) *t*-Ara*-SC14 and (b) *e*-Ara*-SC14. Dashed lines indicate the position of the alkyl tail crystal peaks and black arrows the remaining polyol crystals peaks. Colored areas represent the wide-angle regions. (c) The effect of temperature (1/*T*, between 298–433 K) on the maximum intensity (1/*I*_max_) of the main scattering peak for both compounds.

The l-arabinose and l-rhamnose derived compounds *t*-Ara*-SC14, *e*-Ara*-SC14 and *t*-Rha*-SC14 are pentitol based structures with five hydroxyl groups. Moreover, the l-rhamnose derived *t*-Rha*-SC14 is the only compound studied here possessing a hydrophobic functional group at both ends of the molecule (Fig. 1). Similar temperature dependent SWAXS behavior, as observed for the l-glucose derived compounds, were also characteristic for the arabinose derived compounds *t*-Ara*-SC14 and *e*-Ara*-SC14 (Fig. 6). Compound *t*-Ara*-SC14 melts into a Sm phase at 88 °C while the *e*-Ara*-SC14 shows a cubic phase transition at 122 °C with scattering spacing ratios: q*, √2, √3, 2, √6, √8, 3, √15, 4, √18, 6, √41, 7, √50, 8 and *a*_Cub_ = 5.8 nm before transition to the Sm phase at 129 °C (Fig. 6a, b and Fig. S6, ESI†). Formation of the *e*-Ara*-SC14 cubic structure can be confirmed also upon cooling at 115 °C with similar scattering pattern. Compound *e*-Ara*-SC14 shows an oblique polycrystalline structure at 25 °C similar to *e*-Glc*-SC14 by showing a double peak pattern at SAXS region and several sharp high intensity peaks in the WAXS area. Unfortunately, alkyl tail crystallinity is difficult to observe underneath the polyol crystal peaks. Temperature dependent *I*_max_ analysis (Fig. 6c) revealed delicate phase formation for *e*-Ara*-SC14 as the intensity increases upon melting of the oblique structures till 76 °C, following a steep intensity decrease at 122 °C by the formation of cubic phase. Interestingly, at 122 °C two intense WAXS peaks assigned to polyol hydrogen bonding remain demonstrating the apparent mobility of the hydrogen bonding network of the pentitol rod blocks. POM images shown in Fig. 3 for *e*-Ara*-SC14 can be explained by the formation of an isotropic 3D network of hydrogen bonded pentitol blocks around 122 °C. The somewhat smooth *I*_max_ profile for the *t*-Ara*-SC14 compound resembles that observed for *t*-Glc*-SC14 compound. The fused WAXS area peaks at 25 °C indicate poor polyol block crystallization and, therefore, the compounds melt at lower temperature compared to *e*-Ara*-SC14 where sharp crystalline peaks in solid state are seen before heating.

**Fig. 7:**
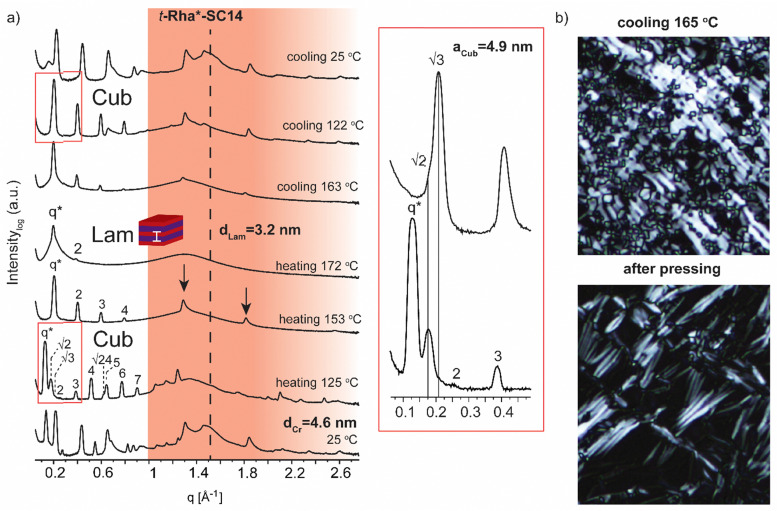
(a) Selected SWAXS profiles for the rhamnitol derivative *t*-Rha*-SC14. Colored area represents the wide-angle region. Dashed lines indicate the position of alkyl tail crystal peaks. Zoom-in scattering profile for cubic phase transition upon heating to 125 °C and cooling to 122 °C. (b) POM images (cooling at 165 °C) before and after shearing the sample by pressing. Black arrows show the maintained polyol block hydrogen bonding at 158 °C.

Finally, we address the SWAXS results of *t*-Rha*-SC14 (Fig. 7). Specifically, the effect of the absence of the polyol hydroxyl head group on the temperature dependent structure formation is demonstrated with *t*-Rha*-SC14. The Sm liquid crystal phase (at 172 °C with q* and 2 scattering peaks) forms through a cubic structure at 125 °C with scattering peak series: q*, √2, √3, 2, 3, 4, √24, 5, 6 and 7. (Fig. 7a, Table S6, ESI†) Interestingly, a transition state, identified also with DSC (reproducible significant enthalpy peak) between the Cub and the Sm phase, forms through a specific polyol block hydrogen bonding, identified from the WAXS area (Fig. 7a arrows indicate rearranged polyol block stacking). At 153 °C, a clean lamella peak series becomes visible (q*, 2, 3, 4) and, curiously, with two sharp WAXS peaks remaining (1.29 and 1.81 Å^−1^). Cubic structure reformation is detected from the SAXS data upon cooling to 122 °C. The lattice parameter is calculated according to primitive cubic space group as *a*_Cub_ = 4.9 nm. A single peak at 1.51 Å^−1^ suggests the formation of a hexagonal crystal lattice by the alkyl tail in solid state before heating. The WAXS peaks diffuse at 172 °C into a single broad peak and primary peak width broadening is also observed. Since the Sm phase temperature region is very narrow, possibly because of the end methyl group distorting the polyol hydrogen bonding, the Sm phase transition into isotropic fluid broadens the primary peak width. As the Sm phase domain is as small as *d*_Lam_ = 3.2 nm, the structure forms by terminal methyl group interdigitation into the hydrophobic alkyl tail layer. The hydrogen bonded polyol domain structure seems to be kinetically hindered after cubic structure as sharp peak pattern is seen at the WAXS area together with the lamella peak pattern appearing clearly at the SAXS area (Fig. 7a). Compound *t*-Rha*-SC14 shows viscoelastic phase behavior through visual observation during POM upon heating/cooling cycles and a loss of birefringence at 115 °C during cooling (Fig. S11, ESI†). The SWAXS analysis confirms reversible temperature dependent dynamic hydrogen bonding for the *t*-Rha* block at 153 °C upon heating and at 163 °C upon cooling. Some of the hydrogen bonds rearrange, inflicting kinetic effects seen both in DSC and SAXS data (Fig. 2 and 7). Sm phase domain spacing *d*_Lam_ = 3.2 nm for the *t*-Rha*-SC14 is the smallest spacing obtained for the compounds studied in this work and being the best to fit into the suggested alkyl tail interdigitated molecular lamella model.

Carbohydrate based compounds possessing double or triple alkyl chains linked to the polyol block are known to form hexagonal cylinder and cubic phases.^79^ Therein, the large volume fractions of the hydrophobic parts shape the curved interface separating the phase segregating blocks within the molecule. Cubic isotropic mesophases can possess interwoven networks of branched columns or consist of closed micelles arranged in a cubic lattice structure.^40,80^ The compounds studied herein are non-branched by molecular shape and, interestingly, polyol specific stereo-configurations can direct the curved interface formation.

**Fig. 8:**
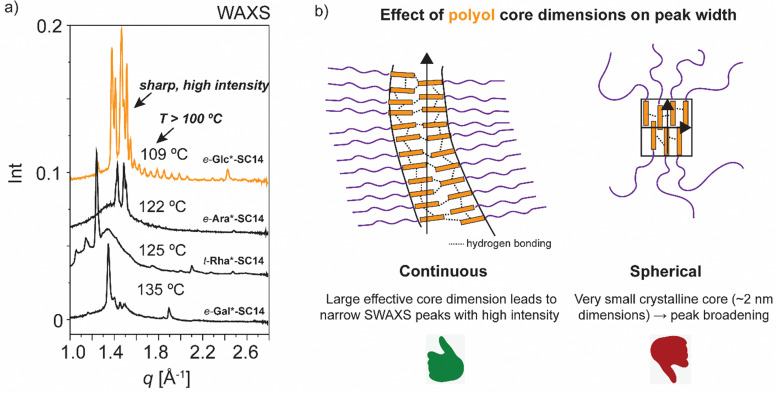
(a) Selected WAXS data for *e*-Glc*-SC14, *e*-Gal*-SC14, *e*-Ara*-SC14 and *t*-Rha*-SC14 cubic phases upon heating. (b) Schematics describing the suggested possible cubic phase structure, favoring the continuous core structure formation using *e*-Glc*-SC14 as an example compound. The small crystalline domains forming a spherical cubic phase would show peak broadening in SWAXS, not seen for the compounds in question.

High intensity and sharp WAXS area peak patterns from cubic phase forming highly ordered polyol block crystals are seen at the cubic transition temperature (Fig. 8a). In order to maintain the hydrogen-bonded network, hence the polyol crystalline structure indicated by WAXS, the cubic phase would need to develop without the polyol block interactions getting irreversibly disrupted. Similar to *t*-Man*-SC14 with hexagonal closed packed cylinder structure preceding the Sm phase, the polyol block interactions can be maintained through a bicontinuous cubic phase formation. Therein the polyol core interactions are preserved and they rearrange into branched layers connected to neighboring layers upon heating.^9^

We suggest a co-continuous structure for *e*-Glc*-SC14, *e*-Gal*-SC14, *e*-Ara*-SC14 and *t*-Rha*-SC14 cubic phases, because such a rich pattern of polyol crystalline reflections obtained, would not be expected for spherical nanosegregated domains classically explaining the cubic self-assembly. Fig. 8a introduces selected cubic structure WAXS area profiles for compounds studied herein. According to Scherrer formulation, intensive X-ray peak broadening covering small and wide-angle range, is evident for small crystallite sizes (<200 nm).^81^ A very small orthorhombic crystallite structure size was obtained for *t*-Man* (space group *P*2_1_2_1_2, unit cell parameters: *a* 21.1909(5) Å, *b* 8.8667(2) Å, *c* 5.55223(12) Å) from previous single crystal X-ray diffraction analysis.^52^ Therefore, if a spherical cubic phase transition would occur for cubic phase forming compounds in this study, most likely the core would form from a few molecules forming a very small polyol crystals. Yet, both SAXS and WAXS peak patterns for cubic phase forming compounds (Fig. 8a) demonstrate narrow peaks with high intensity from polyol block inter- and intramolecular hydrogen bonding in temperatures over 100 °C. Therefore, we suggest the formation of polyol block 3D continuous network preceding the smectic phase transition for the compounds. An illustrated example of this is given in Fig. 8b for *e*-Glc*-SC14. The branching can occur through connected polyol core cylinders or as ribbons of crystalline aggregates (Fig. 8b). For compound *t*-Rha*-SC14, with *S, R* configuration at the interface, cubic phase interpretation is more complex due to polyol head nonpolar methyl group effects on self-assembly (Table 2).

This comprehensive thermal analysis of mesoscopic structures of monosaccharide based amphiphilic compounds interestingly shows that the minimal energy conformations of the polyol blocks influence the overall nanophase separation. Hydrogen bonds break and reform at temperatures above 100 °C under reorientational motion^10^ and enable the rich phase behavior from cubic/cylindrical to Sm liquid crystal phases seen for particular compounds. Of the compounds studied *t*-Glc*-SC14, *t*-Gal*-SC14 and *t*-Ara*-SC14 with non-linear polyol blocks, formed merely the Sm liquid crystal phase. The overall linear shape of the amphiphilic block molecules and the balanced hydrophobic *vs.* hydrophilic volume fractions support Sm phase formation. On the other hand, *t*-Man*-SC14, *e*-Glc*-SC14, *e*-Gal*-SC14, *e*-Ara*-SC14 and *t*-Rha*-SC14 with linear polyol blocks demonstrate a curved interface structure hexagonal or cubic phase formation before the Sm phase upon heating. In the case of the hexagonal liquid crystal phase of *t*-Man*-SC14, the monoalkylated molecular shape does not support non-lamellar structure formation, yet the hexagonal transition structure could be explained by the prevailing polyol interaction forming the polar cylinder core.^9^ Linear hexitol blocks containing *t*-Man*-SC14 and *e*-Gal*-SC14 show smaller lamella spacings with higher crystalline melting temperatures than the non-linear *t*- and *e*-Glc*-SC14 and *t*-Gal*-SC14 (Table 2). The polyol block molecule packing for *t*-Man*-SC14 and *e*-Gal*-SC14 compounds is enhanced, and the coil parts are forced to stretch, enabling more efficient interdigitated block structures in Sm phase. Pentitol *t*-Ara*-SC14 with smaller rod block size, and with a non-linear approximated minimal conformation, still obtains a larger lamella domain spacing than the linear hexitol containing *t*-Man*-SC14 (Table 2). Of the pentitol-derived compounds *t*-Ara*-SC14 and *e*-Ara*-SC14, the latter possessing linear minimum energy conformation in its polyol block, has the lower liquid crystal transition temperature. Even though the linear conformation of the *t*-Man* block can hinder the kinetics of alkyl tail crystallization of the molecules, in comparison with the non-linear *t*-Glc* and *e*-Glc* blocks and their analogues, highly crystalline structures were developed for the glucose derived compounds at 25 °C, whereas *t*-Man*-SC14 remains disordered. Upon heating, minor changes in the *e*-Glc*-SC14 intramolecular hydrogen bonding network, in contrast to the *t*-Glc*-SC14 (one 1,3-*syn* hydroxyl group pair *versus* two pairs), are sufficient to promote a significant structural transition into a cubic phase for *e*-Glc*-SC14 prior to its further transition into the Sm phase. The pentitol containing *t*-Rha*-SC14 is a molecule structurally differing from the others, because of its more complex triblock oligomer-like architecture, wherein the linear polyol block is positioned in the middle of the molecule. This enables the formation of a highly layered, non-flowing lamella intermediate structure between the cubic and the smectic phase.

*e*-Glc*-SC14, *e*-Gal*-SC14 and *e*-Ara*-SC14 possess the same relative configurations at the C5 and C6 stereocenters at the polar-nonpolar interface, separating the polyol and the coil blocks and uptake the cubic intermediate phase before the smectic (Table 2). Apparently linear polyol block conformation can aid in curved interface structure formation for *e*-Gal*-SC14 and *e*-Ara*-SC14, yet *e*-Glc*-SC14 polyol block is described nonlinear according to computed geometry optimizations and still forms the cubic phase. Therefore, we explain the cubic phase formation of *e*-Gal*-SC14 solely with the similar stereochemistry with compounds, *e*-Gal*-SC14 and *e*-Ara*-SC14 (*S, S* in C5 and C6) emphasizing the effect of stereocenters at the polar-nonpolar interface on cubic phase formation for the compounds in question.

A 3D co-continuous cubic phase formation is suggested for *e*-Glc*-SC14, *e*-Gal*-SC14, *e*-Ara*-SC14 and *t*-Rha*-SC14 due to the persistent, yet changing hydrogen bonding network, and identified by X-ray studies. Cubic structure maintains a reformed sharp and high intensity peaks in the WAXS region, assigned to the dynamic hydrogen bonded structures of the polar polyol blocks. A significant difference in the solid-to-liquid crystal WAXS pattern appearance is also evident between the diastereomers of the same parent monosaccharide (Fig. 5, 6 and Fig. S12, ESI†), demonstrating the sensitivity of the stereochemical effects of the polyol head to phase segregation, building into varying nanostructures. The long-range stacking of polyol blocks can further evolve into a 3D network if the flexibility/thermal vibration of the long alkyl chain is yet restricted with insufficient temperature increase and the chain is unable to crowd the whole volume around the polyol core. Simulations of carbohydrate based amphiphile lyotropics have shown the effects of compound stereochemistry on non-lamellar structure formation.^82,83^ For *e*-Glc*-SC14, *e*-Gal*-SC14 and *t*-Rha*-SC14, the cubic transition is stable for over 20 °C range. Temperature variations trigger unique optical and mechanical properties from isotropic and viscous cubic phases into a birefringent Sm fluid. These properties are highly attractive for controlled/tunable stimuli-induced material design enabled by structural changes through self-assembly.^40^ A clear distinction between spherical and bicontinuous cubic symmetries for *e*-Glc*-SC14, *e*-Gal*-SC14, *e*-Ara*-SC14 and *t*-Rha*-SC14 is not possible on the basis of the obtained results and requires additional measurements *e.g.*, by single crystal X-ray diffraction. All phase transitions for the compounds studied in this work (to smectic and to cubic → smectic) are thermoreversible, consistent with equilibrium phases.

## Conclusions

A series of amphiphilic block molecules with varying hydrophilic monosaccharide-based polyol blocks and well-defined stereocenters combined with tetradecyl alkyl blocks were synthesized by UV-induced click-chemistry. Temperature dependent self-assemblies were characterized by DSC, POM and SWAXS techniques. The stereochemistry of the parent polyols influenced the complexity of the self-assemblies and strongly controlled their thermal and liquid crystalline behavior. All compounds showed low temperature crystalline phases, smectic phases, and isotropic phases. Interestingly, cubic phases were observed for *e*-Glc*-SC14, *e*-Gal*-SC14, *e*-Ara*-SC14 before entering the smectic phase. They consist of the same C5 and C6 relative configurations near the interface separating the polar polyol and the nonpolar hydrocarbon tail blocks, unlike the compositions that did not lead to the cubic phase. This is rationalized by the fact that stereocenters nearest to the junction point are most sensitive under the influence of thermal fluctuations and hydrogen bonding network rearrangements. As the temperature further increases over the cubic phase transition temperature, more hydrogen bonds are broken enhancing the phase segregation of the immiscible blocks of the amphiphile into a flat interface, specific property for carbohydrate amphiphilic block molecules through polyol hydrogen bonding. Our results provide steps towards novel carbohydrate based high-*χ* block molecules that are expected to pave the way to understand and optimize the effects of stereochemistry on the resulting self-assemblies.

## Author contributions

I. M., J. M. and M. L. contributed to synthesis work and/or analysis of the synthesis products. T. S. and A. F. conducted the computational modelling. R. L., T. S. S., O. R., O. I. all contributed to supervision and conception of the work. All authors contributed to the writing of the manuscript draft as well as to reviewing and editing of the final version. The manuscript was written through contributions of all authors. All authors have given approval to the final version of the manuscript.

## Conflicts of interest

There are no conflicts of interest to declare.

## Supplementary Material

SM-019-D3SM00939D-s001

## References

[cit1] Whitesides G. M., Grzybowski B. (2002). Science.

[cit2] Ikkala O. , Houbenov N. and Rannou P., in Handbook of Liquid Crystals, ed. P. Goodby, J. W. Collings, P. J. Kato, T. Tschierske, C. Gleeson and H. Raynes, Wiley-VCH Verlag GmbH & Co, Series Eds., 2014, pp. 541–598

[cit3] Self-Assembly: From Surfactants to Nanoparticles, ed. R. Nagarajan, Wiley, 2018

[cit4] van Genabeek B., Lamers B. A. G., Hawker C. J., Meijer E. W., Gutekunst W. R., Schmidt B. V. K. J. (2021). J. Polym. Sci..

[cit5] Leibler L. (1980). Macromolecules.

[cit6] Bates C. M., Bates F. S. (2017). Macromolecules.

[cit7] Sinturel C., Bates F. S., Hillmyer M. A. (2015). ACS Macro Lett..

[cit8] Olsen B. D., Segalman R. A. (2008). Mater. Sci. Eng., R.

[cit9] Lee M., Cho B.-K., Kim H., Yoon J.-Y., Zin W.-C. (1998). J. Am. Chem. Soc..

[cit10] Paleos C. M., Tsiourvas D. (1995). Angew. Chem., Int. Ed. Engl..

[cit11] Luo Y., Montarnal D., Treat N. J., Hustad P. D., Christianson M. D., Kramer E. J., Fredrickson G. H., Hawker C. J. (2015). ACS Macro Lett..

[cit12] Majoinen J., Bouilhac C., Rannou P., Borsali R. (2022). ACS Macro Lett..

[cit13] Isono T., Komaki R., Lee C., Kawakami N., Ree B. J., Watanabe K., Yoshida K., Mamiya H., Yamamoto T., Borsali R., Tajima K., Satoh T. (2020). Commun. Chem..

[cit14] Cushen J. D., Shanmuganathan K., Janes D. W., Willson C. G., Ellison C. J. (2014). ACS Macro Lett..

[cit15] Nowak S. R., Lachmayr K. K., Yager K. G., Sita L. R. (2021). Angew. Chem., Int. Ed..

[cit16] Bates M. W., Barbon S. M., Levi A. E., Lewis R. M. I. I. I., Beech H. K., Vonk K. M., Zhang C., Fredrickson G. H., Hawker C. J., Bates C. M. (2020). ACS Macro Lett..

[cit17] Barreda L., Shen Z., Chen Q. P., Lodge T. P., Siepmann J. I., Hillmyer M. A. (2019). Nano Lett..

[cit18] Singh M. K., Jayaraman N. (2009). J. Indian Inst. Sci..

[cit19] Goodby J. W., Görtz V., Cowling S. J., Mackenzie G., Martin P., Plusquellec D., Benvegnu T., Boullanger P., Lafont D., Queneau Y., Chambert S., Fitremann J. (2007). Chem. Soc. Rev..

[cit20] van Doren H. A., Smits E., Pestman J. M., Engberts J. B. F. N., Kellogg R. M. (2000). Chem. Soc. Rev..

[cit21] Hashim R., Zahid N. I., Velayutham T.
S., Aripin N. F. K., Ogawa S., Sugimura A. (2018). J. Oleo Sci..

[cit22] Goodby J. W., Saez I. M., Cowling S. J., Görtz V., Draper M., Hall A. W., Sia S., Cosquer G., Lee S. E., Raynes E. P. (2008). Angew. Chem., Int. Ed..

[cit23] Fuhrhop J. H., Helfrich W. (1993). Chem. Rev..

[cit24] Pfannemuller B., Welte W., Chin E., Goodby J. W. (1986). Liq. Cryst..

[cit25] Chaveriat L., Stasik I., Demailly G., Beaupère D. (2004). Carbohydr. Res..

[cit26] Cook A. G., Wardell J. L., Imrie C. T. (2011). Chem. Phys. Lipids.

[cit27] Stasik I., Gottis S., Falentin-Daudré C., Meyer C. (2014). Carbohydr. Res..

[cit28] Moore J. E., McCoy T. M., Marlow J. B., Pottage M. J., Mudie S. T., Pearson G. R., Wilkinson B. L., Tabor R. F. (2019). J. Colloid Interface Sci..

[cit29] Tschierske C. (2002). Curr. Opin. Colloid Interface Sci..

[cit30] van Doren H., Buma T. J., Kellogg R. M., Wynberg H. (1988). J. Chem. Soc., Chem. Commun..

[cit31] Praefcke K., Levelut A.-M., Kohne B., Eckert A. (1989). Liq. Cryst..

[cit32] van Doren H., Van Der Geest R., Keuning C. A., Kellogg R. M., Wynberg H. (1989). Liq. Cryst..

[cit33] Jeffrey G. A., Wingert L. M. (1992). Liq. Cryst..

[cit34] Letellier P., Ewing D. F., Goodby J. W., Haley J., Kelly S. M., Mackenzie G. (1997). Liq. Cryst..

[cit35] Goodby J. W., Haley J. A., Watson M. J., Mackenzie G., Kelly S. M., Letellier P., Douillet O., Gode P., Goethals G., Ronco G., Villa P. (1997). Liq. Cryst..

[cit36] Bault P., Gode P., Goethals G., Goodby J. W., Haley J. A., Kelly S. M., Mehl G. H., Ronco G., Villa P. (1998). Liq. Cryst..

[cit37] Bault P., Gode P., Goethals G., Goodby J. W., Haley J. A., Kelly S. M., Mehl G. H., Villa P. (1999). Liq. Cryst..

[cit38] Poppe S., Cheng X., Chen C., Zeng X., Zhang R., Liu F., Ungar G., Tschierske C. (2020). J. Am. Chem. Soc..

[cit39] Chen C., Poppe M., Poppe S., Tschierske C., Liu F. (2020). Angew. Chem., Int. Ed..

[cit40] Kutsumizu S. (2012). Isr. J. Chem..

[cit41] Fischer S., Fischer H., Diele S., Pelzl G., Jankowski K., Schmidt R. R., Vill V. (1994). Liq. Cryst..

[cit42] Kutsumizu S. (2002). Curr. Opin. Solid State Mater. Sci..

[cit43] Impéror-Clerc M. (2005). Curr. Opin. Colloid Interface Sci..

[cit44] Tschierske C. (2002). Curr. Opin. Colloid Interface Sci..

[cit45] Mannock D. A., Harper P. E., Gruner S. M., McElhaney R. N. (2001). Chem. Phys. Lipids.

[cit46] Tenchova R., Tenchov B., Hinz H.-J., Quinn P. J. (1996). Liq. Cryst..

[cit47] Liew C. Y., Salim M., Zahid N. I., Hashim R. (2015). RSC Adv..

[cit48] Mannock D. A., Collins M. D., Kreichbaum M., Harper P. E., Gruner S. M., McElhaney R. N. (2007). Chem. Phys. Lipids.

[cit49] Schmid W., Whitesides G. M. (1991). J. Am. Chem. Soc..

[cit50] Kim E., Gordon D. M., Schmid W., Whitesides G. M. (1993). J. Org. Chem..

[cit51] Mattsson I., Lahtinen M., Peuronen A., Sau A., Gunell A., Saloranta-Simell T., Leino R. (2018). Cryst. Growth Des..

[cit52] Saloranta T., Peuronen A., Dieterich J. M., Ruokolainen J., Lahtinen M., Leino R. (2016). Cryst. Growth Des..

[cit53] Saloranta T., Müller C., Vogt D., Leino R. (2008). Chem. – Eur. J..

[cit54] Mattsson I., Sitdikov R., Gunell A. C. M., Lahtinen M., Saloranta-Simell T., Leino R. (2020). RSC Adv..

[cit55] Ogawa S., Honda K., Tsubomura T., Totani K., Takahashi I., Hara S. (2018). Chem. Phys. Lipids.

[cit56] Crich D., Pirrone M. G., Gysin M., Haldimann K., Hobbie S. N., Vasella A. (2020). J. Org. Chem..

[cit57] Lewis D., Angyal S. J. (1989). J. Chem. Soc., Perkin Trans. 2.

[cit58] Gillies D. G., Lewis D. (1985). J. Chem. Soc., Perkin Trans. 2.

[cit59] Lewis D. (1986). J. Chem. Soc., Perkin Trans. 2.

[cit60] Hawkes G. E., Lewis D. (1984). J. Chem. Soc., Perkin Trans. 2.

[cit61] Matsumori N., Kaneno D., Murata M., Nakamura H., Tachibana K. (1999). J. Org. Chem..

[cit62] Vill V., Hashim R. (2002). Curr. Opin. Colloid Interface Sci..

[cit63] Milkereit G., Gerber S., Brandenburg K., Morr M., Vill V. (2005). Chem. Phys. Lipids.

[cit64] Liao G., Zewe S. K., Hagerty J., Hashim R., Abeygunaratne S., Vill V., Jákli A. (2006). Liq. Cryst..

[cit65] Nguan H. S., Heidelberg T., Hashim R., Tiddy G. J. T. (2010). Liq. Cryst..

[cit66] Wang F. C., Miyazaki Y., Marangoni A. G. (2018). Cryst. Growth Des..

[cit67] Ericsson C. A., Ericsson L. C., Kocherbitov V., Söderman O., Ulvenlund S. (2005). Phys. Chem. Chem. Phys..

[cit68] Milkereit G., Garamus V. M., Yamashita J., Hato M., Morr M., Vill V. (2005). J. Phys. Chem. B.

[cit69] Paleos C. M., Tsiourvas D. (2001). Liq. Cryst..

[cit70] Vill V., von Minden H. M., Koch M. H. J., Seydel U., Brandenburg K. (2000). Chem. Phys. Lipids.

[cit71] Chaveriat L., Meyer C., Beaupère D., Demailly G., Stasik I. (2008). J. Mol. Liq..

[cit72] Ericsson C. A., Ericsson L. C., Ulvenlund S. (2005). Carbohydr. Res..

[cit73] Demus D. and Richter L., Textures of liquid crystals, Verlag Chemie, Weinheim New York, 1978

[cit74] Förster S., Apostol L., Bras W. (2010). J. Appl. Crystallogr..

[cit75] Praefcke K., Kohne B., Diele S., Pelzl G., Kjær A. (1992). Liq. Cryst..

[cit76] Singh M. K., Jayaraman N., Rao D. S. S., Prasad S. K. (2010). Chem. Phys. Lipids.

[cit77] Luyten M. C., Alberda Van Ekenstein G. O. R., Ten Brinke G., Ruokolainen J., Ikkala O., Torkkeli M., Serimaa R. (1999). Macromolecules.

[cit78] Luo Y., Montarnal D., Kim S., Shi W., Barteau K. P., Pester C. W., Hustad P. D., Christianson M. D., Fredrickson G. H., Kramer E. J., Hawker C. J. (2015). Macromolecules.

[cit79] Borisch K., Diele S., Göring P., Tschierske C. (1996). Chem. Commun..

[cit80] Huang Z., Qi P., Liu Y., Chai C., Wang Y., Song A., Hao J. (2019). Phys. Chem. Chem. Phys..

[cit81] Holder C. F., Schaak R. E. (2019). ACS Nano.

[cit82] Chong T. T., Hashim R., Bryce R. A. (2006). J. Phys. Chem. B.

[cit83] Manna M., Róg T., Vattulainen I. (2014). Biochim. Biophys. Acta, Mol. Cell Biol. Lipids.

