# Supplementary material for: Stereochemistry-dependent thermotropic liquid crystalline phases of monosaccharide-based amphiphiles
Source: Soft Matter. 2023 Oct 24;19(43):8360–77. doi: 10.1039/d3sm00939d (PMC10630951; doi:10.1039/d3sm00939d)
Supplement: SM-019-D3SM00939D-s001 [file SM-019-D3SM00939D-s001.pdf]

Electronic Supplementary Material (ESI) for Soft Matter.
This journal is © The Royal Society of Chemistry 2023

Supporting information

Stereochemistry-dependent thermotropic liquid crystalline phases of monosaccharide-based amphiphiles

Ida Mattsson,[†] Johanna Majoinen,[‡] Manu Lahtinen,[⊥] Thomas Sandberg,[†] Anna Fogde,[†] Tiina Saloranta-Simell,[†] Orlando J. Rojas,[‡,§] Olli Ikkala,[#] and Reko Leino*,[†]

[†] Laboratory of Molecular Science and Engineering, Johan Gadolin Process Chemistry Centre, Åbo Akademi University, FI-20500 Finland

[‡] Department of Bioproducts and Biosystems, School of Chemical Engineering, Aalto University, FI-00076 Aalto, Finland

[⊥] Department of Chemistry, University of Jyväskylä, FI-40014, Finland

[§] Bioproducts Institute, Department of Chemical and Biological Engineering, Department of Chemistry and Department of Wood Science, University of British Columbia, 2360 East Mall, Vancouver, BC V6T 1Z4, Canada

[#] Department of Applied Physics, Aalto University, Espoo FI-00076, Finland

Contents

Synthesis and characterization of allylated monosaccharides 4
Allylation of D-mannose (**Man**) to yield *t*-**Man*** 4
Allylation of D-glucose (**Glc**) to yield *t*-**Glc*** and *e*-**Glc*** 5
Allylation of D-galactose (**Gal**) to yield *t*-**Gal*** and *e*-**Gal*** 6
Allylation of L-arabinose (**Ara**) to yield *t*-**Ara*** and *e*-**Ara***........ 7
Allylation of L-rhamnose (**Rha**) to yield *t*-**Rha*** 8
Synthesis and characterization of liquid crystalline amphiphiles 9
Coupling of *t*-**Man*** with 1-hexanethiol to yield *t*-**Man*-SC6** 9
Coupling of *t*-**Man*** with 1-decanethiol to yield *t*-**Man*-SC10** 9
Coupling of *t*-**Man*** with 1-tetradecanethiol to yield *t*-**Man*-SC14** 10
Coupling of *t*-**Glc*** with 1-tetradecanethiol to yield *t*-**Glc*-SC14** 10
Coupling of *e*-**Glc*** with 1-tetradecanethiol to yield *e*-**Glc*-SC14** 11
Coupling of *t*-**Gal*** with 1-tetradecanethiol to yield *t*-**Gal*-SC14** 11
Coupling of *e*-**Gal*** with 1-tetradecanethiol to yield *e*-**Gal*-SC14** 12
Coupling of *t*-**Ara*** with 1-tetradecanethiol to yield *t*-**Ara*-SC14** 12
Coupling of *e*-**Ara*** with 1-tetradecanethiol to yield *e*-**Ara*-SC14** 13
Coupling of *t*-**Rha*** with 1-tetradecanethiol to yield *t*-**Rha*-SC14** 13
NMR spectra 14
^{1}H and ^{13}C NMR spectra of compound *t*-**Man*** 14
^{1}H-and ^{13}C NMR spectra of compound *t*-**Glc*** 15
^{1}H-and ^{13}C NMR spectra of compound *e*-**Glc*** 16
^{1}H-and ^{13}C NMR spectra of compound *t*-**Gal*** 17
^{1}H-and ^{13}C NMR spectra of compound *e*-**Gal*** 18
^{1}H-and ^{13}C NMR spectra of compound *t*-**Ara*** 19
^{1}H-and ^{13}C NMR spectra of compound *e*-**Ara*** 20
^{1}H-and ^{13}C NMR spectra of compound *t*-**Rha*** 21
^{1}H-and ^{13}C NMR spectra of compound *t*-**Man*-SC6** 22
^{1}H-and ^{13}C NMR spectra of compound *t*-**Man*-SC10** 23
^{1}H-and ^{13}C NMR spectra of compound *t*-**Man*-SC14** 24
^{1}H-and ^{13}C NMR spectra of compound *t*-**Glc*-SC14** 25
^{1}H-and ^{13}C NMR spectra of compound *e*-**Glc*-SC14** 26
^{1}H-and ^{13}C NMR spectra of compound *t*-**Gal*-SC14** 27
^{1}H-and ^{13}C NMR spectra of compound *e*-**Gal*-SC14** 28
^{1}H-and ^{13}C NMR spectra of compound *t*-**Ara*-SC14** 29
^{1}H-and ^{13}C NMR spectra of compound *e*-**Ara*-SC14** 30
^{1}H-and ^{13}C NMR spectra of compound *t*-**Rha*-SC14** 31
Conformational analysis of allylated monosaccharides 32
Thermal analysis of allylated monosaccharides 34
Thermal events for amphiphiles derived from allylated monosaccharides 35

DSC curves for amphiphiles derived from allylated monosaccharides 39
DSC curve for *t*-**Man*-SC6** 39
DSC curve for *t*-**Man*-SC10** 39
DSC curve for *t*-**Man*-SC14** 40
DSC curve for *t*-**Glc*-SC14** 40
DSC curve for *e*-**Glc*-SC14** 41
DSC curve for *t*-**Gal*-SC14** 41
DSC curve for *e*-**Gal*-SC14** 42
DSC curve for *t*-**Ara*-SC14** 42
DSC curve for *e*-**Ara*-SC14** 43
DSC curve for *t*-**Rha*-SC14** 43
Polarized Optical Microscopy Observations 44
References 51

Synthesis and characterization of allylated monosaccharides

Allylation of D-mannose (**Man**) to yield *t*-**Man***

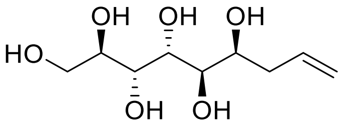

t*-Man

The synthetic procedure was conducted according to previously published methods[1-5]:

D-Mannose (5.0 g, 27.8 mmol, 1 eq.), tin powder (6.7 g, 56.4 mmol, 2 eq.) and allyl bromide (10.0 g, 83.2 mmol, 3 eq.) were dissolved in 550 ml EtOH and 50 ml distilled H_2O. The reaction mixture was heated to 60 °C and stirred overnight. The mixture was allowed to cool to room temperature and neutralized with 5 M NaOH (aq). The mixture was filtered through Celite. The filtrate was evaporated until approximately 80 ml solution remained. The solution was left in the refridgerator overnight to yield 2.96 g of *t*-**Man*** as a white powder (48% yield).

(2*R*,3*R*,4*R*,5*R*,6*S*)-Non-8-ene-1,2,3,4,5,6-hexaol (*t*-Man*)

^{1}H-NMR (500.20 MHz, D_2O, 25 °C): δ = 5.90 (dddd, $J_{8,7b}$ = 6.9 Hz, $J_{8,7a}$ = 7.1 Hz, $J_{8,9cis}$ = 10.2 Hz, $J_{8,9trans}$ = 17.2 Hz, 1 H, H-8), 5.18 (dddd, $J_{9trans,7a}$ = −1.4 Hz, $J_{9trans,7b}$ = −1.5 Hz, $J_{9trans,9cis}$ = −2.1 Hz, 1 H, H-9_{trans}), 5.13 (dddd, $J_{9cis,7a}$ = −1.1 Hz, $J_{9cis,7b}$ = −1.1 Hz, 1 H, H-9_{cis}), 3.99 (ddd, $J_{6,5}$ = 1.5 Hz, $J_{6,7b}$ = 5.7 Hz, $J_{6,7a}$ = 8.3 Hz, 1 H, H-6), 3.91 (dd, $J_{4,3}$ = 1.1 Hz, $J_{4,5}$ = 9.4 Hz, 1 H, H-4), 3.87 (dd, $J_{1a,2}$ = 3.0 Hz, $J_{1a,1b}$ = −11.9 Hz, 1 H, H-1a), 3.81 (dd, $J_{3,2}$ = 8.9 Hz, 1 H, H-3), 3.76 (ddd, $J_{2,1b}$ = 6.5 Hz, 1 H, H-2), 3.67 (dd, 1 H, H-1b), 3.59 (dd, 1 H, H-5), 2.40 (ddddd, $J_{7a,7b}$ = −14.2 Hz, 1 H, H-7a), 2.35 (ddddd, 1 H, H-7b) ppm. ^{13}C NMR (125.8 MHz, D_2O, 25 °C): δ = 135.2 (C-8), 117.3 (C-9), 71.0 (C-5), 70.9 (C-2), 69.4 (C-3), 69.3 (C-6), 68.5 (C-4), 63.2 (C-1), 37.6 (C-7) ppm.

Allylation of D-glucose (**Glc**) to yield *t*-**Glc*** and *e*-**Glc***

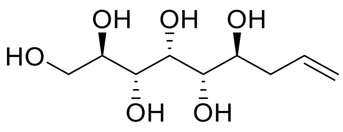

e*-Glc

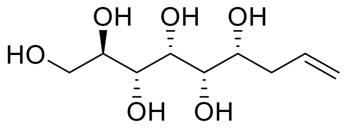

t*-Glc

The synthetic procedure was conducted according to previously published methods[1–3]:

D-Glucose (5.0 g, 27.8 mmol, 1 eq.), tin powder (6.7 g, 56.4 mmol, 2 eq.) and allyl bromide (10.0 g, 83.2 mmol, 3 eq.) were dissolved in 550 ml EtOH and 50 ml distilled H_2O. The reaction mixture was heated to 60 °C and stirred overnight. The mixture was allowed to cool to room temperature and neutralized with 5 M NaOH (aq). The mixture was filtered through Celite. The filtrate was evaporated to dryness and the crude product was obtained as an offwhite powder. The crude product was acetylated with acetic anhydride (9 eq.) in pyridine. The acetylated diastereomers were separated by column chromatography (hexane:ethyl acetate 2:1). Deacetylation was subsquently conducted with NaOMe in dry methanol. The reaction was neutralized with Dowex. The reaction mixtures were filtered and evaporated to dryness. The products were obtained as white powders: 3.24 g of *t*-**Glc*** and 0.86 g of *e*-**Glc*** equaling total yield of 4.10 g (66 %).

(2*R*,3*R*,4*R*,5*S*,6*R*)-Non-8-ene-1,2,3,4,5,6-hexaol (*t*-Glc*)

^{1}H NMR (500.20 MHz, D_2O, 25 °C): δ = 5.88 (dddd, $J_{8,7a}$ = 6.6 Hz, $J_{8,7b}$ = 7.4 Hz, $J_{8,9cis}$ = 10.2 Hz, $J_{8,9trans}$ = 17.2 Hz, 1 H, H-8), 5.19 (dddd, $J_{9trans,7a}$ = 1.5 Hz, $J_{9trans,7b}$ = 1.3 Hz, $J_{9trans,9cis}$ = 2.0 Hz, 1 H, H-9trans), 5.14 (dddd, $J_{9cis,7a}$ = 1.0 Hz, $J_{9cis,7b}$ = <1 Hz, 1 H, H-9cis), 3.96 (dd, $J_{4,3}$ = 2.3 Hz, $J_{4,5}$ = 6.1 Hz, 1 H, H-4), 3.84 (ddd, $J_{6,5}$ = 3.5 Hz, $J_{6,7a}$ = 5.0 Hz, $J_{6,7b}$ = 8.2 Hz, 1 H, H-6), 3.83 (dd, $J_{1a,1b}$ = -11.9 Hz, $J_{1a,2}$ = 3.1 Hz, 1 H, H-1a), 3.78 (ddd, $J_{2,1b}$ = 6.4 Hz, $J_{2,3}$ = 8.2 Hz, 1 H, H-2), 3.71 (dd, 1 H, H-3), 3.67 (dd, 1 H, H-5), 3.65 (dd, 1 H, H-1b), 2.39 (ddddd, $J_{7a,7b}$ = -14.3 Hz, 1 H, H-7a), 2.34 (m, 1 H, H-7b). ^{13}C NMR (125.8 MHz, D_2O, 25 °C): δ = 134.8 (C-8), 117.6 (C-9), 73.8 (C-5), 71.0 (C-3), 71.0 (C-2), 70.3 (C-6), 69.9 (C-4), 62.8 (C-1), 37.4 (C-7) ppm.

(2*R*,3*R*,4*R*,5*S*,6*S*)-Non-8-ene-1,2,3,4,5,6-hexaol (*e*-Glc*)

^{1}H NMR (500.20 MHz, D_2O, 25 °C): δ = 5.92 (dddd, $J_{8,7a}$ = 6.3 Hz, $J_{8,7b}$ = 7.8 Hz, $J_{8,9cis}$ = 10.3 Hz, $J_{8,9trans}$ = 17.2 Hz, 1 H, H-8), 5.19 (dddd, $J_{9trans,7a}$ = 1.6 Hz, $J_{9trans,7b}$ = 1.2 Hz, $J_{9trans,9cis}$ = 2.1 Hz, 1 H, H-9trans), 5.16 (dddd, $J_{9cis,7a}$ = 1.1 Hz, $J_{9cis,7b}$ = <1 Hz, 1 H, H-9cis), 4.00 (dd, $J_{4,3}$ = 3.0 Hz, $J_{4,5}$ = 3.7 Hz, 1 H, H-4), 3.81 (dd, $J_{1a,1b}$ = -11.9 Hz, $J_{1a,2}$ = 3.0 Hz, 1 H, H-1a), 3.80 (ddd, $J_{6,5}$ = 6.9 Hz, $J_{6,7a}$ = 3.1 Hz, $J_{6,7b}$ = 8.9 Hz, 1 H, H-6), 3.78 (ddd, $J_{2,1b}$ = 6.7 Hz, $J_{2,3}$ = 7.8 Hz, 1 H, H-2), 3.70 (dd, 1 H, H-3), 3.67 (dd, 1 H, H-5), 3.65 (dd, 1 H, H-1b), 2.46 (ddddd, $J_{7a,7b}$ = -14.6, 1 H, H-7a), 2.24 (ddddd, 1 H, H-7b) ppm. ^{13}C NMR (125.8 MHz, D_2O, 25 °C): δ = 134.9 (C-8), 117.7 (C-9), 74.9 (C-5), 72.6 (C-3), 71.1 (C-2), 70.2 (C-6), 68.5 (C-4), 62.3 (C-1), 36.3 (C-7) ppm. HRMS calcd. for $C_9H_{18}O_6Na$ $[M+Na]^+$ 245.1001, found 245.1028.

Allylation of D-galactose (**Gal**) to yield *t*-**Gal*** and *e*-**Gal***

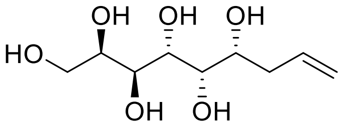

t-**Gal***

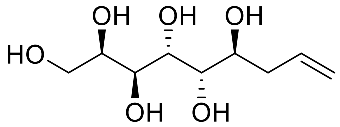

e-**Gal***

The synthetic procedure was conducted according to previously published methods[1–3]:

D-Galactose (5.0 g, 27.8 mmol, 1 eq.), tin powder (6.7 g, 56.4 mmol, 2 eq.) and allyl bromide (10.0 g, 83.2 mmol, 3 eq.) were dissolved in 550 ml EtOH and 50 ml distilled H_2O. The reaction mixture was heated to 60 °C and stirred overnight. The mixture was allowed to cool to room temperature and neutralized with 5 M NaOH (aq). The mixture was filtered through Celite. The filtrate was evaporated to dryness and the crude product was obtained as an offwhite powder. The crude product was acetylated with acetic anhydride (9 eq.) in pyridine. The acetylated diastereomers were separated by column chromatography (hexane:ethyl acetate 2:1). Deacetylation was subsequently conducted with NaOMe in dry methanol. The reaction was neutralized with Dowex. The reaction mixtures were filtered and evaporated to dryness. The products were obtained as white powders: 2.02 g of *t*-**Gal*** and 0.94 g of *e*-**Gal*** equaling total yield of 2.96 g (48 %).

(2*R*,3*S*,4*R*,5*S*,6*R*)-Non-8-ene-1,2,3,4,5,6-hexaol (*t*-Gal*)

^{1}H NMR (500.20 MHz, D_2O, 25 °C): δ = 5.90 (dddd, $J_{8,7a}$ = 6.5 Hz, $J_{8,7b}$ = 7.6 Hz, $J_{8,9cis}$ = 10.2 Hz, $J_{8,9trans}$ = 17.2 Hz, 1 H, H-8), 5.19 (dddd, $J_{9trans,7a}$ = 1.5 Hz, $J_{9trans,7b}$ = 1.3 Hz, $J_{9trans,9cis}$ = 2.0 Hz, 1 H, H-9trans), 5.15 (dddd, $J_{9cis,7a}$ = 1.1 Hz, $J_{9cis,7b}$ = <1 Hz, 1 H, H-9cis), 3.96 (ddd, $J_{2,1a}$ = 5.6 Hz, $J_{2,1b}$ = 7.2 Hz, $J_{2,3}$ = 1.5 Hz, 1 H, H-2), 3.86 (ddd, $J_{6,5}$ = 6.6 Hz, $J_{6,7a}$ = 4.1 Hz, $J_{6,7b}$ = 8.2 Hz, 1 H, H-6), 3.79 (dd, $J_{3,4}$ = 9.2 Hz, 1 H, H-3), 3.76 (dd, $J_{5,4}$ = 1.5 Hz, 1 H, H-5), 3.70 (dd, 1 H, H-4), 3.69 (dd, $J_{1a,1b}$ = -7.3, 1 H, H-1a), 3.69 (dd, 1 H, H1b), 2.44 (ddddd, $J_{7a,7b}$ = -14.5 Hz, 1 H, H-7a), 2.25 (ddddd, 1 H, H-7b) ppm. ^{13}C NMR (125.8 MHz, D_2O, 25 °C): δ = 134.5 (C-8), 117.8 (C-9), 72.2 (C-6), 71.7 (C-5), 70.0 (C-2), 70.0 (C-3), 69.5 (C-4), 63.2 (C-1), 37.0 (C-7) ppm.

(2*R*,3*S*,4*R*,5*S*,6*S*)-Non-8-ene-1,2,3,4,5,6-hexaol (*e*-Gal*)

^{1}H NMR (500.20 MHz, D_2O, 25 °C): δ = 5.94 (dddd, $J_{8,7a}$ = 6.4 Hz, $J_{8,7b}$ = 7.8 Hz, $J_{8,9cis}$ = 10.2 Hz, $J_{8,9trans}$ = 17.3 Hz, 1 H, H-8), 5.20 (dddd, $J_{9trans,7a}$ = 1.6 Hz, $J_{9trans,7b}$ = 1.2 Hz, $J_{9trans,9cis}$ = 2.1 Hz, 1 H, H-9trans), 5.16 (dddd, $J_{9cis,7a}$ = 1.1 Hz, $J_{9cis,7b}$ = <1 Hz, 1 H, H-9cis), 3.99 (ddd, $J_{2,1a}$ = 5.6 Hz, $J_{2,1b}$ = 7.2 Hz, $J_{2,3}$ = 1.5 Hz, 1 H, H-2), 3.94 (dd, $J_{4,3}$ = 9.4 Hz, $J_{4,5}$ = 1.1 Hz, 1 H, H-4), 3.79 (ddd, $J_{6,5}$ = 8.5 Hz, $J_{6,7a}$ = 3.2 Hz, $J_{6,7b}$ = 8.5 Hz, 1 H, H-6), 3.71 (dd, 1 H, H-5), 3.70 (dd, $J_{1a,1b}$ = -6.8 Hz, 1 H, H-1a), 3.69 (dd, 1 H, H-1b), 3.68 (dd, 1 H, H-3), 2.58 (ddddd, $J_{7a,7b}$ = -14.6 Hz, 1 H, H-7a), 2.25 (ddddd, 1 H, H-7b) ppm. ^{13}C NMR (125.8 MHz, D_2O, 25 °C): δ = 135.0 (C-8), 117.7 (C-9), 71.4 (C-5), 70.3 (C-2), 70.0 (C-6), 69.4 (C-3), 68.3 (C-4), 63.3 (C-1), 37.5 (C-7) ppm. HRMS calcd. for $C_9H_{18}O_6Na$ $[M+Na]^+$ 245.1001, found 245.1018.

Allylation of L-arabinose (**Ara**) to yield *t*-**Ara*** and *e*-**Ara***

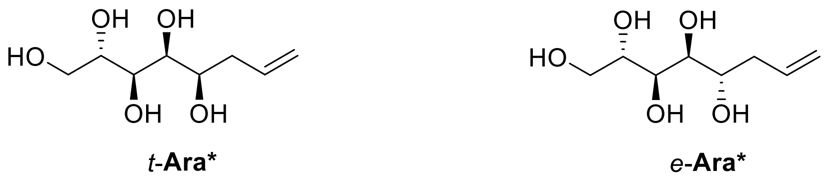

The synthetic procedure was conducted according to previously published methods[1,2]:

L-Arabinose (5.0 g, 33.3 mmol, 1 eq.), tin powder (7.9 g, 66.6 mmol, 2 eq.), and allyl bromide (12.1 g, 99.8 mmol, 3 eq.) were dissolved in 500 ml distilled water and 50 ml ethanol. The reaction mixture was heated to 60 °C and stirred at that temperature overnight. The reaction mixture was neutralized with 5 M NaOH solution and subsequently filtered through Celite. The filtrate was evaporated to dryness and the crude product was obtained as an offwhite powder. The crude product was acetylated with acetic anhydride (9 eq.) in pyridine. The acetylated diastereomers were separated by column chromatography (hexane:ethyl acetate 2:1). Deacetylation was subsequently conducted with NaOMe in dry methanol. The reaction was neutralized with Dowex. The reaction mixtures were filtered and evaporated to dryness. The products were obtained as white powdesr: 1.92 g of *t*-**Ara*** and 0.71 g of *e*-**Ara*** equaling total yield of 2.63 g (41 %).

(2*S*,3*S*,4*S*,5*R*)-Oct-7-ene-1,2,3,4,5-pentaol (*t*-Ara*)

^{1}H NMR (500.20 MHz, D_2O, 25 °C): δ = 5.89 (dddd, $J_{7,6a}$ = 6.5 Hz, $J_{7,6b}$ = 7.6 Hz, $J_{7,8cis}$ = 10.3 Hz, $J_{7,8trans}$ = 17.2 Hz, 1 H, H-7), 5.18 (dddd, $J_{8trans,6a}$ = 1.6 Hz, $J_{8trans,6b}$ = 1.3 Hz, $J_{8trans,8cis}$ = 2.0 Hz, 1 H, H-8trans), 5.15 (dddd, $J_{8cis,6a}$ = 1.2 Hz, $J_{8cis,6b}$ = <1 Hz, 1 H, H-8cis), 3.84 (ddd, $J_{5,4}$ = 6.2 Hz, $J_{5,6a}$ = 4.3 Hz, $J_{5,6b}$ = 8.2 Hz, 1 H, H-5), 3.83 (dd, $J_{1a,1b}$ = -11.9, $J_{1a,2}$ = 3.0, 1 H, H-1a), 3.77 (ddd, $J_{2,1b}$ = 6.5 Hz, $J_{2,3}$ = 8.3 Hz, 1 H, H-2), 3.72 (dd, $J_{4,3}$ = 2.1 Hz, 1 H, H-4), 3.68 (dd, 1 H, H-3), 3.64 (dd, 1 H, H-1b), 2.41 (ddddd, $J_{6a,6b}$ = -14.5 Hz, 1 H, H-6a), 2.25 (ddddd, 1 H, H-6b). ^{13}C NMR (125.8 MHz, D_2O, 25 °C): δ = 134.5 (C-7), 117.8 (C-8), 71.9 (C-5), 71.7 (C-4), 71.1 (C-3), 71.0 (C-2), 62.8 (C-1), 37.0 (C-6) ppm. HRMS calcd. for $C_8H_{16}O_5Na$ $[M+Na]^+$ 215.0895, found 215.0921.

(2*S*,3*S*,4*S*,5*S*)-Oct-7-ene-1,2,3,4,5-pentaol (*e*-Ara*)

^{1}H NMR (500.20 MHz, D_2O, 25 °C): δ = 5.93 (dddd, $J_{7,6a}$ = 6.4 Hz, $J_{7,6b}$ = 7.8 Hz, $J_{7,8cis}$ = 10.3 Hz, $J_{7,8trans}$ = 17.2 Hz, 1 H, H-7), 5.19 (dddd, $J_{8trans,6a}$ = 1.5 Hz, $J_{8trans,6b}$ = 1.2 Hz, $J_{8trans,8cis}$ = 2.1 Hz, 1 H, H-8trans), 5.16 (dddd, $J_{8cis,6a}$ = 1.1 Hz, $J_{8cis,6b}$ = <1 Hz, 1 H, H-8cis), 3.86 (dd, $J_{1a,1b}$ = -11.9, $J_{1a,2}$ = 3.0, 1 H, H-1a), 3.82 (dd, $J_{3,2}$ = 8.7 Hz, $J_{3,4}$ = 1.2 Hz, 1 H, H-3), 3.77 (ddd, $J_{5,4}$ = 8.4 Hz, $J_{5,6a}$ = 3.2 Hz, $J_{5,6b}$ = 8.4 Hz, 1 H, H-5), 3.75 (ddd, $J_{2,1b}$ = 6.5 Hz, 1 H, H-2), 3.68 (dd, 1 H, H-4), 3.66 (dd, 1 H, H-1b), 2.56 (ddddd, $J_{6a,6b}$ = -14.6 Hz, 1 H, H-6a), 2.25 (ddddd, 1 H, H-6b) ppm. ^{13}C NMR (125.8 MHz, D_2O, 25 °C): δ = 134.9 (C-7), 117.7 (C-8), 71.5 (C-4), 71.0 (C-2), 69.9 (C-5), 69.3 (C-3), 63.2 (C-1), 37.4 (C-6) ppm. HRMS calcd. for $C_8H_{16}O_5Na$ $[M+Na]^+$ 215.0895, found 215.0940.

Allylation of L-rhamnose (**Rha**) to yield *t*-**Rha***

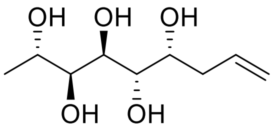

t-**Rha***

The synthetic procedure was conducted according to previously published methods[1,2,4]:

L-Rhamnose (5.0 g, 27.8 mmol, 1 eq.), tin powder (6.7 g, 56.4 mmol, 2 eq.) and allyl bromide (10.0 g, 83.2 mmol, 3 eq.) were dissolved in 550 ml EtOH and 50 ml distilled H_2O. The reaction mixture was heated to 60 °C and stirred overnight. The mixture was allowed to cool to room temperature and neutralized with 5 M NaOH (aq). The mixture was filtered through Celite. The filtrate was evaporated until approximately 80 ml solution remained. The solution was left in the refridgerator overnight to yield 2.47 g of *t*-**Rha*** as a white powder (43 % yield).

(2*S*,3*S*,4*R*,5*S*,6*R*)-Non-8-ene-2,3,4,5,6-pentaol (*t*-Rha*)

^{1}H-NMR (500.20 MHz, D_2O, 25 °C): δ = 5.90 (dddd, $J_{8,7a}$ = 7.1 Hz, $J_{8,7b}$ = 7.0 Hz, $J_{8,9cis}$ = 10.2 Hz, $J_{8,9trans}$ = 17.2 Hz, 1 H, H-8), 5.19 (dddd, $J_{9trans,7a}$ = 1.5 Hz, $J_{9trans,7b}$ = 1.5 Hz, $J_{9trans,9cis}$ = 2.1 Hz, 1 H, H-9trans), 5.14 (dddd, $J_{9cis,7a}$ = 1.1 Hz, $J_{9cis,7b}$ = 1.1 Hz, 1 H, H-9cis), 3.98 (ddd, $J_{6,5}$ = 1.6 Hz, $J_{6,7a}$ = 8.3 Hz, $J_{6,7b}$ = 5.8 Hz, 1 H, H-6), 3.91 (dd, $J_{4,3}$ = 1.3 Hz, $J_{4,5}$ = 9.2 Hz, 1 H, H-4), 3.88 (dq, $J_{2,1}$ = 6.3 Hz, $J_{2,3}$ = 7.9 Hz, 1 H, H-2), 3.62 (dd, 1 H, H-3), 3.59 (dd, 1 H, H-5), 2.40 (ddddd, $J_{7a,7b}$ = -14.2 Hz, 1H, H-7a), 2.35 (ddddd, 1H, H-7b), 1.28 (d, 3H, H-1) ppm. ^{13}C NMR (125.8 MHz, D_2O, 25 °C): δ = 135.2 (C-8), 117.4 (C-9), 73.4 (C-3), 71.2 (C-5), 69.3 (C-6), 68.5 (C-4), 67.2 (C-2), 37.6 (C-7), 19.0 (C-1) ppm.

Synthesis and characterization of liquid crystalline amphiphiles

Coupling of *t*-**Man*** with 1-hexanethiol to yield *t*-**Man*-SC6**

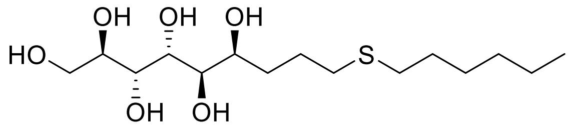

***t*-Man*-SC6**

Compound *t*-**Man*** (50 mg, 0.225 mmol, 1 eq.), 1-hexanethiol (53 mg, 0.45 mmol, 2 eq.) and 2,2-dimethoxy-2-phenylacetophenone (5.8 mg, 0.0225 mmol, 0.1 eq.) were dissolved in 5 ml DMF. The solution was irradiated with 365 nm 125 W UV-light for 1 h. The product was purified by washing the formed solids with 2 x 10 ml hexane and 5 ml distilled water, followed by centrifugation, decantation and freeze drying. Product *t*-**Man*-SC6** was obtained as a white powder (56.3 mg, 73 % yield).

(2*R*,3*R*,4*R*,5*R*,6*S*)-9-(Hexylthio)nonane-1,2,3,4,5,6-hexaol (*t*-Man*-SC6)

^{1}H NMR (500.20 MHz, DMSO, 25 °C): δ = 4.38 (d, 1 H, OH-2), 4.32 (t, 1 H, OH-1), 4.08 (d, 1 H, OH-4), 4.06 (d, 1 H, OH-5), 4.03 (d, 1 H, OH-3), 3.98 (d, 1 H, OH-6), 3.68 (m, 1 H, H-4), 3.67 (m, 1 H, H-6), 3.61 (m, 1 H, H-1a), 3.56 (m, 1 H, H-3), 3.47 (m, 1 H, H-2), 3.38 (m, 1 H, H-1b), 3.25 (m, 1 H, H-5), 2.49 (m, 2 H, H-9), 2.47 (m, 2 H, H-10), 1.63 (m, 1 H, H-8a), 1.53 (m, 1 H, H-7a), 1.53 (m, 1 H, H-8b), 1.50 (m, 2 H, H-11), 1.46 (m, 1 H, H-7b), 1.27 (m, 4 H, H-12, H-14), 1.25 (m, 2 H, H-13), 0.86 (t, 3 H, H-15) ppm. ^{13}C NMR (125.8 MHz, DMSO, 25 °C): δ = 71.5 (C-2), 71.4 (C-5), 69.7 (C-3), 69.0 (C-6), 68.8 (C-4), 63.9 (C-1), 32.9 (C-7), 31.4 (C-9), 31.1 (C-10), 30.8 (C-13), 29.1 (C-11), 27.9 (C-12), 26.1 (C-8), 22.0 (C-14), 13.9 (C-15) ppm. HRMS calcd. for $C_{15}H_{32}O_6SNa$ $[M+Na]^+$ 363.1817, found 363.1819.

Coupling of *t*-**Man*** with 1-decanethiol to yield *t*-**Man*-SC10**

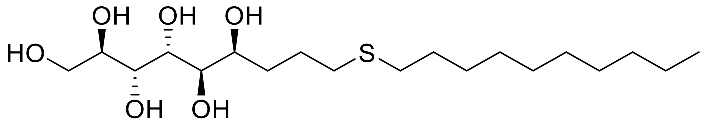

***t*-Man*-SC10**

Compound *t*-**Man*** (50 mg, 0.225 mmol, 1 eq.), 1-decanethiol (78 mg, 0.45 mmol, 2 eq.) and 2,2-dimethoxy-2-phenylacetophenone (5.8 mg, 0.0225 mmol, 0.1 eq.) were dissolved in 5 ml DMF. The solution was irradiated with 365 nm 125 W UV-light for 1 h. The product was purified by washing the formed solids with 2 x 10 ml hexane and 5 ml distilled water, followed by centrifugation, decantation and freeze drying. Product *t*-**Man*-SC10** was obtained as a white powder (64.2 mg, 72 % yield).

(2*R*,3*R*,4*R*,5*R*,6*S*)-9-(Decylthio)nonane-1,2,3,4,5,6-hexaol (*t*-Man*-SC10)

^{1}H NMR (500.20 MHz, DMSO, 25 °C): δ = 4.38 (d, 1 H, OH-2), 4.32 (t, 1 H, OH-1), 4.07 (d, 1 H, OH-4), 4.05 (d, 1 H, OH-5), 4.03 (d, 1 H, OH-3), 3.98 (d, 1 H, OH-6), 3.68 (m, 1 H, H-4), 3.67 (m, 1 H, H-6), 3.61 (m, 1 H, H-1a), 3.56 (m, 1 H, H-3), 3.48 (m, 1 H, H-2), 3.38 (m, 1 H, H-1b), 3.25 (m, 1 H, H-5), 2.48 (m, 2 H, H-9), 2.46 (m, 2 H, H-10), 1.63 (m, 1 H, H-8a), 1.53 (m, 1 H, H-7a), 1.53 (m, 1 H, H-8b), 1.50 (m, 2 H, H-11), 1.48 (m, 1 H, H-7b), 1.57-1.17 (m, 14 H, H-12-H-18), 0.86 (t, 3 H, H-19) ppm. ^{13}C NMR (125.8 MHz, DMSO, 25 °C): δ = 71.5 (C-2), 71.4 (C-5), 69.7 (C-3), 69.0 (C-6), 68.8 (C-4), 63.9 (C-1), 32.9 (C-7), 31.4 (C-9), 31.1 (C-10), 26.2 (C-8), 13.9 (C-19), 31.3, 29.1, 29.0, 28.9, 28.7, 28.6, 28.2, 22.0 (C-11 – C-18) ppm. HRMS calcd. for $C_{19}H_{40}O_6SNa$ $[M+Na]^+$ 419.2443 found 419.2455

Coupling of *t*-**Man*** with 1-tetradecanethiol to yield *t*-**Man*-SC14**

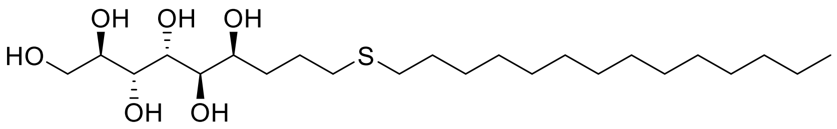

***t*-Man*-SC14**

Compound *t*-**Man*** (50 mg, 0.225 mmol, 1 eq.), 1-tetradecanethiol (104 mg, 0.45 mmol, 2 eq.) and 2,2-dimethoxy-2-phenylacetophenone (5.8 mg, 0.0225 mmol, 0.1 eq.) were dissolved in 5 ml DMF. The solution was irradiated with 365 nm 125 W UV-light for 1 h. The product was purified by washing the formed solids with 2 x 10 ml hexane and 5 ml distilled water, followed by centrifugation, decantation and freeze drying. Product *t*-**Man*-SC14** was obtained as a white powder (84.5 mg, 83 % yield).

(2*R*,3*R*,4*R*,5*R*,6*S*)-9-(Tetradecylthio)nonane-1,2,3,4,5,6-hexaol (*t*-Man*-SC14)

^{1}H NMR (500.20 MHz, DMSO, 25 °C): δ = 4.38 (d, 1 H, OH-2), 4.32 (t, 1 H, OH-1), 4.07 (d, 1 H, OH-4), 4.05 (d, 1 H, OH-5), 4.03 (d, 1 H, OH-3), 3.98 (d, 1 H, OH-6), 3.68 (m, 1 H, H-4), 3.67 (m, 1 H, H-6), 3.61 (m, 1 H, H-1a), 3.56 (m, 1 H, H-3), 3.47 (m, 1 H, H-2), 3.38 (m, 1 H, H-1b), 3.25 (m, 1 H, H-5), 2.48 (m, 2 H, H-9), 2.46 (m, 2 H, H-10), 1.63 (m, 1 H, H-8a), 1.53 (m, 1 H, H-7a), 1.53 (m, 1 H, H-8b), 1.50 (m, 2 H, H-11), 1.48 (m, 1 H, H-7b), 1.57-1.17 (m, 22 H, H-12-H-22), 0.86 (t, 3 H, H-23) ppm. ^{13}C NMR (125.8 MHz, DMSO, 25 °C): δ = 71.5 (C-2), 71.4 (C-5), 69.7 (C-3), 69.0 (C-6), 68.8 (C-4), 63.9 (C-1), 32.9 (C-7), 31.4 (C-9), 31.1 (C-10), 26.1 (C-8), 13.9 (C-23), 31.3, 29.2, 29.0, 28.7, 28.6, 28.3, 22.1 (C-11 – C-22) ppm. HRMS calcd. for $C_{19}H_{40}O_6SNa$ $[M+Na]^+$ 475.3064 found 475.3045

Coupling of *t*-**Glc*** with 1-tetradecanethiol to yield *t*-**Glc*-SC14**

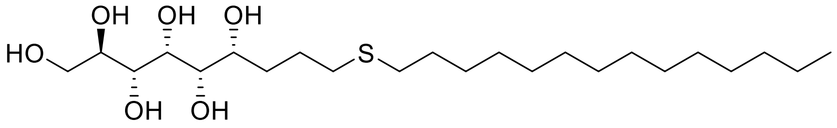

***t*-Glc*-SC14**

Compound *t*-**Glc*** (50 mg, 0.225 mmol, 1 eq.), 1-tetradecanethiol (104 mg, 0.45 mmol, 2 eq.) and 2,2-dimethoxy-2-phenylacetophenone (5.8 mg, 0.0225 mmol, 0.1 eq.) were dissolved in 5 ml DMF. The solution was irradiated with 365 nm 125 W UV-light for 1 h. The product was purified by washing the formed solids with 2 x 10 ml hexane and 5 ml distilled water, followed by centrifugation, decantation and freeze drying. Product *t*-**Glc*-SC14** was obtained as a white powder (75.6 mg, 76 % yield).

(2*R*,3*R*,4*R*,5*S*,6*R*)-9-(Tetradecylthio)nonane-1,2,3,4,5,6-hexaol (*t*-Glc*-SC14)

^{1}H NMR (500.20 MHz, DMSO, 25 °C): δ = 4.45 (d, 1 H, OH-3), 4.42 (d, 1 H, OH-2), 4.34 (d, 1 H, OH-6), 4.33 (d, 1 H, OH-5), 4.31 (t, 1 H, OH-1), 4.13 (d, 1 H, OH-4), 3.74 (m, 1 H, H-4), 3.57 (m, 1 H, H-1a), 3.51 (m, 1 H, H-6), 3.48 (m, 1 H, H-2), 3.43 (m, 1 H, H-3), 3.37 (m, 1 H, H-1b), 3.34 (m, 1 H, H-5), 2.47 (m, 2 H, H-9), 2.47 (m, 2 H, H-10), 1.63 (m, 1 H, H-8a), 1.49 (m, 1 H, H-8b), 1.49 (m, 2 H, H-11), 1.48 (m, 2 H, H-7), 1.37-1.18 (m, 22 H, H-12 – H-22), 0.85 (t, 3 H, H-23) ppm. ^{13}C NMR (125.8 MHz, DMSO, 25 °C): δ = 74.5 (C-5), 71.5 (C-3), 71.3 (C-2), 69.8 (C-6), 69.4 (C-4), 63.4 (C-1), 32.6 (C-7), 31.2 (C-9), 31.0 (C-10), 29.0 (C-11), 25.8 (C-8), 13.9 (C-23), 31.2, 28.9, 28.6, 28.5, 28.2, 22.0 (C-12 – C-22) ppm. HRMS calcd. for $C_{23}H_{48}O_6SNa$ $[M+Na]^+$ 475.3064, found 475.3073.

Coupling of *e*-**Glc*** with 1-tetradecanethiol to yield *e*-**Glc*-SC14**

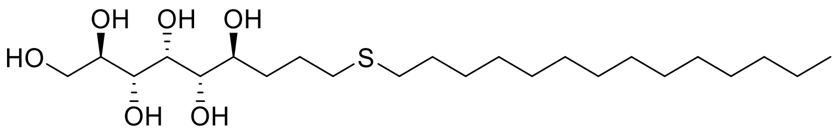

***e*-Glc*-SC14**

Compound *e*-**Glc*** (50 mg, 0.225 mmol, 1 eq.), 1-tetradecanethiol (104 mg, 0.45 mmol, 2 eq.) and 2,2-dimethoxy-2-phenylacetophenone (5.8 mg, 0.0225 mmol, 0.1 eq.) were dissolved in 5 ml DMF. The solution was irradiated with 365 nm 125 W UV-light for 1 h. The product was purified by washing the formed solids with 2 x 10 ml hexane and 5 ml distilled water, followed by centrifugation, decantation and freeze drying. Product *e*-**Glc*-SC14** was obtained as a white powder (83.4 mg, 85 % yield).

(2*R*,3*R*,4*R*,5*S*,6*S*)-9-(Tetradecylthio)nonane-1,2,3,4,5,6-hexaol (*e*-Glc*-SC14)

^{1}H NMR (500.20 MHz, DMSO, 25 °C): δ = 4.55 (d, 1 H, OH-3), 4.51 (d, 1 H, OH-5), 4.49 (d, 1 H, OH-2), 4.44 (d, 1 H, OH-6), 4.32 (t, 1 H, OH-1), 4.05 (d, 1 H, OH-4), 3.90 (m, 1 H, H-4), 3.56 (m, 1 H, H-1a), 3.50 (m, 1 H, H-2), 3.44 (m, 1 H, H-6), 3.40 (m, 1 H, H-3), 3.37 (m, 1 H, H-1b), 3.27 (m, 1 H, H-5), 2.47 (m, 2 H, H-9), 2.47 (m, 2 H, H-10), 1.69 (m, 1 H, H-8a), 1.68 (m, 1 H, H-7a), 1.52 (m, 1 H, H-8b), 1.50 (m, 2 H, H-11), 1.38-1.18 (m, 22 H, H-12 – H-22), 1.31 (m, 1 H, H-7b), 0.85 (t, 3 H, H-23) ppm. ^{13}C NMR (125.8 MHz, DMSO, 25 °C): δ = 76.4 (C-5), 74.0 (C-3), 71.3 (C-2), 69.8 (C-6), 67.3 (C-4), 63.2 (C-1), 32.2 (C-7), 31.4 (C-9), 31.0 (C-10), 29.0 (C-11), 25.4 (C-8), 13.8 (C-23), 31.2, 28.9, 28.6, 28.5, 28.2, 22.0 (C-12 – C-22) ppm. HRMS calcd. for $C_{23}H_{48}O_6SNa$ $[M+Na]^+$ 475.3064, found 475.3108.

Coupling of *t*-**Gal*** with 1-tetradecanethiol to yield *t*-**Gal*-SC14**

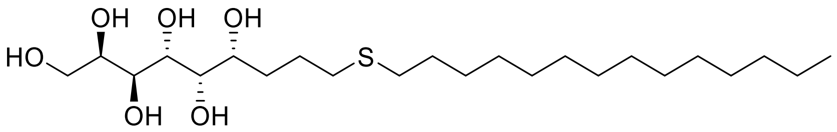

***t*-Gal*-SC14**

Compound *t*-**Gal*** (50 mg, 0.225 mmol, 1 eq.), 1-tetradecanethiol (104 mg, 0.45 mmol, 2 eq.) and 2,2-dimethoxy-2-phenylacetophenone (5.8 mg, 0.0225 mmol, 0.1 eq.) were dissolved in 5 ml DMF. The solution was irradiated with 365 nm 125 W UV-light for 1 h. The product was purified by washing the formed solids with 2 x 10 ml hexane and 5 ml distilled water, followed by centrifugation, decantation and freeze drying. Product *t*-**Gal*-SC14** was obtained as a white powder (93.5 mg, 94 % yield).

(2*R*,3*S*,4*R*,5*S*,6*R*)-9-(Tetradecylthio)nonane-1,2,3,4,5,6-hexaol (*t*-Gal*-SC14)

^{1}H NMR (500.20 MHz, DMSO, 25 °C): δ = 4.41 (t, 1 H, OH-1), 4.38 (d, 1 H, OH-6), 4.22 (d, 1 H, OH-4), 4.11 (d, 1 H, OH-2), 4.05 (d, 1 H, OH-5), 4.04 (d, 1 H, OH-3), 3.69 (m, 1 H, H-2), 3.51 (m, 1 H, H-6), 3.48 (m, 1 H, H-3), 3.47 (m, 1 H, H-4), 3.47 (m, 1 H, H-5), 3.39 (m, 2 H, H-1), 2.48 (m, 2 H, H-9), 2.47 (m, 2 H, H-10), 1.66 (m, 1 H, H-8a), 1.52 (m, 1 H, H-8b), 1.52 (m, 1 H, H-7a), 1.49 (m, 2 H, H-11), 1.38 (m, 1 H, H-7b), 1.36-1.19 (m, 22 H, H-12 – H-22), 0.85 (t, 3 H, H-23) ppm. ^{13}C NMR (125.8 MHz, DMSO, 25 °C): δ = 72.0 (C-6), 71.5 (C-3), 70.6 (C-4), 69.7 (C-2), 69.2 (C-5), 63.0 (C-1), 32.2 (C-7), 31.3 (C-9), 31.0 (C-10), 29.1 (C-11), 25.6 (C-8), 13.9 (C-23), 31.2, 28.9, 28.6, 28.5, 28.1, 22.0 (C-12 – C-22) ppm. HRMS calcd. for $C_{23}H_{48}O_6SNa$ $[M+Na]^+$ 475.3064, found 475.3076.

Coupling of *e*-**Gal*** with 1-tetradecanethiol to yield *e*-**Gal*-SC14**

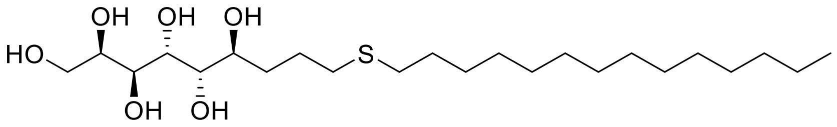

***e*-Gal*-SC14**

Compound *e*-**Gal*** (50 mg, 0.225 mmol, 1 eq.), 1-tetradecanethiol (104 mg, 0.45 mmol, 2 eq.) and 2,2-dimethoxy-2-phenylacetophenone (5.8 mg, 0.0225 mmol, 0.1 eq.) were dissolved in 5 ml DMF. The solution was irradiated with 365 nm 125 W UV-light for 1 h. The product was purified by washing the formed solids with 2 x 10 ml hexane and 5 ml distilled water, followed by centrifugation, decantation and freeze drying. Product *e*-**Gal*-SC14** was obtained as a white powder (92.6 mg, 94 % yield).

(2*R*,3*S*,4*R*,5*S*,6*S*)-9-(Tetradecylthio)nonane-1,2,3,4,5,6-hexaol (*e*-Gal*-SC14)

^{1}H NMR (500.20 MHz, DMSO, 25 °C): δ = 4.41 (t, 1 H, OH-1), 4.38 (d, 1 H, OH-6), 4.10 (d, 1 H, OH-2), 4.00 (d, 1 H, OH-5), 3.96 (d, 1 H, OH-4), 3.95 (d, 1 H, OH-3), 3.72 (m, 1 H, H-4), 3.71 (m, 1 H, H-2), 3.44 (m, 1 H, H-3), 3.41 (m, 1 H, H-6), 3.40 (m, 2 H, H-1), 3.38 (m, 1 H, H-5), 2.47 (m, 4 H, H-9, H-10), 1.74 (m, 1 H, H-7a), 1.71 (m, 1 H, H-8a), 1.52 (m, 1 H, H-8b), 1.50 (m, 2 H, H-11), 1.38-1.17 (m, 22 H, H-12 – H-22), 1.32 (m, 1 H, H-7b), 0.85 (t, 3 H, H-23) ppm. ^{13}C NMR (125.8 MHz, DMSO, 25 °C): δ = 71.8 (C-5), 70.1 (C-2), 70.0 (C-6), 69.2 (C-3), 68.3 (C-4), 63.1 (C-1), 33.1 (C-7), 31.5 (C-9), 31.2 (C-10), 29.1 (C-11), 25.5 (C-8), 13.9 (C-23), 31.0, 28.9, 286, 28. 28.5, 28.1, 22.0 (C-12 – C-22) ppm. HRMS calcd. for $C_{23}H_{48}O_6SNa$ $[M+Na]^+$ 475.3064, found 475.3073.

Coupling of *t*-**Ara*** with 1-tetradecanethiol to yield *t*-**Ara*-SC14**

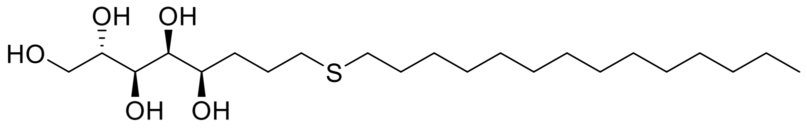

***t*-Ara*-SC14**

Compound *t*-**Ara*** (50 mg, 0.26 mmol, 1 eq.), 1-tetradecanethiol (119 mg, 0.52 mmol, 2 eq.) and 2,2-dimethoxy-2-phenylacetophenone (6.7 mg, 0.026 mmol, 0.1 eq.) were dissolved in 5 ml DMF. The solution was irradiated with 365 nm 125 W UV-light for 1 h. The product was purified by washing the formed solids with 2 x 10 ml hexane and 5 ml distilled water, followed by centrifugation, decantation and freeze drying. Product *t*-**Ara*-SC14** was obtained as white powder (85.9 mg, 81 % yield).

(2*S*,3*S*,4*S*,5*R*)-8-(Tetradecylthio)octane-1,2,3,4,5-pentaol (*t*-Ara*-SC14)

^{1}H NMR (500.20 MHz, DMSO, 25 °C): δ = 4.45 (d, 1 H, OH-2), 4.39 (d, 1 H, OH-5), 4.30 (t, 1 H, OH-1), 4.30 (d, 1 H, OH-3), 4.12 (d, 1 H, OH-4), 3.57 (m, 1 H, H-1a), 3.53-3.42 (m, 3 H, H-2, H-4, H-5), 3.37 (m, 1 H, H-1b), 3.34 (m, 1 H, H-3), 2.46 (m, 2 H, H-8), 2.46 (m, 2 H, H-9), 1.65 (m, 1 H, H-7a), 1.51 (m, 1 H, H-7b), 1.51 (m, 1 H, H-6a), 1.49 (m, 2 H, H-10), 1.38 (m, 1 H, H-6b), 1.37-1.19 (m, 22 H, H-11 – H-21), 0.85 (t, 3 H, H-22) ppm. ^{13}C NMR (125.8 MHz, DMSO, 25 °C): δ = 71.8 (C-3), 71.8 (C-5), 71.4 (C-4), 71.3 (C-2), 63.3 (C-1), 32.1 (C-6), 31.3 (C-8), 31.0 (C-9), 29.0 (C-10), 25.6 (C-7), 13.9 (C-23), 31.2, 28.9, 28.6, 28.5, 28.1, 22.0 (C-11 – C-21) ppm. HRMS calcd. for $C_{22}H_{46}O_5SNa$ $[M+Na]^+$ 445.2958, found 445.2969.

Coupling of *e*-**Ara*** with 1-tetradecanethiol to yield *e*-**Ara*-SC14**

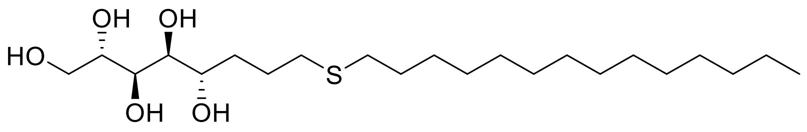

***e*-Ara*-SC14**

Compound *e*-**Ara*** (50 mg, 0.26 mmol, 1 eq.), 1-tetradecanethiol (119 mg, 0.52 mmol, 2 eq.) and 2,2-dimethoxy-2-phenylacetophenone (6.7 mg, 0.026 mmol, 0.1 eq.) were dissolved in 5 ml DMF. The solution was irradiated with 365 nm 125 W UV-light for 1 h. The product was purified by washing the formed solids with 2 x 10 ml hexane and 5 ml distilled water, followed by centrifugation, decantation and freeze drying. Product *e*-**Ara*-SC14** was obtained as a white powder (89.4 mg, 84 % yield).

(2*S*,3*S*,4*S*,5*S*)-8-(Tetradecylthio)octane-1,2,3,4,5-pentaol (*e*-Ara*-SC14)

^{1}H NMR (500.20 MHz, DMSO, 25 °C): δ = 4.41 (d, 1 H, OH-2), 4.40 (d, 1 H, OH-5), 4.32 (t, 1 H, OH-1), 4.10 (d, 1 H, OH-4), 4.03 (d, 1 H, OH-3), 3.60 (m, 1 H, H-1a), 3.57 (m, 1 H, H-3), 3.45 (m, 1 H, H-2), 3.40 (m, 1 H, H-5), 3.37 (m, 1 H, H-1b), 3.35 (m, 1 H, H-4), 2.47 (m, 2 H, H-8), 2.47 (m, 2 H, H-9), 1.73 (m, 1 H, H-6a), 1.71 (m, 1 H, H-7a), 1.52 (m, 1 H, H-7b), 1.50 (m, 2 H, H-10), 1.37-1.19 (m, 22 H, H-11 – H-21), 1.31 (m, 1 H, H-6b), 0.85 (t, 3 H, H-22) ppm. ^{13}C NMR (125.8 MHz, DMSO, 25 °C): δ = 71.9 (C-4), 71.4 (C-2), 69.7 (C-5), 69.6 (C-3), 63.8 (C-1), 33.0 (C-6), 31.5 (C-8), 31.0 (C-9), 29.1 (C-10), 25.5 (C-7), 13.9 (C-23), 31.2, 28.9, 28.6, 28.5, 28.1, 22.0 (C-11 – C-21) ppm. HRMS calcd. for $C_{22}H_{46}O_5SNa$ $[M+Na]^+$ 445.2958, found 445.2983.

Coupling of *t*-**Rha*** with 1-tetradecanethiol to yield *t*-**Rha*-SC14**

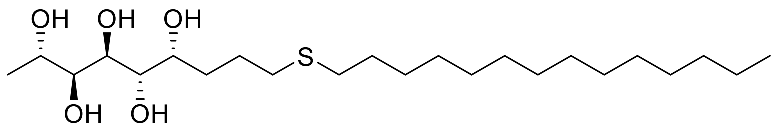

***t*-Rha*-SC14**

Compound *t*-**Rha*** (100 mg, 0.48 mmol, 1 eq.), 1-tetradecanethiol (221.2 mg, 0.96 mmol, 2 eq.) and 2,2-dimethoxy-2-phenylacetophenone (12.3 mg, 0.048 mmol, 0.1 eq.) were dissolved in 10 ml DMF. The solution was irradiated with 365 nm 125 W UV-light for 1 h. The product was purified by washing the formed solids with 2 x 10 ml hexane and 5 ml distilled water, followed by centrifugation, decantation and freeze drying. Product *t*-**Rha*-SC14** was obtained as a white powder (179.9 mg, 89 % yield).

(2*S*,3*S*,4*R*,5*S*,6*R*)-9-(Tetradecylthio)nonane-2,3,4,5,6-pentaol (*t*-Rha*-SC14)

^{1}H NMR (500.20 MHz, DMSO, 25 °C): δ = 4.38 (d, 1 H, OH-2), 4.08 (d, 1 H, OH-5), 4.00 (d, 1 H, OH-4), 3.99 (d, 1 H, OH-3), 3.98 (d, 1 H, OH-6), 3.70 (m, 1 H, H-4), 3.66 (m, 1 H, H-6), 3.58 (m, 1 H, H-2), 3.32 (m, 1 H, H-3), 3.24 (m, 1 H, H-5), 2.48 (m, 2 H, H-9), 2.47 (m, 2 H, H-10), 1.63 (m, 1 H, H-8a), 1.53 (m, 1 H, H-7a), 1.52 (m, 1 H, H-8b), 1.50 (m, 2 H, H-11), 1.46 (m, 1 H, H-7b), 1.33 (m, 2 H, H-12), 1.30-1.19 (m, 20 H, H-13 - H-22), 1.11 (t, 3 H, H-1), 0.85 (t, 3 H, H-23) ppm. ^{13}C NMR (125.8 MHz, DMSO, 25 °C): δ = 73.4 (C-3), 71.6 (C-5), 69.0 (C-6), 68.5 (C-4), 66.5 (C-2), 32.8 (C-7), 31.3 (C-9), 31.0 (C-10), 29.1 (C-11), 28.2 (C-12), 26.0 (C-8), 20.8 (C-1), 13.8 (C-23), 31.2, 28.9, 28.6, 28.5, 22.0 (C-13 – C-22). HRMS calcd. for $C_{23}H_{48}O_5SNa$ $[M+Na]^+$ 459.3115, found 459.3115.

NMR spectra

^{1}H and ^{13}C NMR spectra of compound *t*-**Man***

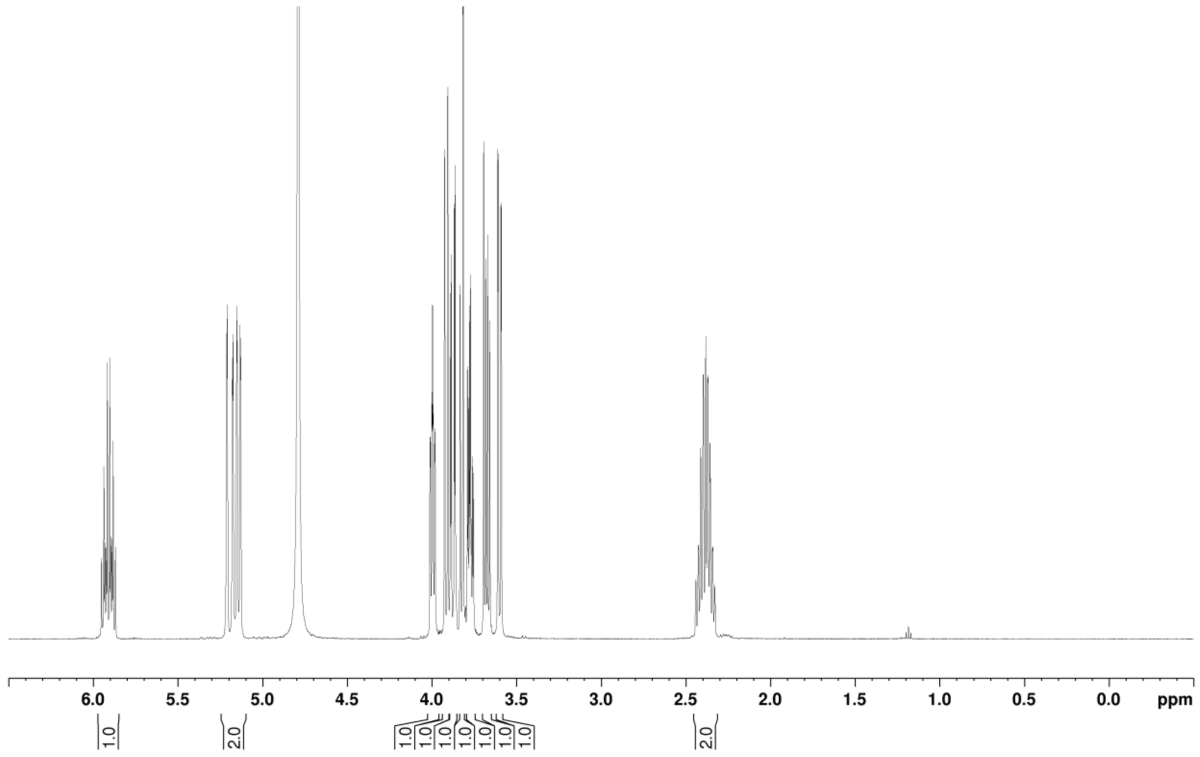

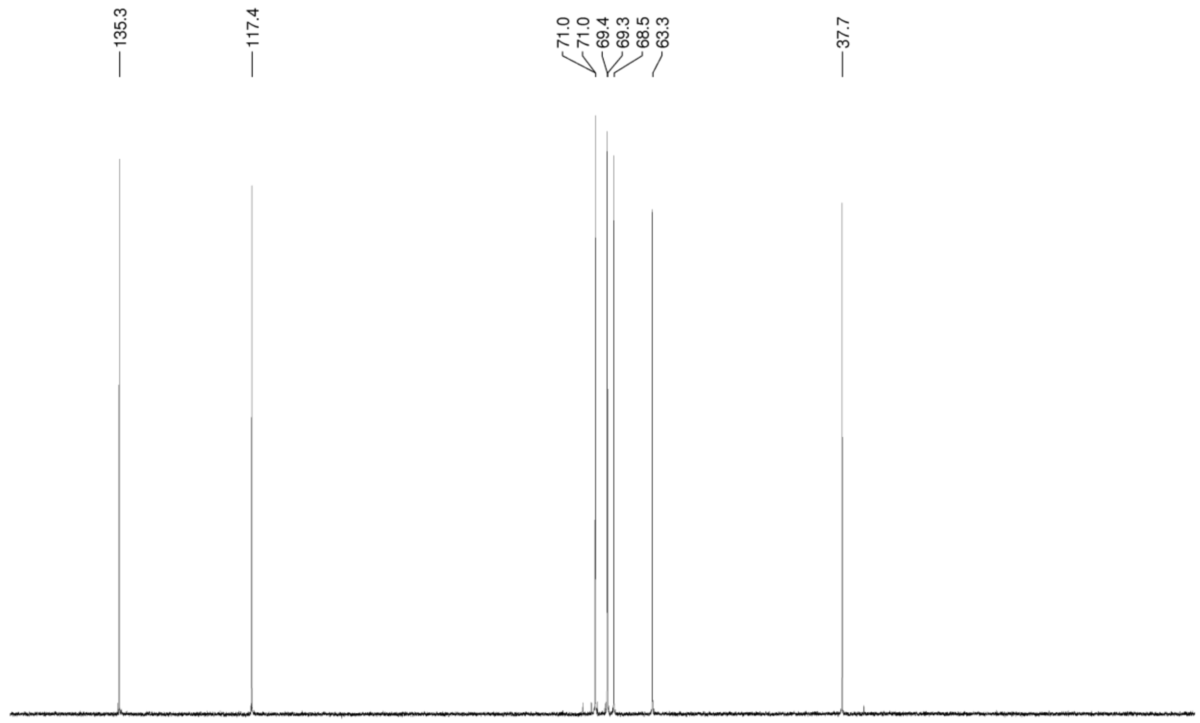

^{1}H-and ^{13}C NMR spectra of compound *t*-**Glc***

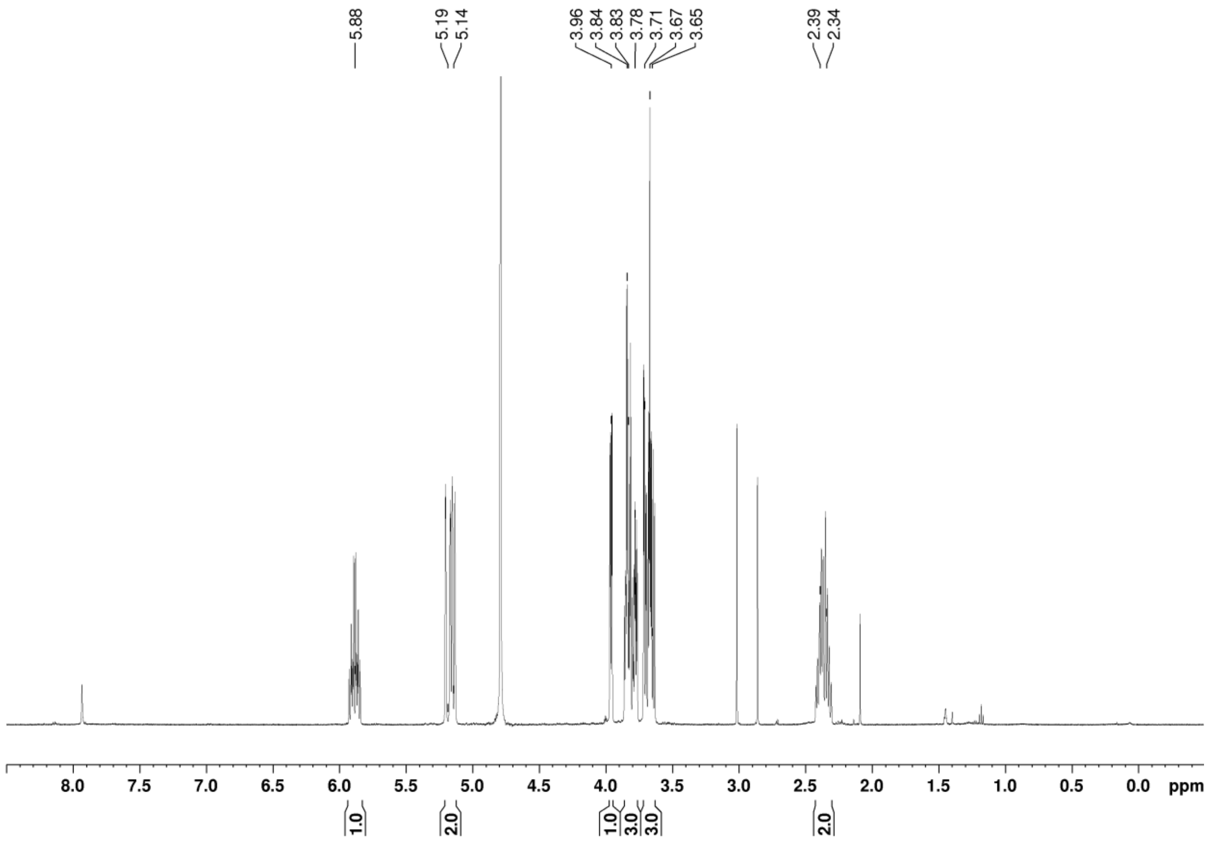

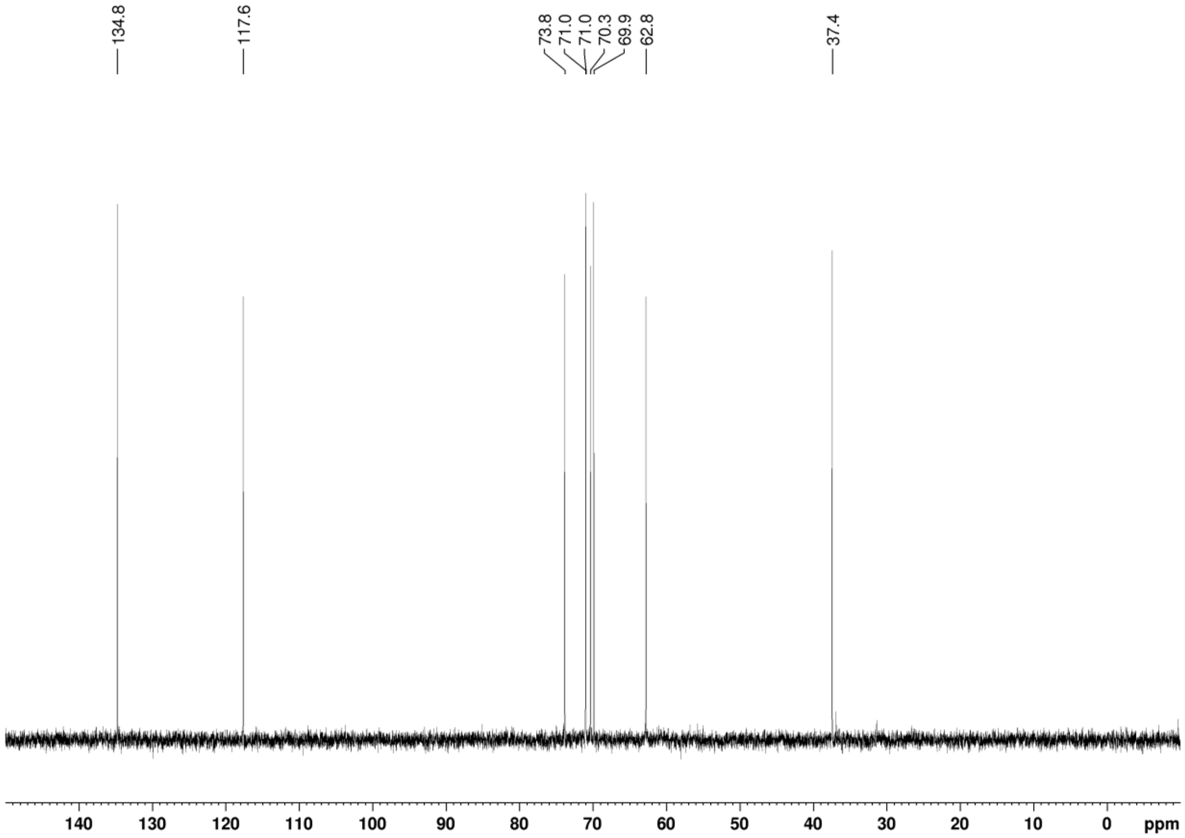

^{1}H-and ^{13}C NMR spectra of compound *e*-**Glc***

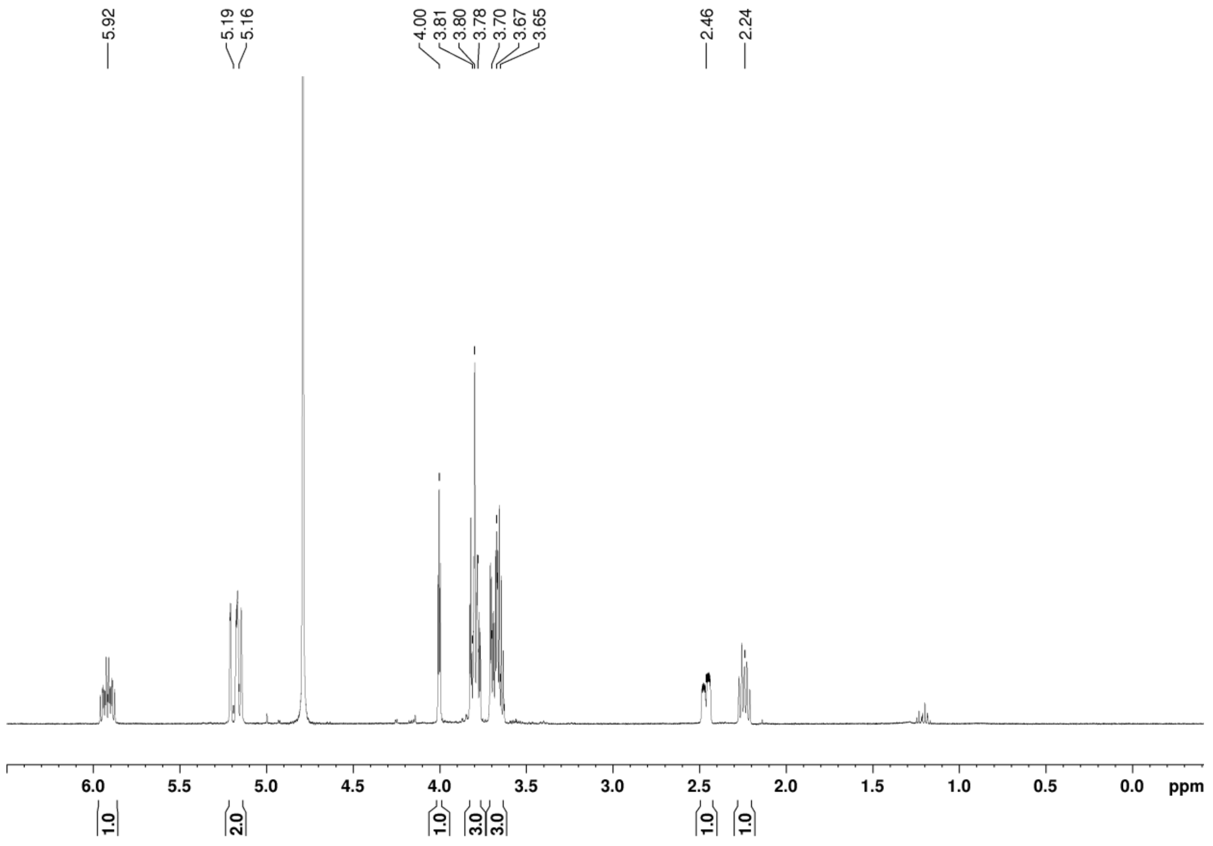

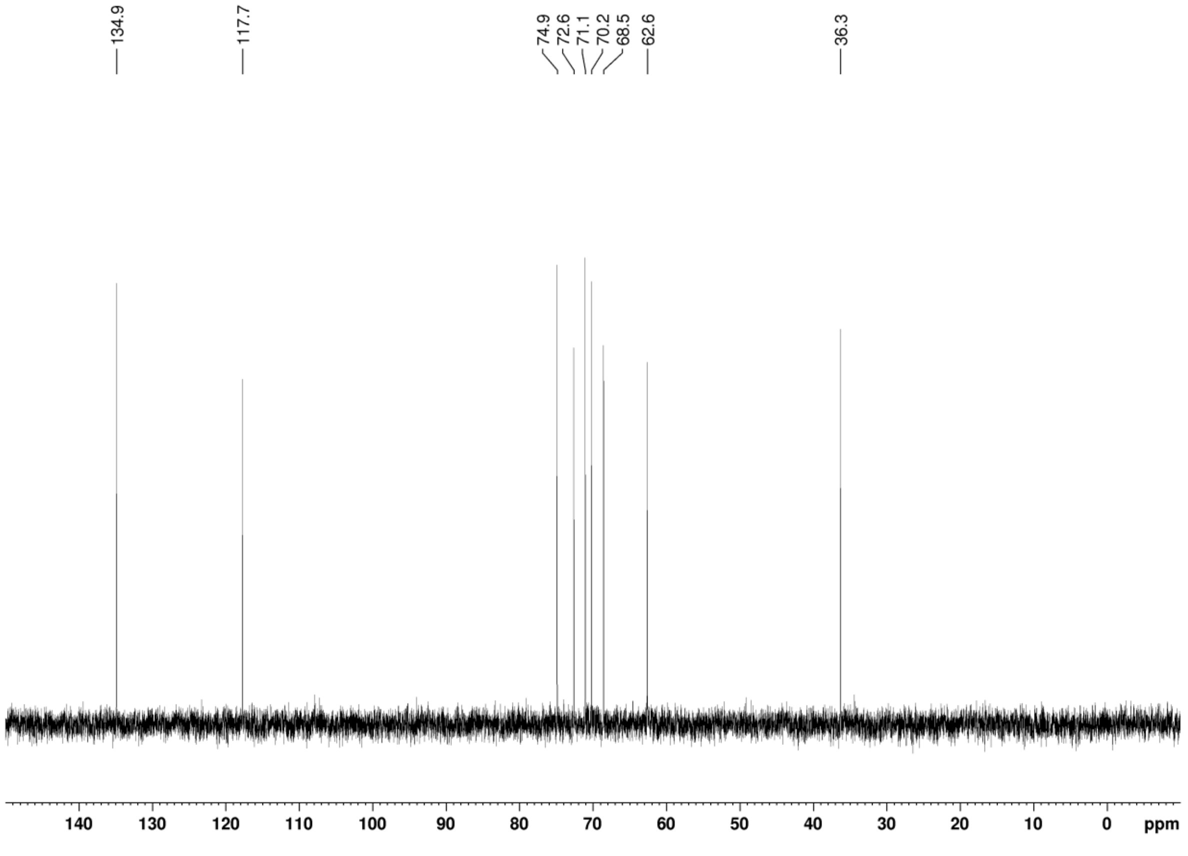

^{1}H-and ^{13}C NMR spectra of compound *t*-**Gal***

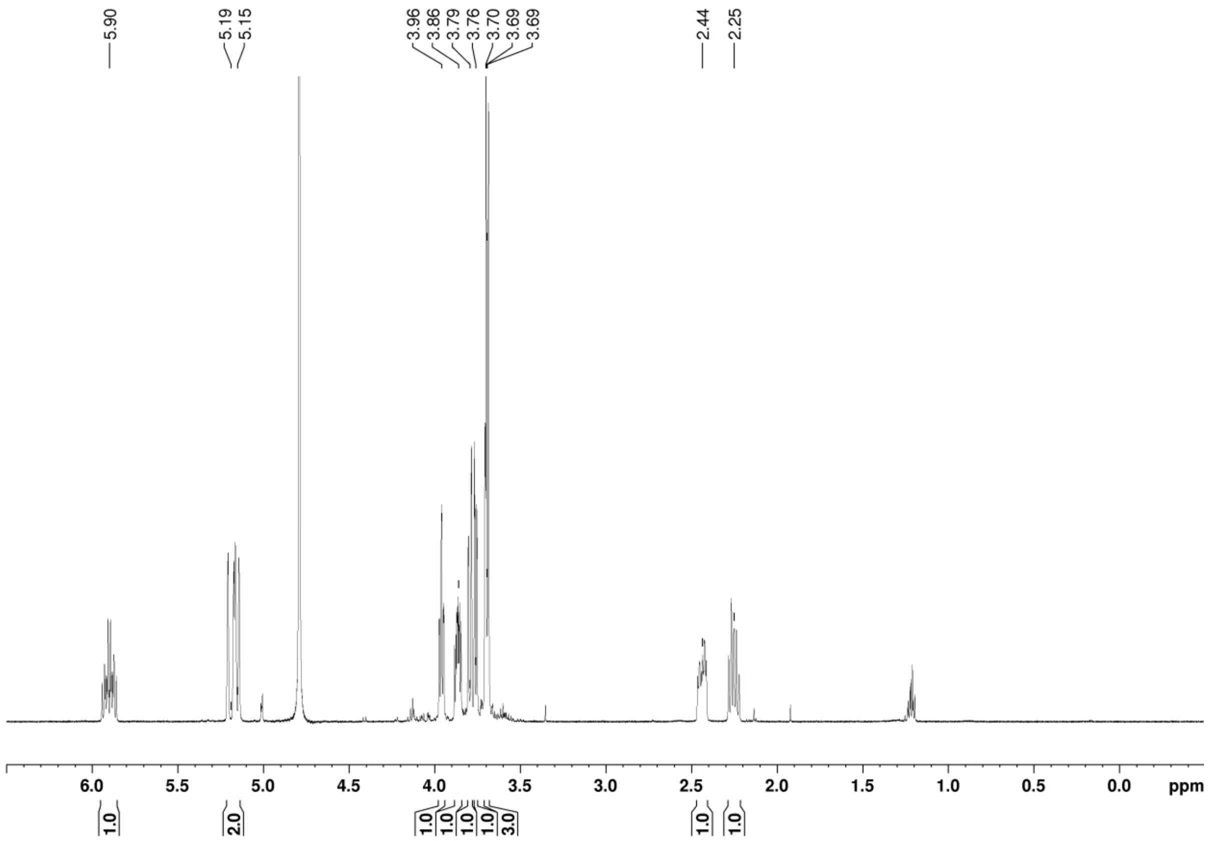

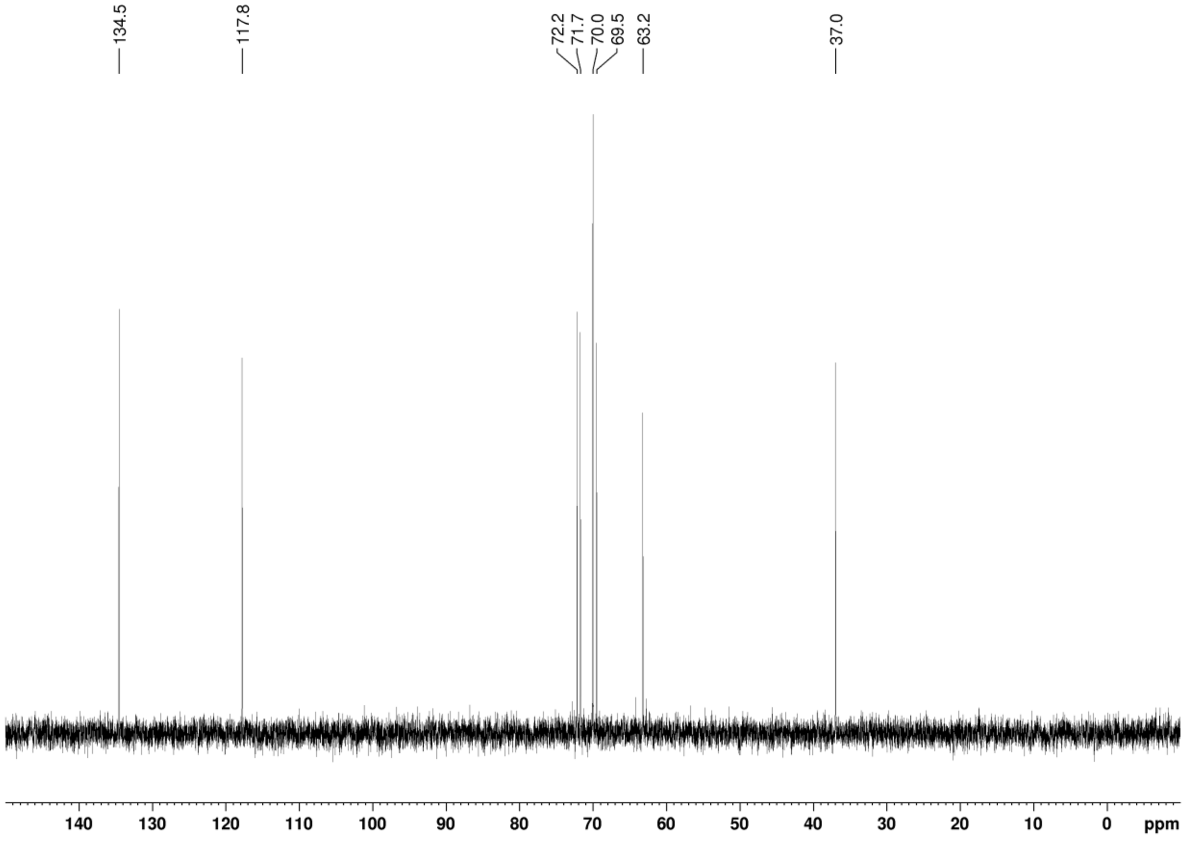

^{1}H-and ^{13}C NMR spectra of compound *e*-**Gal***

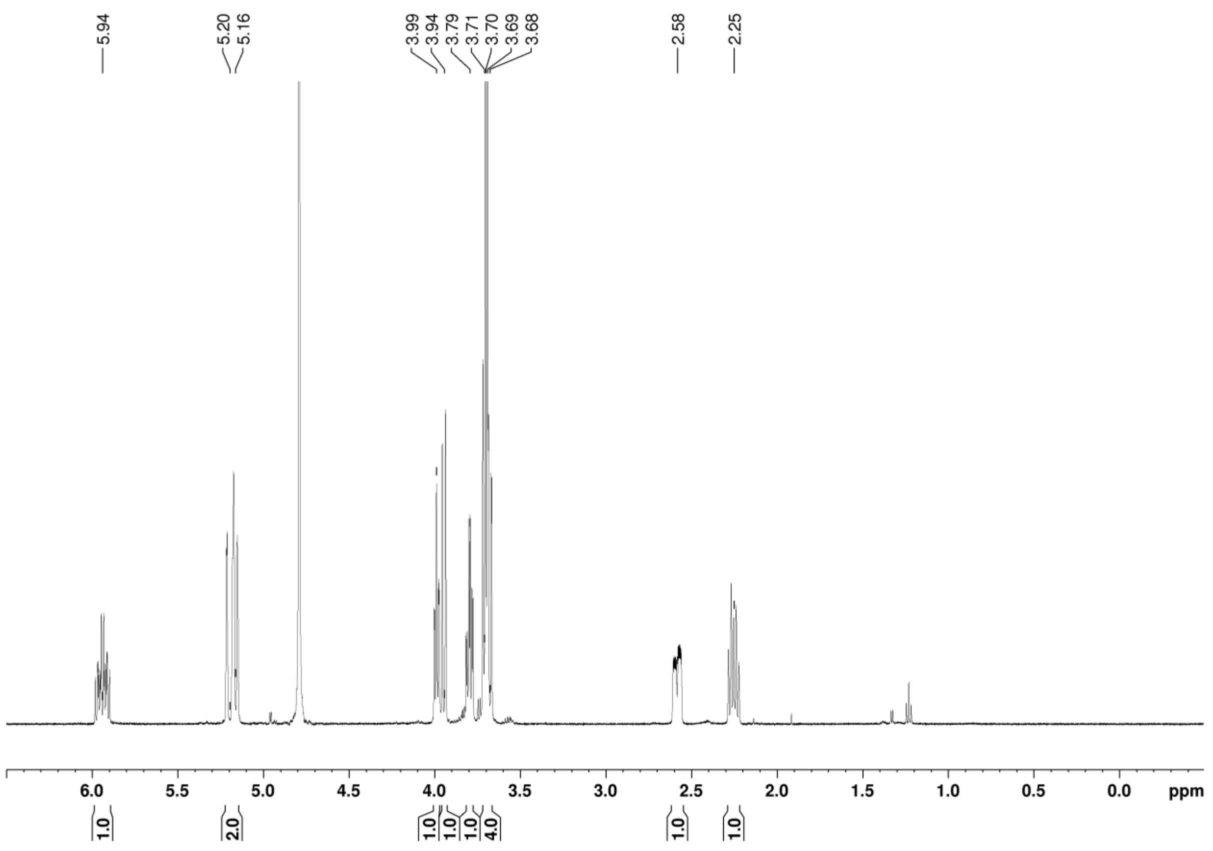

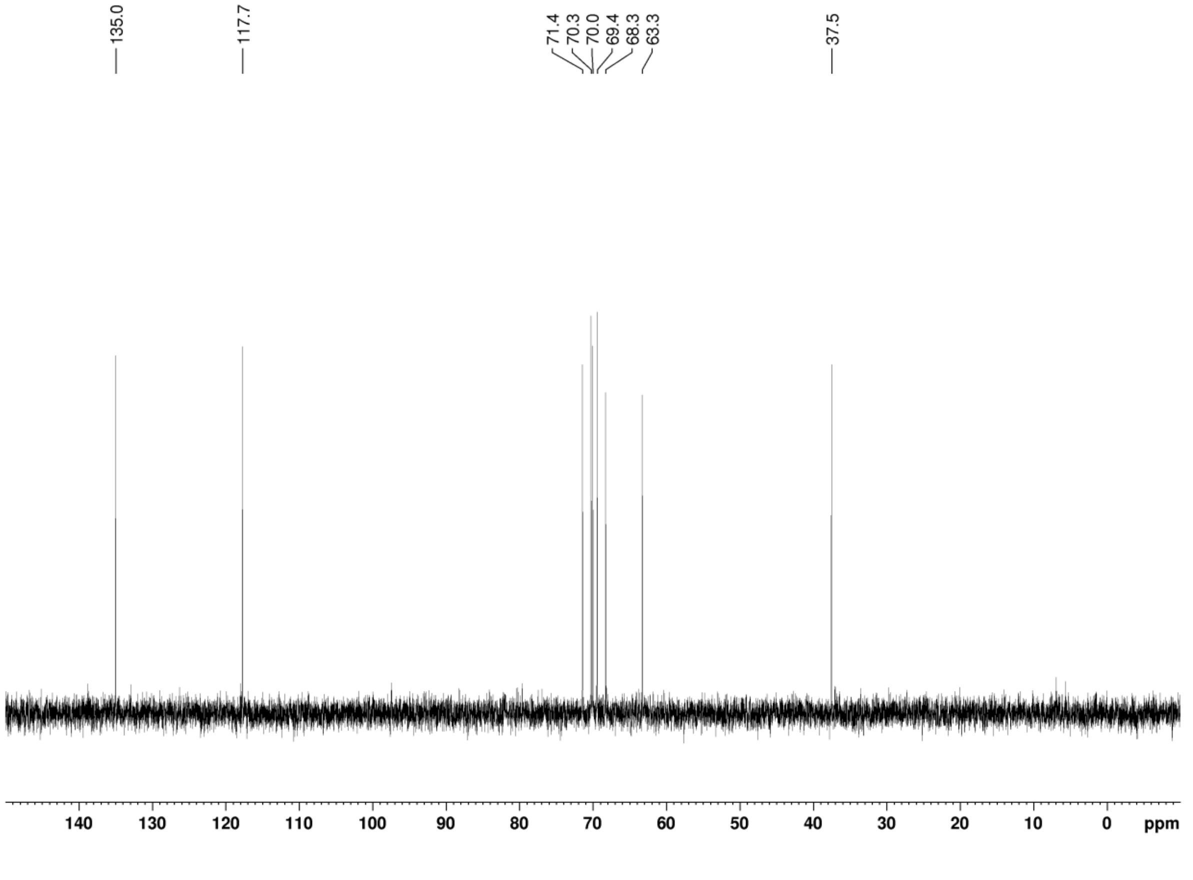

^{1}H-and ^{13}C NMR spectra of compound *t*-**Ara***

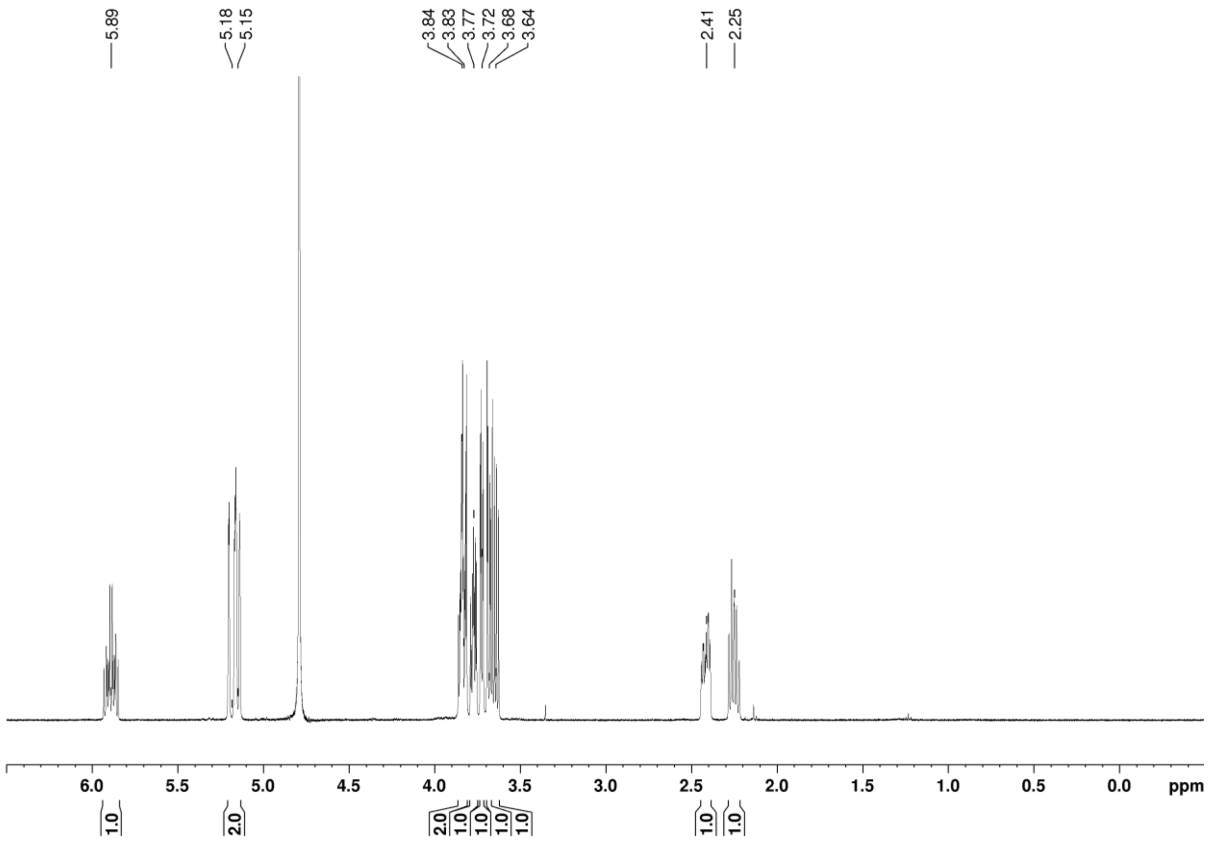

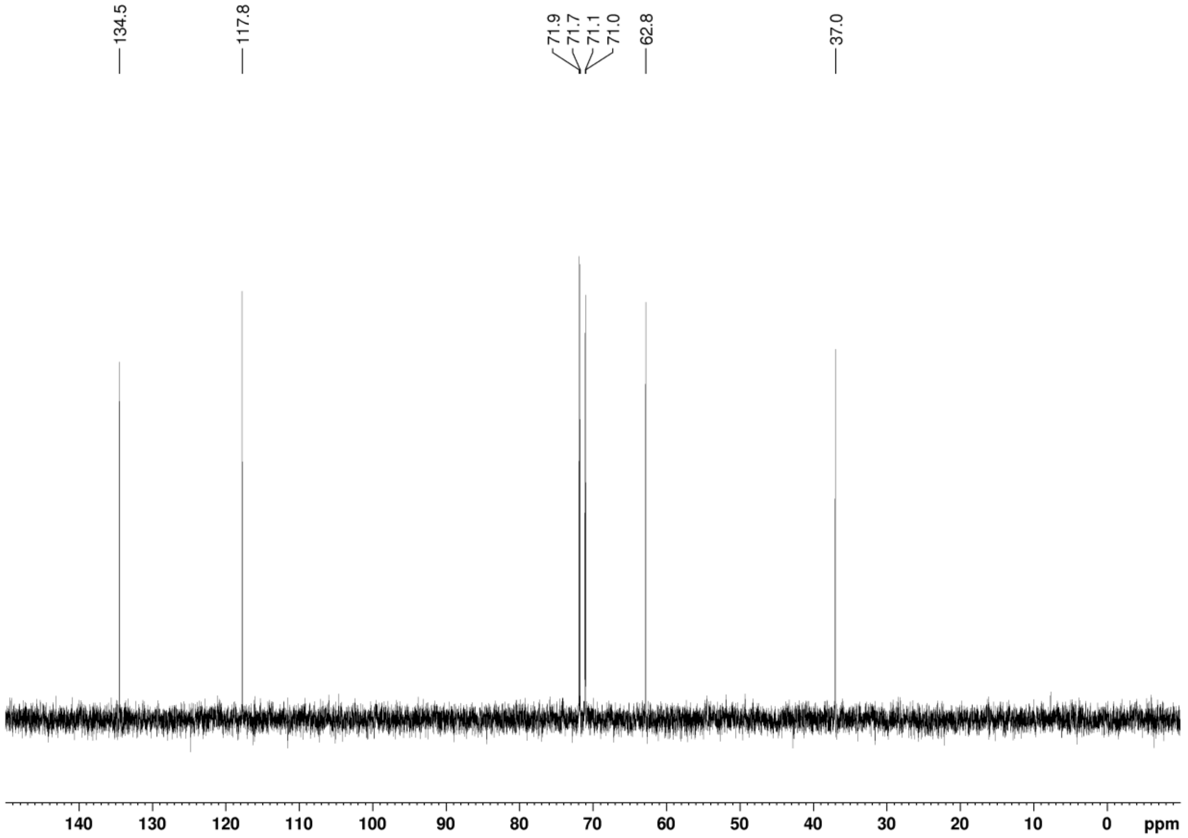

^{1}H-and ^{13}C NMR spectra of compound *e*-**Ara***

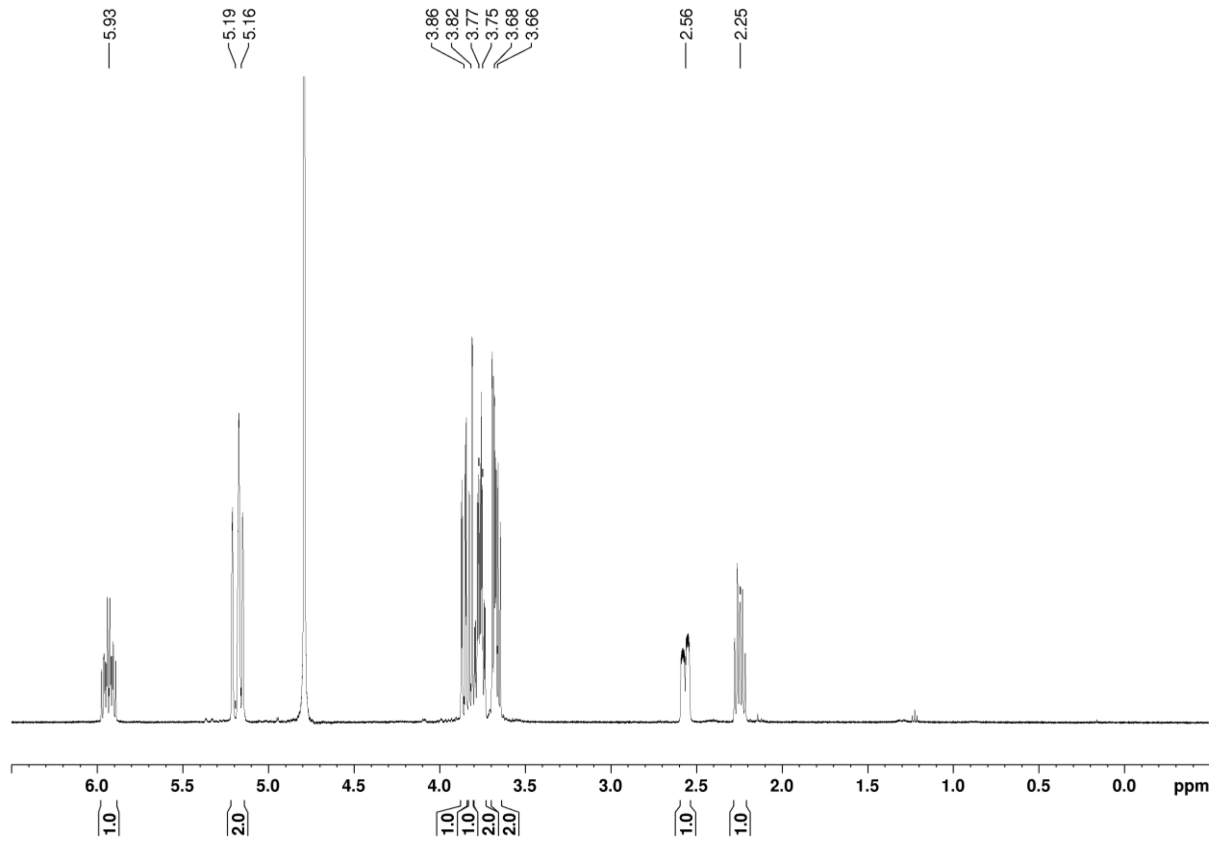

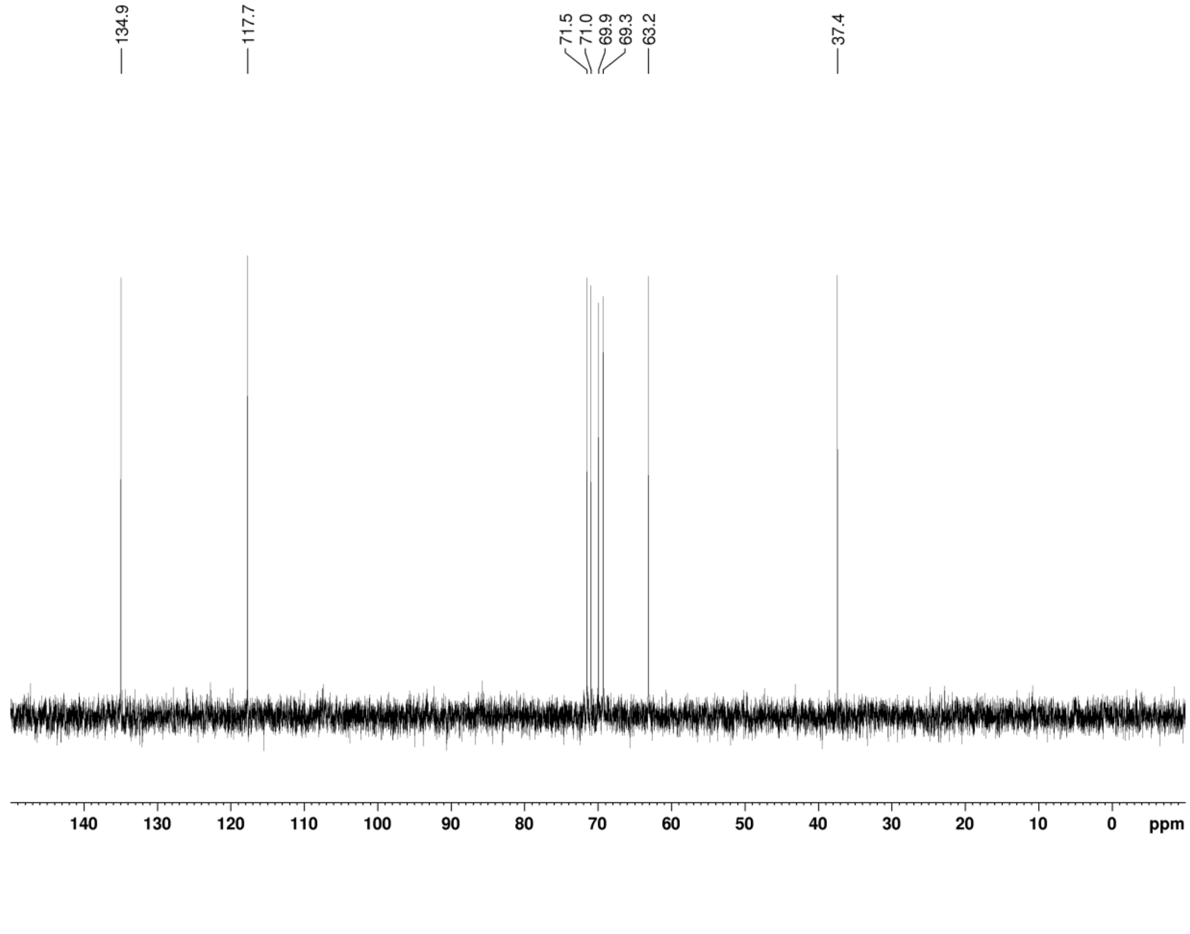

^{1}H-and ^{13}C NMR spectra of compound *t*-**Rha***

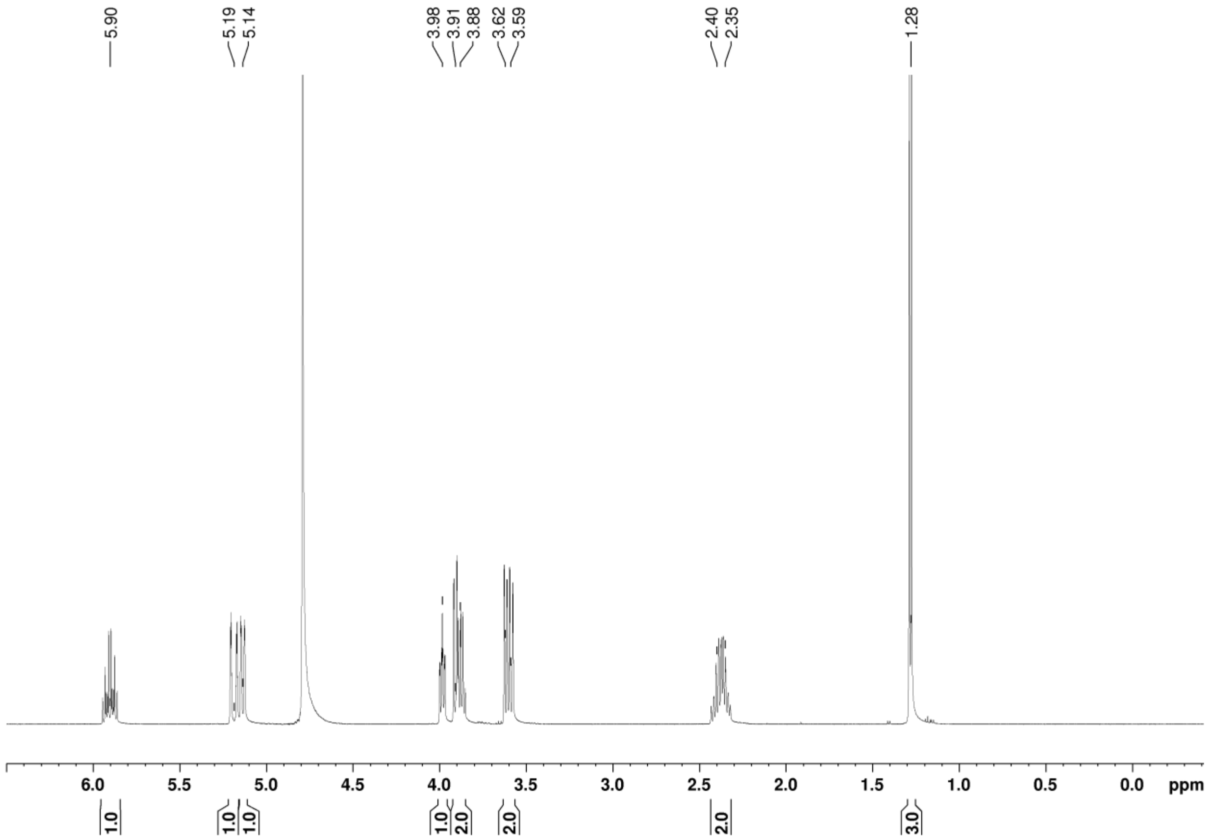

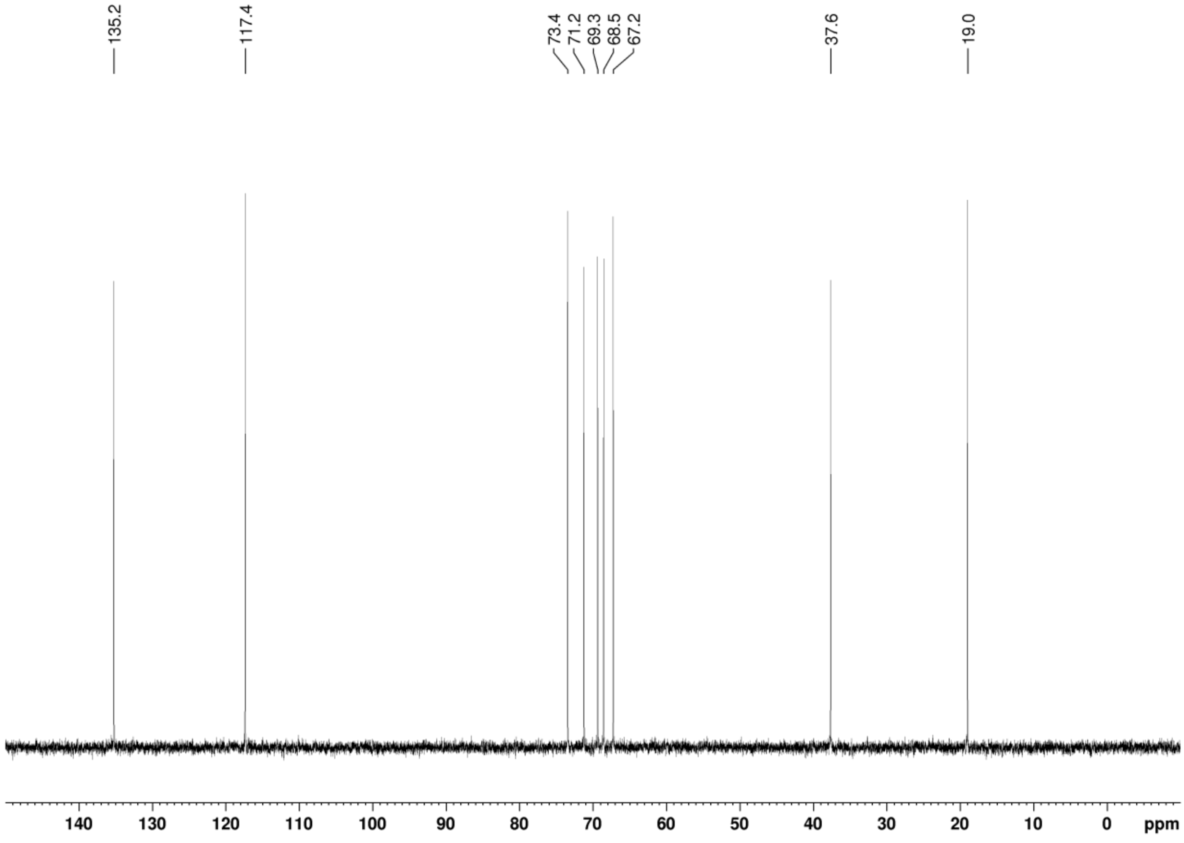

^{1}H-and ^{13}C NMR spectra of compound *t*-**Man*-SC6**

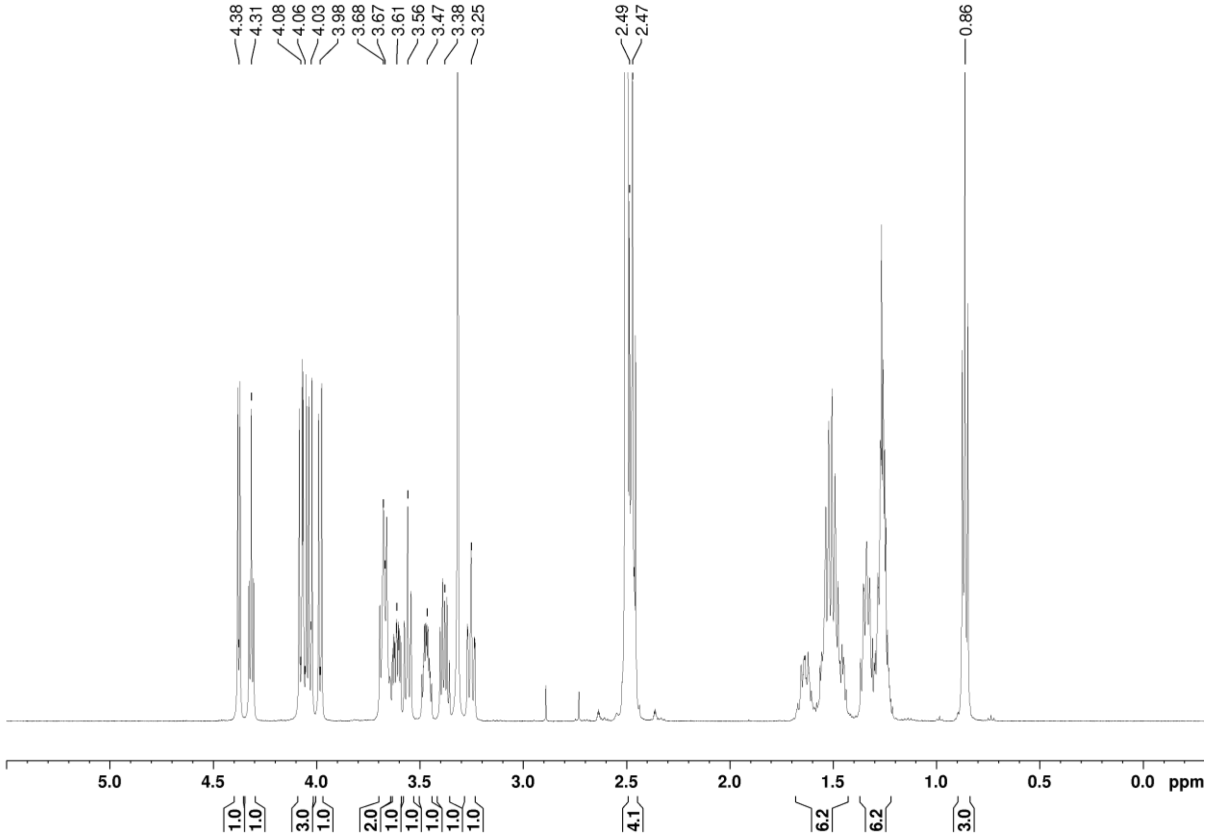

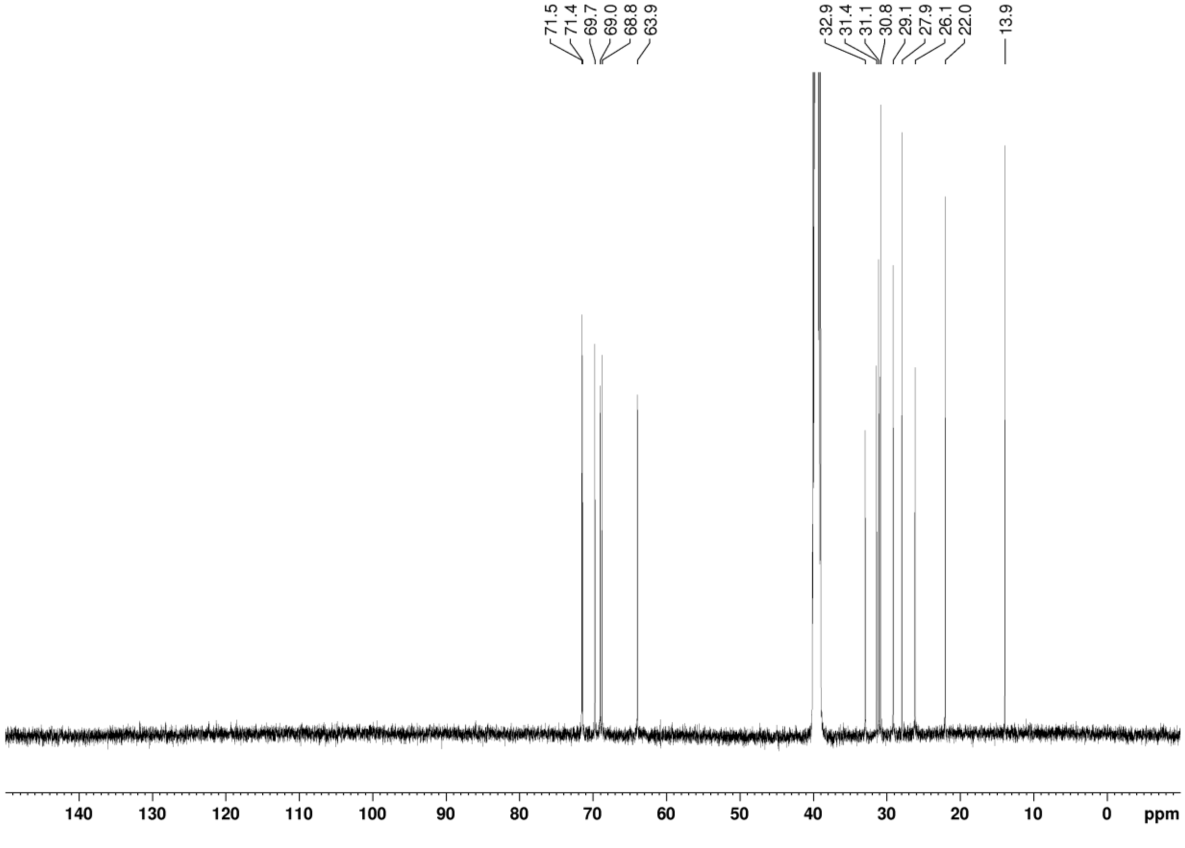

^{1}H-and ^{13}C NMR spectra of compound *t*-**Man*-SC10**

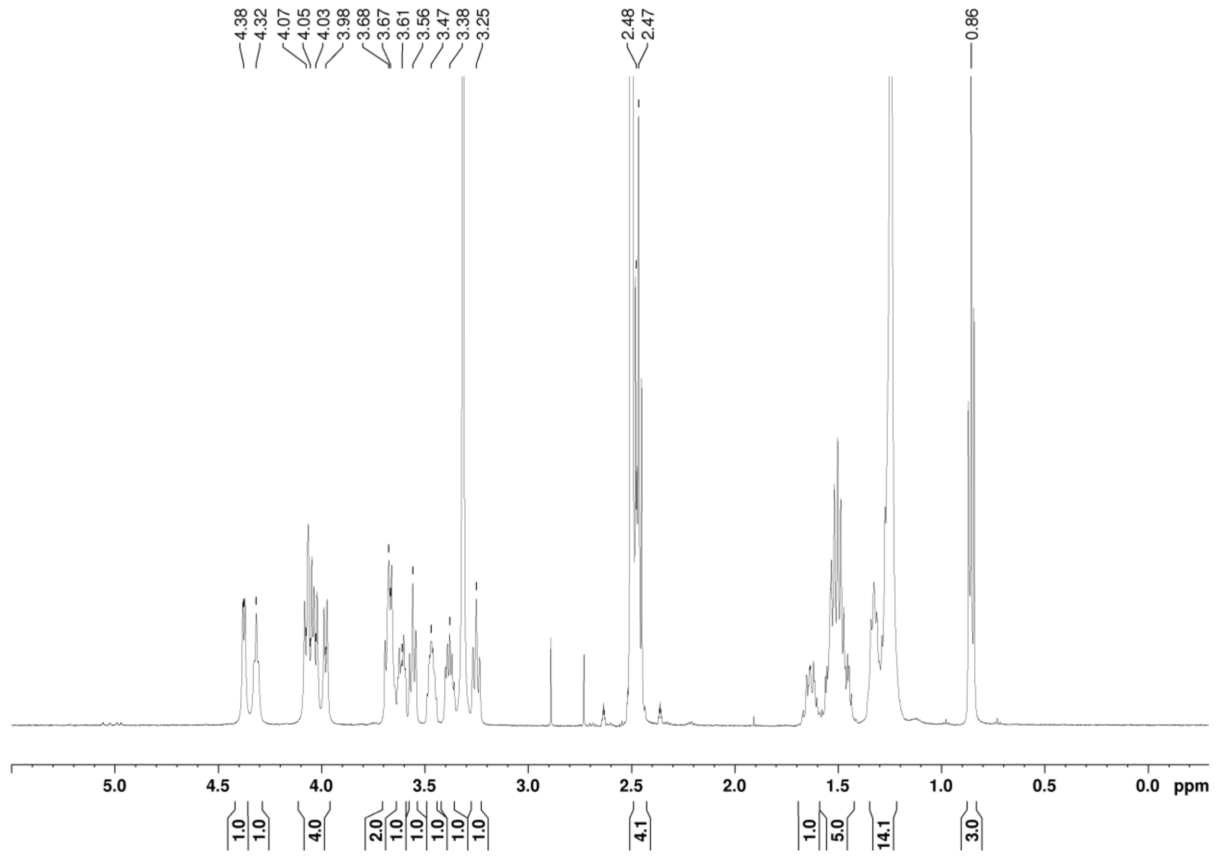

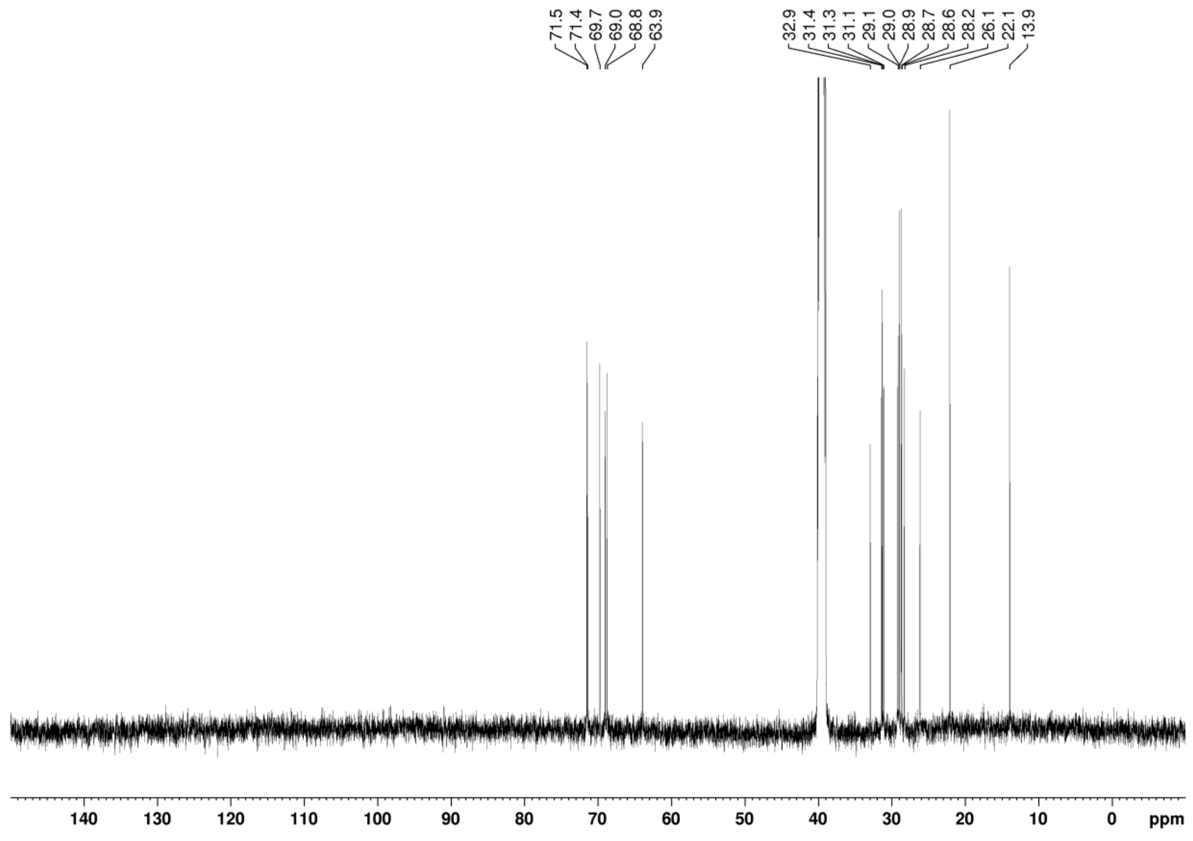

^{1}H-and ^{13}C NMR spectra of compound *t*-**Man*-SC14**

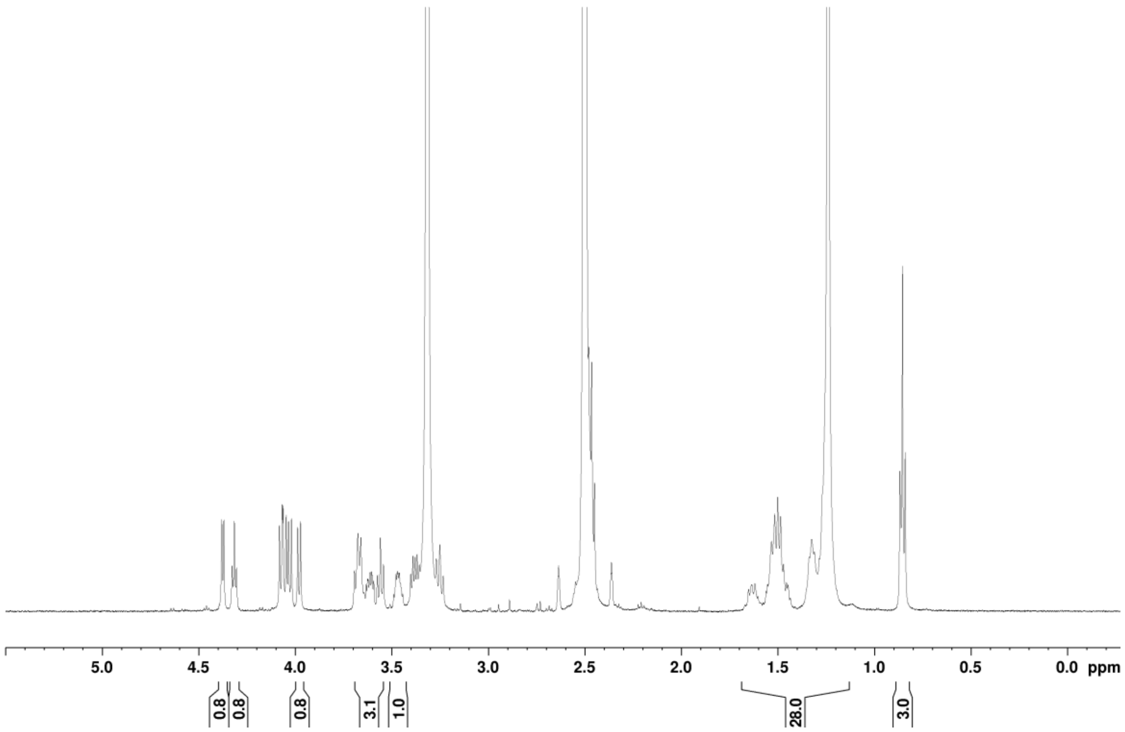

Due to the relatively high water content of this sample (as evidenced by the large signal at approx. 3.3 ppm), and the rapid exchange of the OH-protons with water, the OH-integrals at 4-4.5 ppm appear smaller than expected.

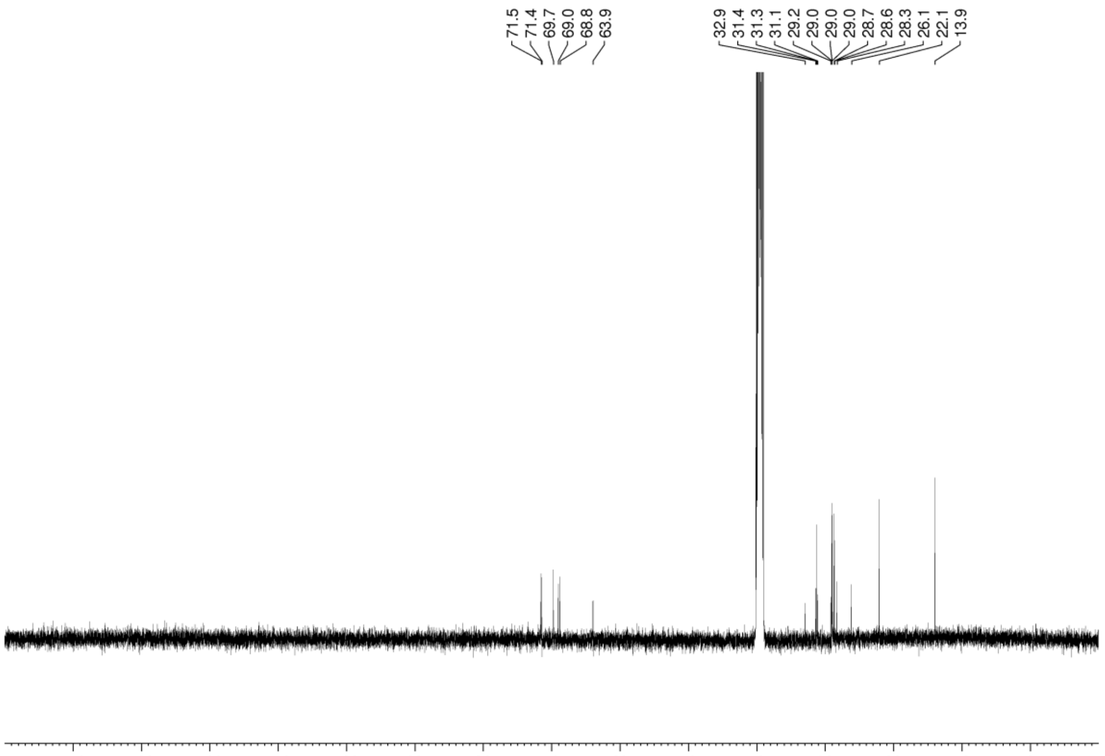

^{1}H-and ^{13}C NMR spectra of compound *t*-**Glc*-SC14**

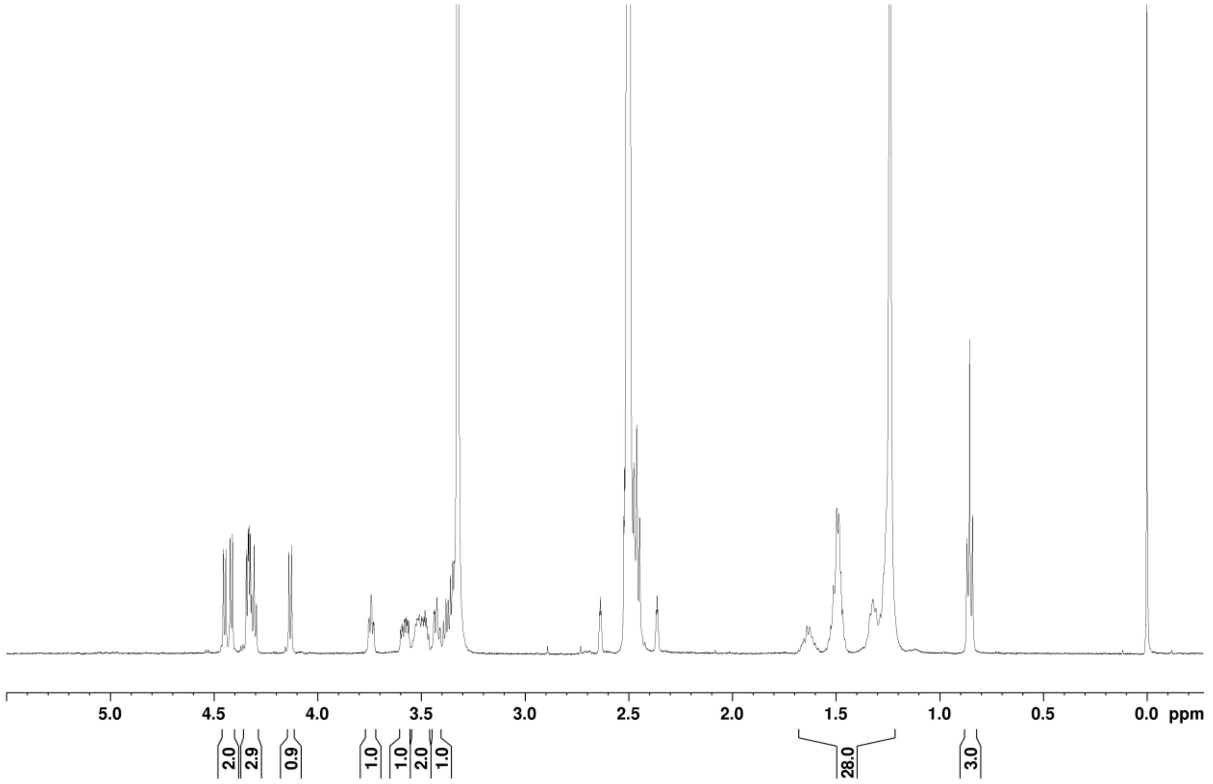

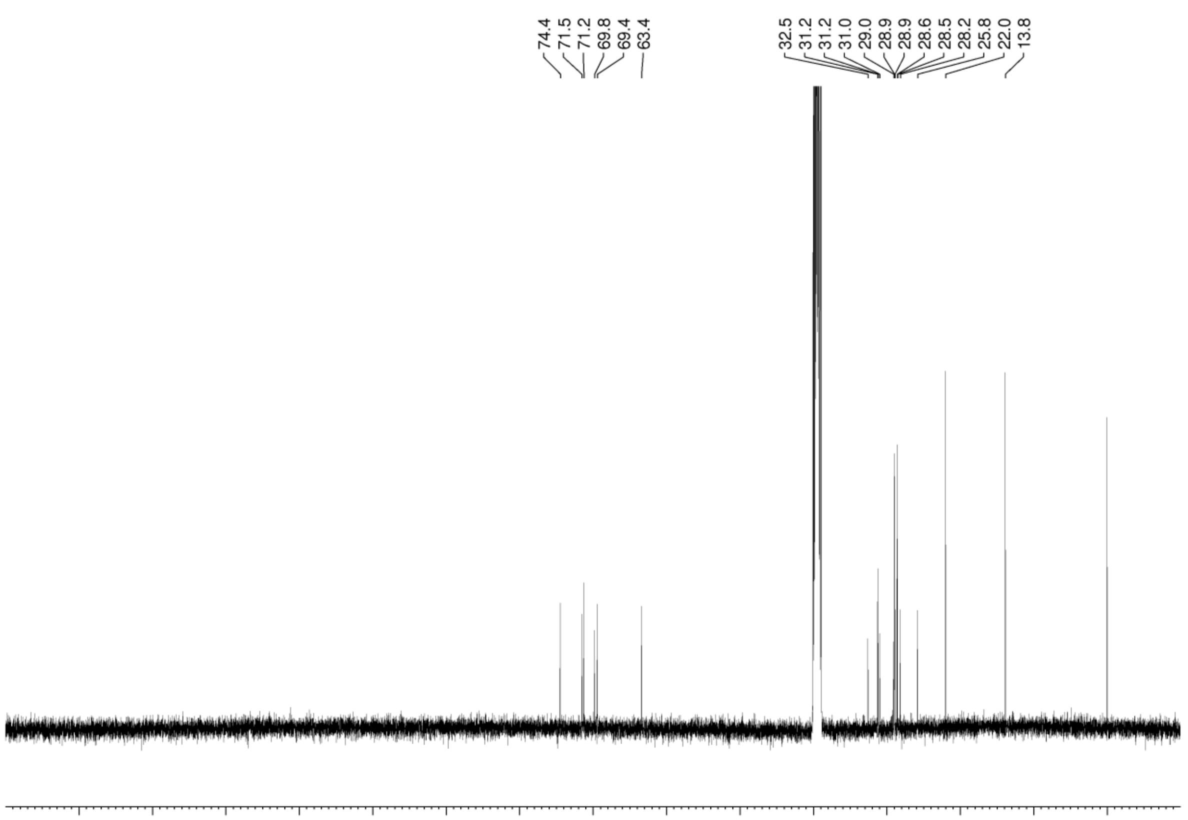

^{1}H-and ^{13}C NMR spectra of compound *e*-**Glc*-SC14**

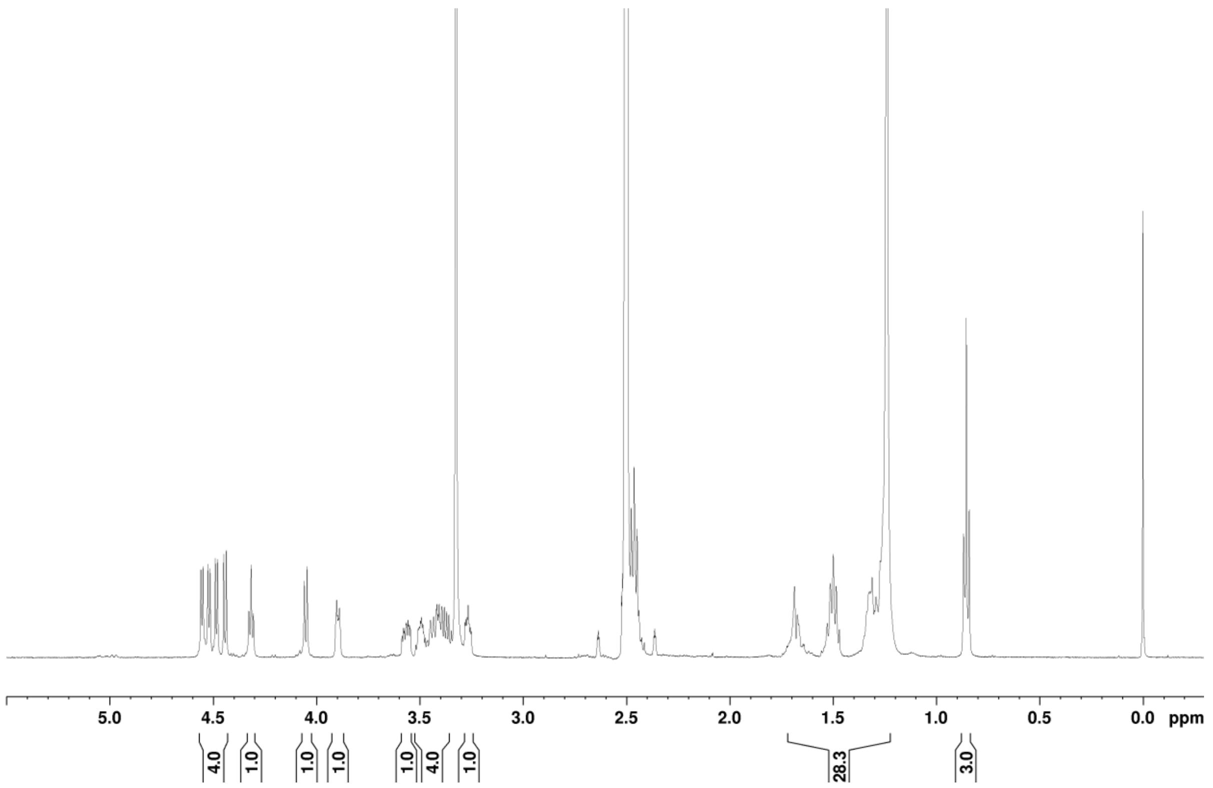

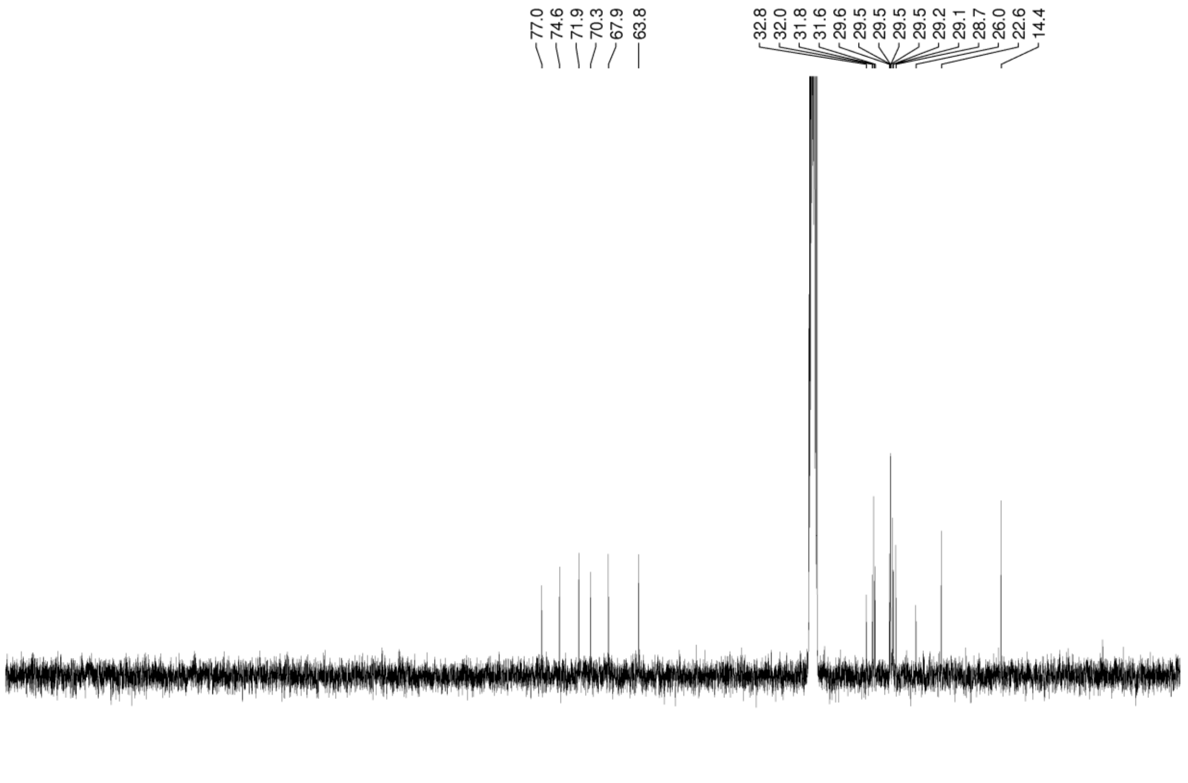

^{1}H-and ^{13}C NMR spectra of compound *t*-**Gal*-SC14**

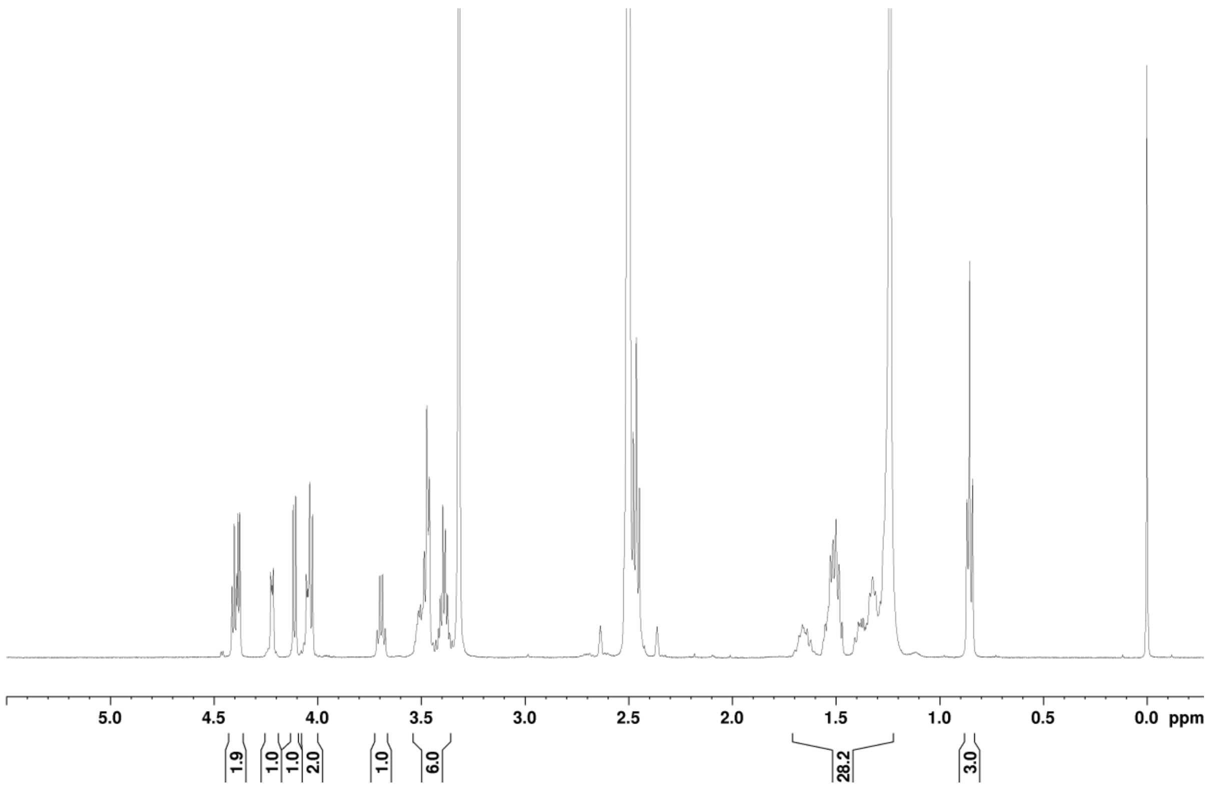

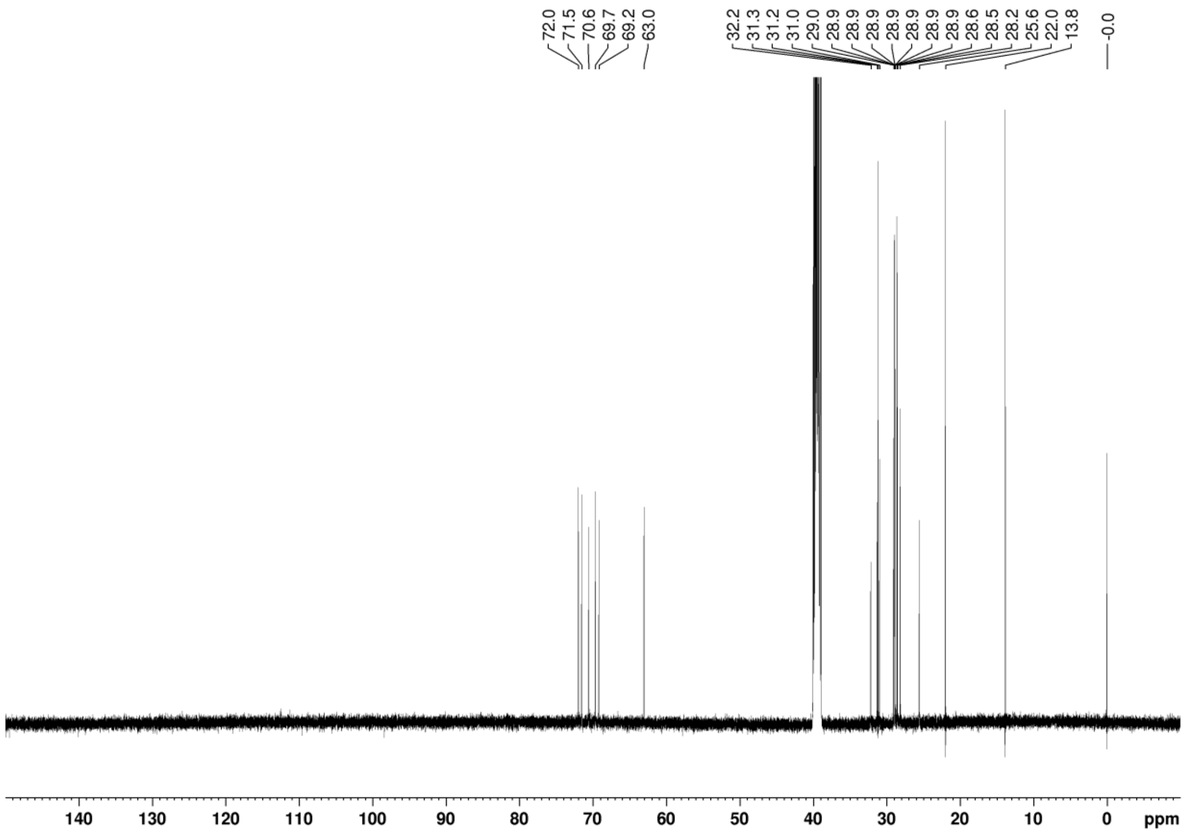

^{1}H-and ^{13}C NMR spectra of compound *e*-**Gal*-SC14**

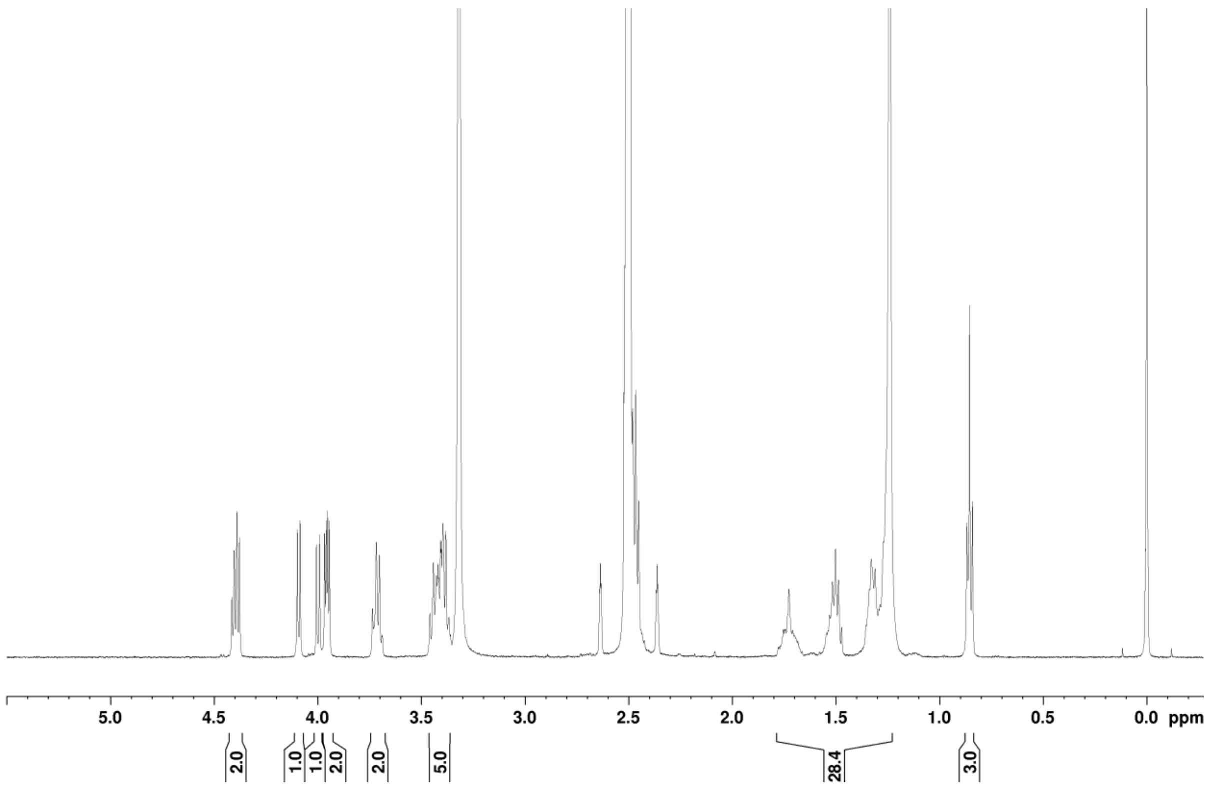

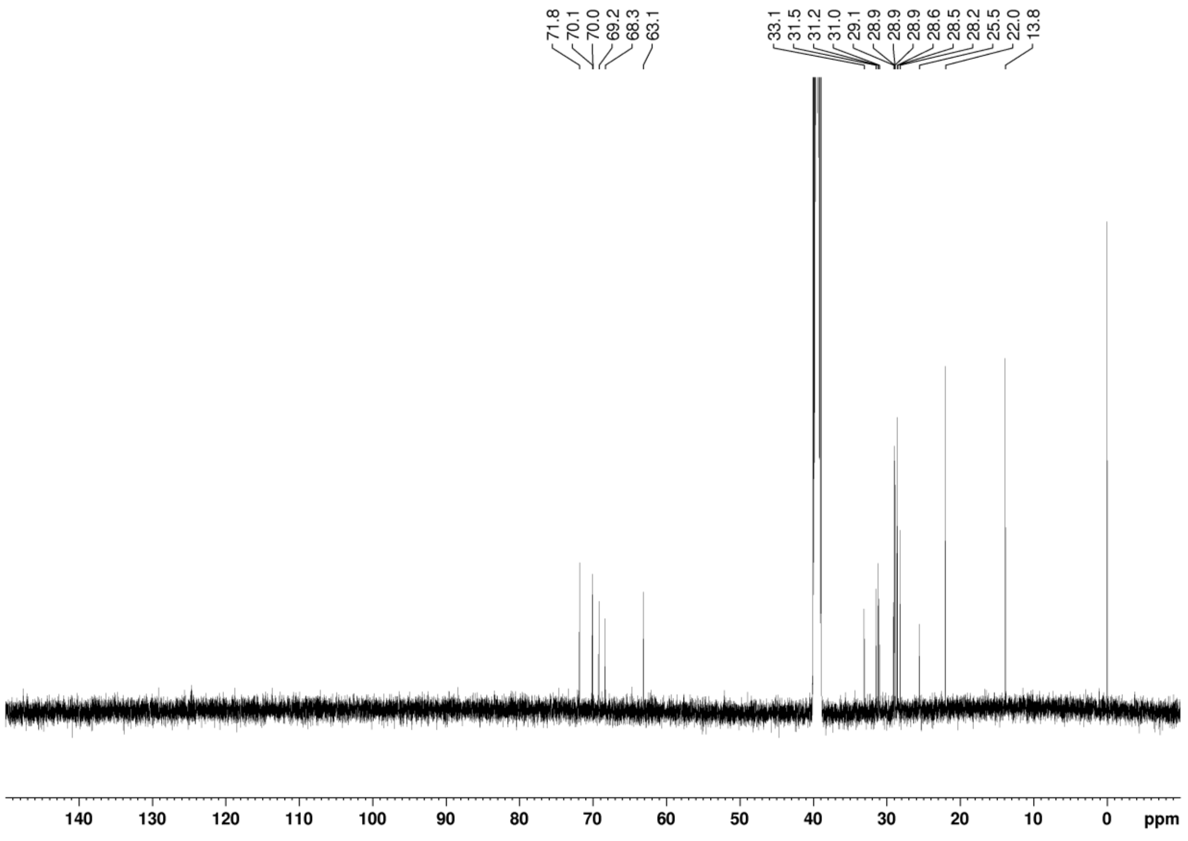

^{1}H-and ^{13}C NMR spectra of compound *t*-**Ara*-SC14**

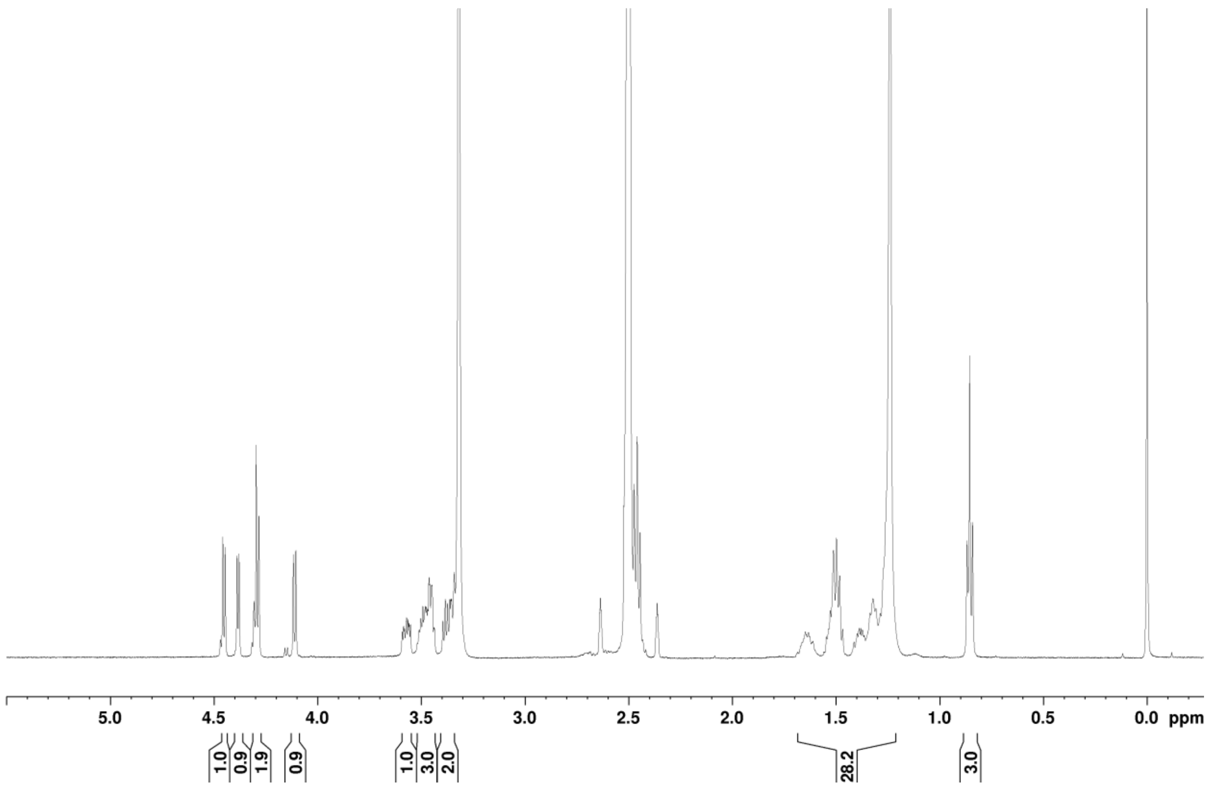

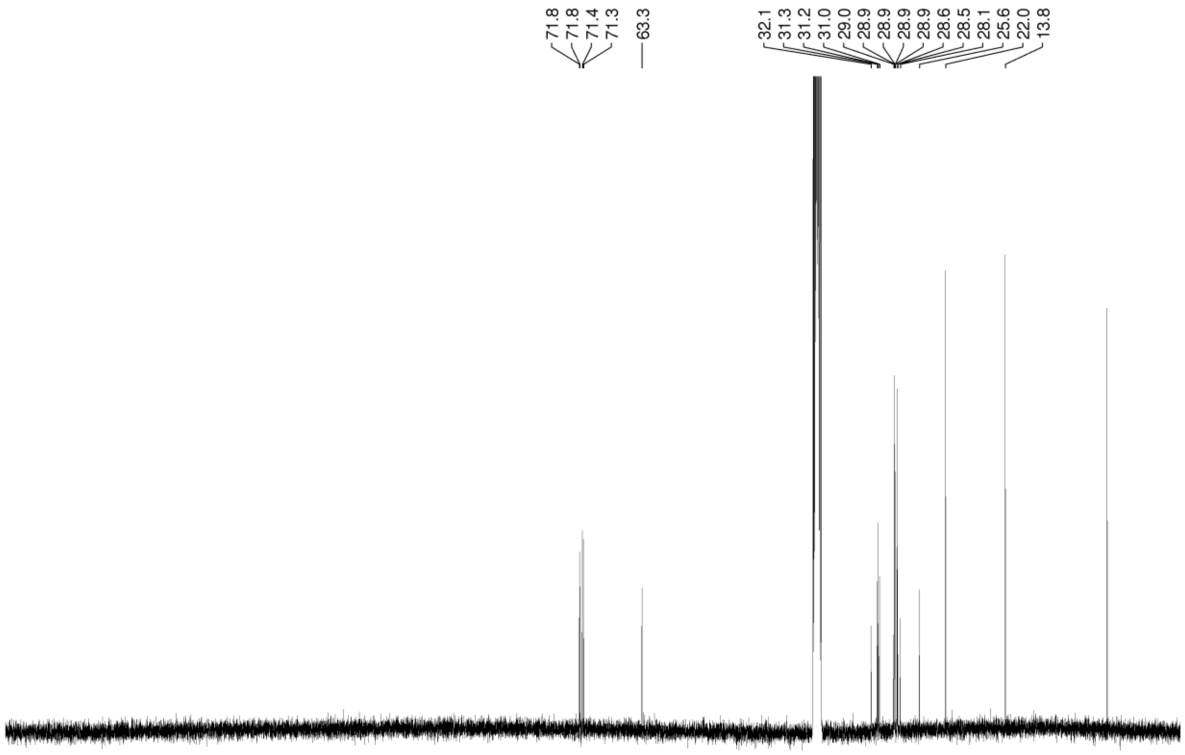

^{1}H-and ^{13}C NMR spectra of compound *e*-**Ara*-SC14**

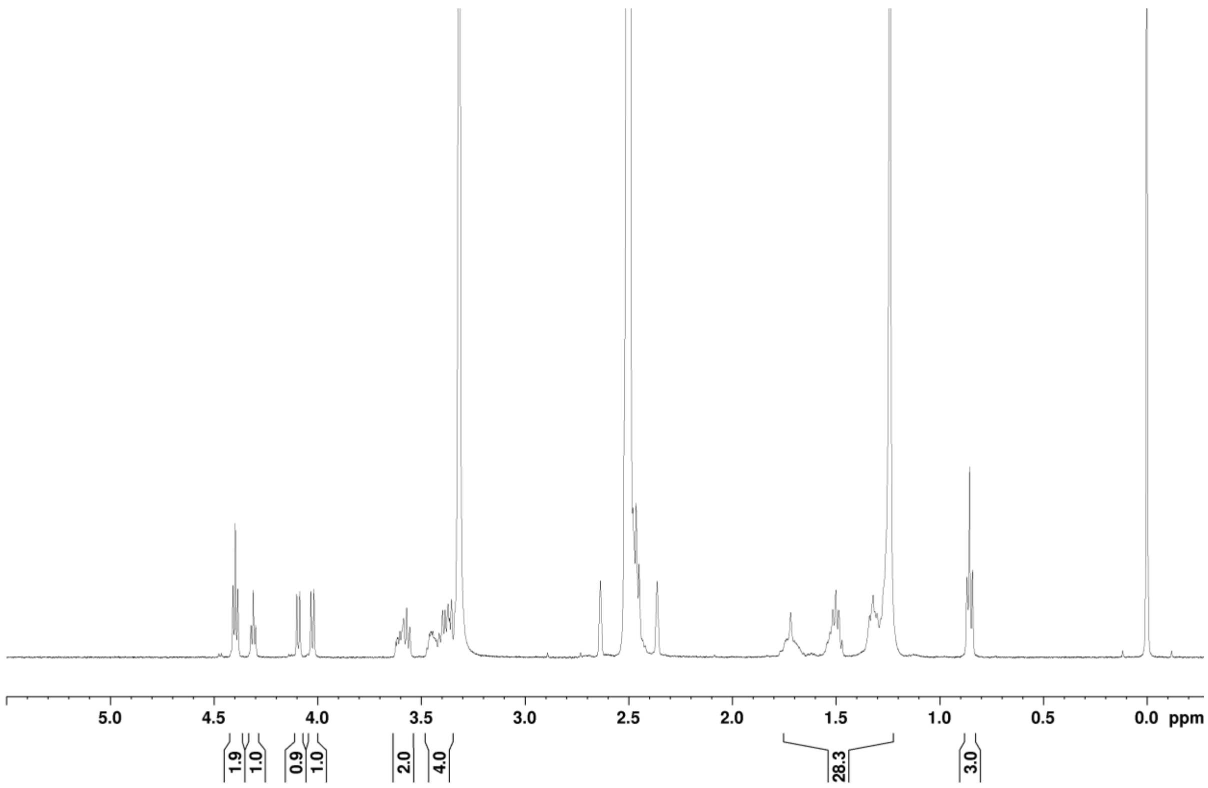

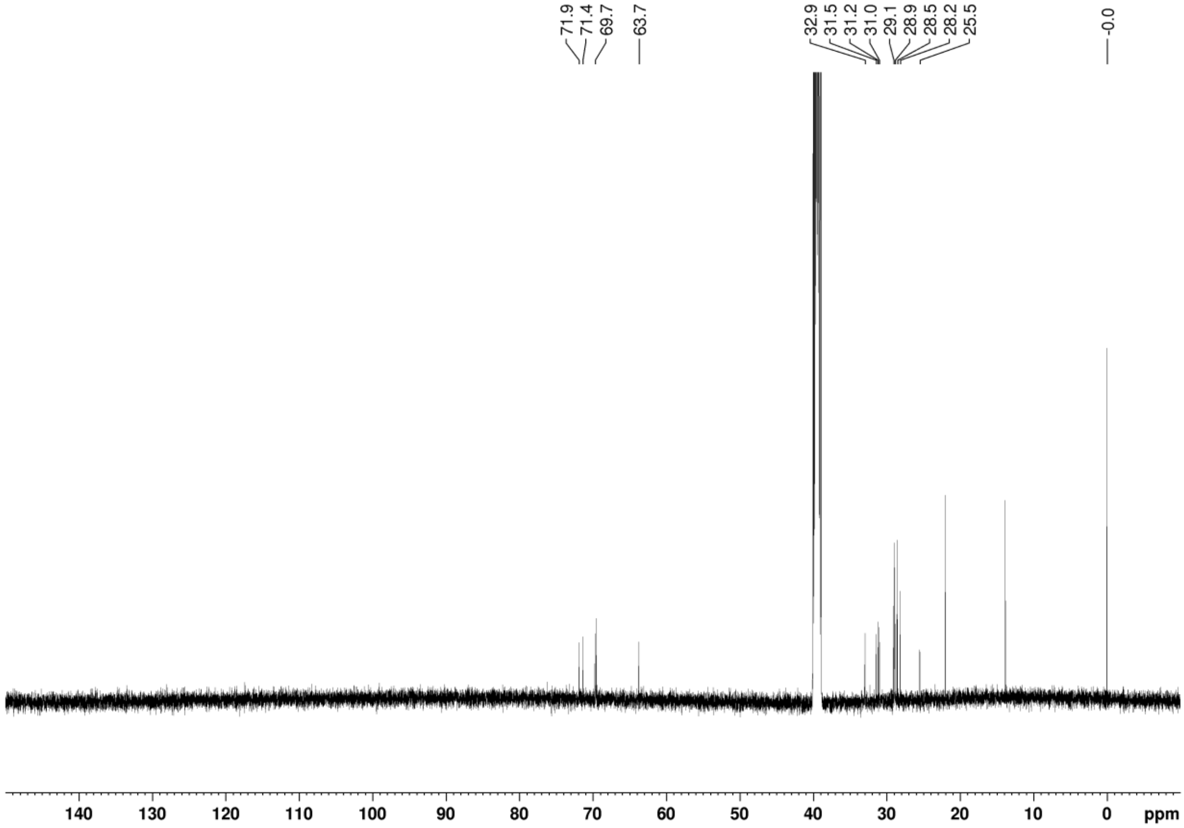

^{1}H-and ^{13}C NMR spectra of compound *t*-**Rha*-SC14**

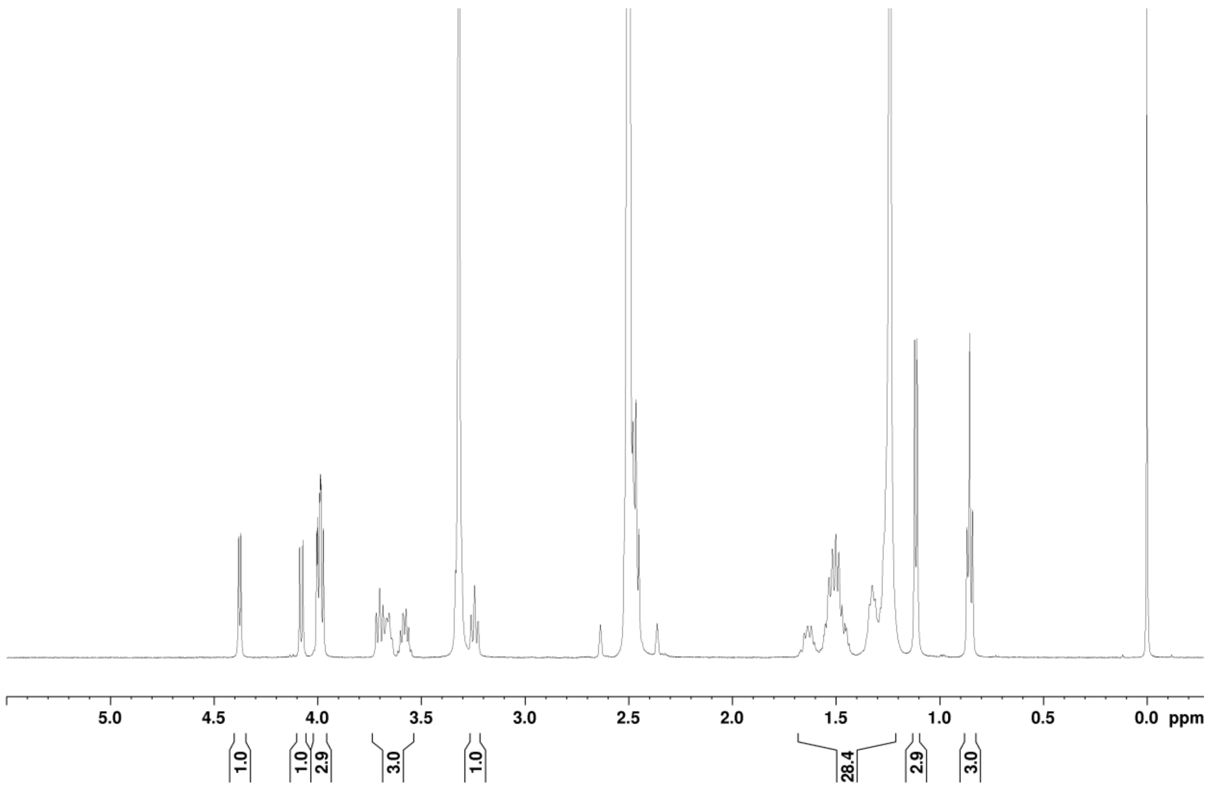

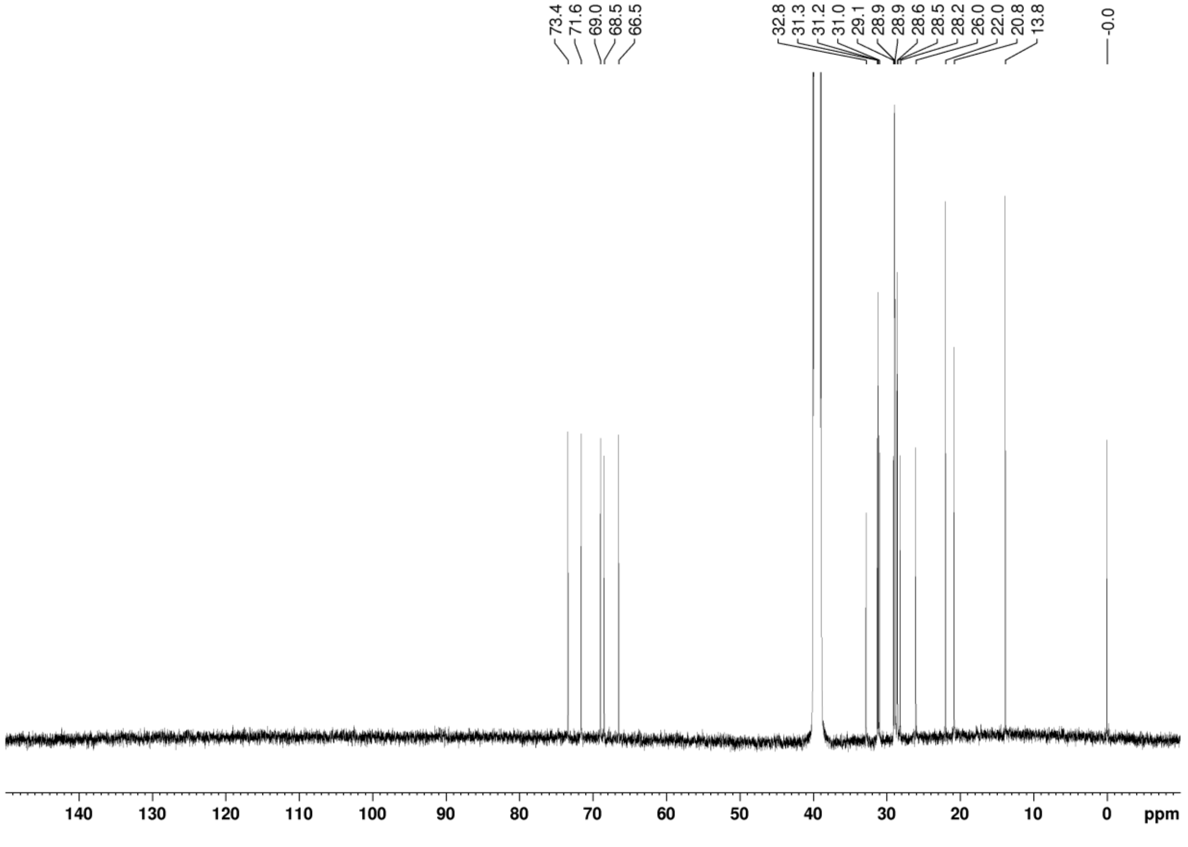

Conformational analysis of allylated monosaccharides

Geometry optimizations of the structures of *e*-**Glc***, *e*-**Gal***, *t*-**Ara***, *e*-**Ara*** and *t*-**Rha*** in water was conducted by a multi-level deterministic structural optimization using the Conformers module in the software Materials studio 2019 version 19.1.0[5,6] and the Jaguar program[7] in the software Maestro 12.2.[8] At molecular mechanics (MM) level, the Universal Force Field was applied as implemented in the Conformers module. The most stable conformers were optimized with density functional theory (DFT) applying the B3LYP hybrid functional,[9–12] the 6-31G** basis set and Grimme's method for DFT-D3 (dispersion) correction[13] as implemented in Jaguar. The surrounding medium might determine which conformation will be preferred. In order to corroborate this, the systems were studied in solution applying the COnductorlike Screening MOdel (COSMO)[14] with the dielectric constant 80.37 for water.

Table S1. Optimized geometries and NMR resolved $^{3}J_{H,H}$ coupling constants (Hz) in D_2O for compounds *t*-**Man***, *t*-**Glc***, *e*-**Glc***, *t*-**Gal*,** *e*-**Gal***, *t*-**Ara*,** *e*-**Ara***, *t*-**Rha***

Entry	Chemical structures and optimized geometries	$J_{2,3}$	$J_{3,4}$	$J_{4,5}$	$J_{5,6}$
1[a]	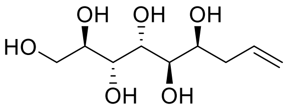 ***t*-Man*** 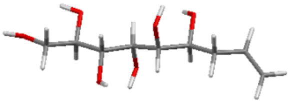	8.9	1.1	9.4	1.5
2[a]	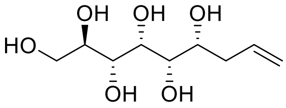 ***t*-Glc*** 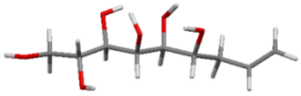	8.2	2.3	6.1	3.5
3	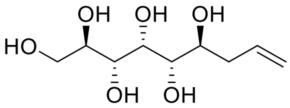 ***e*-Glc*** 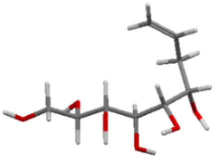	7.8	3.0	3.7	6.9
4[a]	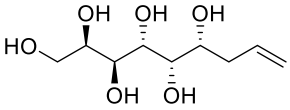 ***t*-Gal*** 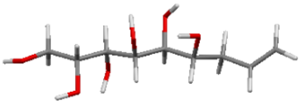	1.5	9.2	1.5	6.6
5	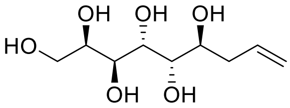 ***e*-Gal*** 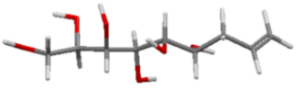	1.5	9.4	1.1	8.5
6	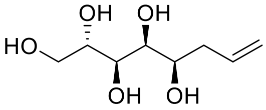 ***t*-Ara*** 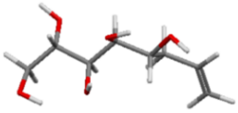	8.3	2.1	6.2	-
7	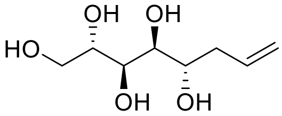 ***e*-Ara*** 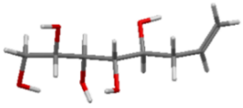	8.7	1.2	8.4	-
8	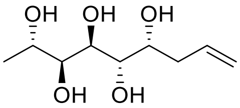 ***t*-Rha*** 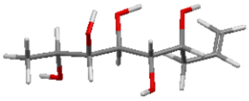	7.9	1.3	9.2	1.6

[a] Geometry optimization and NMR-data reported in reference 3.

Thermal analysis of allylated monosaccharides

Table S2. Melting points and melting enthalpies as determined by DSC, and molecular weights (for comparison) of compounds *t*-**Man***, *t*-**Glc***, *e*-**Glc***, *t*-**Gal***, *e*-**Gal***, *t*-**Ara***, *e*-**Ara***, *t*-**Rha***. The onset temperatures from the 1st heating cycle are reported.

**Compound**	**Melting point (°C)**	**Melting enthalpy (normalized, J/g)**	**Molecular weight (g/mol)**
t-**Man***	193.58	305.86	222.24
t-**Glc***	88.90	124.59	222.24
e-**Glc***	122.49	144.39	222.24
t-**Gal***	102.64	135.97	222.24
e-**Gal***	187.38	284.32	222.24
t-**Ara***	121.04	219.28	192.21
e-**Ara***	119.65	158.82	192.21
t-**Rha***	169.27	244.29	206.24

Thermal events for amphiphiles derived from allylated monosaccharides

Table S3. Thermal events for compounds *t*-**Man*-SC6,** *t*-**Man*-SC10** *t*-**Man*-SC14**, *t*-**Glc*-SC14**, *e*-**Glc*-SC14**, *t*-**Gal*-SC14**, *e*-**Gal*-SC14**, *t*-**Ara*-SC14**, *e*-**Ara*-SC14** *t*-**Rha*-SC14** observed with DSC.

**Compound**	**°C, (J/g)**	**Notes**
t-**Man*-SC6**	145.59, (8.54), T_{ss}<br>190.94, (169.94), T_m<br>198.33, (1.91), T_i<br>*cooling*<br>197.15, (-7.82), T_o<br>183.87, (-124.61), T_c	solid-state phase transition[a]<br>melting to smectic phase<br>isotropization<br><br>ordering to smectic phase<br>crystallization
t-**Man*-SC10**	137.09, (21.32), T_{ss}<br>186.29, (343.39), T_m<br>224.54, (6.38), T_i<br>*cooling*<br>224.94, (-7.46), T_o<br>178.44, (-111.62), T_c<br>90.79 (-14.17), T_{ss}	solid-state phase transition<br>melting to smectic phase<br>isotropization<br><br>ordering to smectic phase<br>crystallization<br>solid-state phase trasition
t-**Man*-SC14**	*1st heating*<br>169.71, (9.87), T_m<br>175.02, (-6.43), T_c<br>183.21 (113.07), T_m<br>231.49 (2.79), T_i<br>*1st cooling*<br>230.06 (-2.92), T_o<br>177.74 (-109.38), T_c<br>*2nd heating*<br>183.0, (121.26), T_m<br>229.25, (2.81), T_i<br>*2nd cooling*<br>228.15, (-2.61), T_o<br>177.33, (-105.04), T_c	 melting of minor polymorph[a]<br>re-crystallization to main phase[a]<br>melting to smectic phase<br>isotropization<br><br>ordering to smectic phase<br>crystallization<br><br>melting to smectic phase<br>isotropization<br><br>ordering to smectic phase<br>crystallization
t-**Glc*-SC14**	*1st heating*<br>104.28 (168.56) T_m<br>213.23 (3.18) T_i<br>*1st cooling*<br>211.53 (-3.15) T_o<br>104.28 (-122.01) T_c<br>*2nd heating*<br>102.83, (131.64), T_m<br>208.99, (3.03), T_i<br>*2nd cooling*<br>206.77, (-2.70), T_o<br>55.58, (-102.70), T_c	 melting to smectic phase<br>isotropization<br><br>ordering to smectic phase<br>crystallization<br><br>melting to smectic phase<br>isotropization<br><br>ordering to smectic phase<br>crystallization
e-**Glc*-SC14**	*1st heating*<br>83.47 (41.53) T_{cub}<br>125.94 (101.04) T_m<br>220.33 (3.01) T_i<br>*1st cooling*	 transition to cubic phase<br>melting to smectic phase<br>isotropization

	217.68 (-2.89) T_o<br>122.10 (-100.69) T_{cub}<br>77.98 (-4.89) T_c<br>69.46 (-23.36) T_{ss}<br>*2nd heating*<br>31.07, (2.67), T_{ss}<br>84.45, (38.34), T_{cub}<br>121.93, (72.92), T_m<br>209.91, (2.76), T_i<br>*2nd cooling*<br>206.97, (-2.16), T_o<br>115.93, (-64.15), T_{cub}<br>78.51, (-15.84), T_c<br>34.25, (-17.21), T_{ss}	ordering to smectic phase<br>transition to cubic phase<br>crystallization<br>solid-state transition<br><br>solid-state transition<br>transition to cubic phase<br>melting to smectic phase<br>isotropization<br><br>ordering to smectic phase<br>transition to cubic phase<br>crystallization<br>solid-state transition
t-**Gal*-SC14**	*1st heating*<br>103.24 (35.87), T_{ss}<br>120.52 (98.11), T_m<br>216.65 (3.03), T_i<br>*1st cooling*<br>216.06 (-3.02), T_o<br>104.10 (-90.00), T_c<br>54.79 (-37.24), T_{ss}<br>*2nd heating*<br>52.62 (37.33), T_{ss}<br>118.24 (91.57), T_m<br>212.04, (1.81), T_i<br>*2nd cooling*<br>211.83, (-2.37), T_o<br>97.63, (-75.18), T_c<br>56.51, (-29.07), T_{ss}	 solid-state transition/minor phase melting[a]<br>melting to smectic phase<br>isotropization<br><br>ordering to smectic phase<br>crystallization<br>solid-state phase transition<br><br>solid-state transition<br>melting to smectic phase<br>isotropization<br><br>ordering to smectic phase<br>crystallization<br>solid-state transition
e-**Gal*-SC14**	*1st heating*<br>54.91 (4.25), T_{ss}<br>127.89 (33.83), T_{cub}<br>173.69 (122.08), T_m<br>225.70 (2.84), T_i<br>*1st cooling*<br>216.56 (-2.34), T_o<br>154.17 (-78.40), T_{cub}<br>116.73 (-25.04), T_c<br>56.15 (-3.68), T_{ss}<br>42.49, (-6.13), T_{ss}<br>*2nd heating*<br>33.64, (5.48), T_{ss}<br>55.34, (4.50), T_{ss}<br>129.13, (20.84), T_{cub}<br>164.68, (80.35), T_m<br>207.20, (2.17), T_i<br>*2nd cooling*<br>183.19, (-2.22), T_o<br>137.83, (-42.64), T_{cub}	 solid-state transition<br>transition to cubic phase<br>melting to smectic phase<br>isotropization<br><br>ordering to smectic phase<br>transition to cubic phase<br>crystallization<br>solid-state transition<br>solid-state transition<br><br>solid-state transition<br>solid-state transition<br>transition to cubic phase<br>melting to smectic phase<br>isotropization<br><br>ordering to smectic phase<br>transition to cubic phase

	116.99, (-4.91), T_c<br>56.55, (-2.01), T_{ss}<br>35.03, (-20.94), T_{ss}	crystallization<br>solid-state transition<br>solid-state transition
t-**Ara*-SC14**	*1st heating*<br>74.23 (10.91) T_m<br>83.72 (119.37) T_m<br>184.67 (3.32) T_i<br>*1st cooling*<br>183.64 (-2.83) T_o<br>68.96 (-100.95) T_c<br>*2nd heating*<br>74.78, (1.66), T_m<br>85.14, (95.74), T_m<br>184.00, (2.78), T_i<br>*2nd cooling*<br>183.32, (-2.84), T_o<br>68.09, (-95.26), T_c	 melting of minor polymorph phase<br>melting to smectic phase<br>isotropization<br><br>ordering to smectic phase<br>crystallization<br><br>melting of minor polymorph phase<br>melting to smectic phase<br>isotropization<br><br>ordering to smectic phase<br>crystallization
e-**Ara*-SC14**	*1st heating*<br>121.24 (119.95) T_m/ T_{ss}<br>124.67 (86.13) T_m<br>192.47 (2.56) T_i<br>*1st cooling*<br>191.69 (-2.64) T_o<br>119.14 (-116.91) T_{cub}<br>55.52 (-5.21) T_c<br>*2nd heating*<br>71.55 (20.16) T_{cub}<br>124.24 (119.15) T_m<br>191.95 (2.49) T_i<br>*2nd cooling*<br>191.21, (-2.58), T_o<br>118.74, (-114.03), T_{cub}<br>53.09, (-2.87), T_c	 melting of a polymorph or solid-state transition trans[a]<br>melting to smectic phase<br>isotropization<br><br>ordering to smectic phase<br>transition to cubic phase<br>crystallization/solid-state transition<br><br>transition to cubic phase<br>melting to smectic phase<br>isotropization<br><br>ordering to smectic phase<br>transition to cubic<br>crystallization/solid-state transition
t-**Rha*-SC14**	*1st heating*<br>59.11 (20.78), T_{ss}<br>97.15 (14.30), T_{ss}<br>119.83 (19.74), T_{cub}<br>148.49 (40.03), T_{pre}<br>167.07 (57.01), T_m<br>174.40 (9.78), T_i<br>*1st cooling*<br>173.84 (-9.50), T_o<br>168.25 (-51.99), T_{pre}<br>127.31 (-25.19), T_{cub}<br>103.60 (-31.45), T_c<br>*2nd heating*<br>71.47, (1.42), T_{ss}<br>98.53, (1.63), T_{ss}<br>135.09, (16.45), T_{cub}	 solid-state transition[a]<br>solid-state transition<br>transition to cubic phase<br>transition to pre-layered phase<br>melting to smectic phase<br>isotropization<br><br>ordering to smectic phase<br>transition to pre-layered phase<br>transition to cubic phase<br>crystallization/solid-state transition<br><br>solid-state transition<br>solid-state transition<br>transition to cubic phase

	148.89, (12.49), T_{pre}<br>167.05, (57.77), T_m<br>173.89, (9.73), T_i<br>*2nd cooling*<br>173.67, (-9.57), T_o<br>168.18, (-51.54), T_{pre}<br>127.27, (-22.71), T_{cub}<br>103.42, (-7.81), T_c	transition to pre-layered phase<br>melting to smectic phase<br>isotropization<br><br>ordering to smectic phase<br>transition to pre-layered phase<br>transition to cubic phase<br>crystallization/solid-state transition

All thermal transition taken as an extrapolated onset value, [a] = not seen on 2nd heating run.

DSC curves for amphiphiles derived from allylated monosaccharides

DSC curve for *t*-**Man*-SC6**

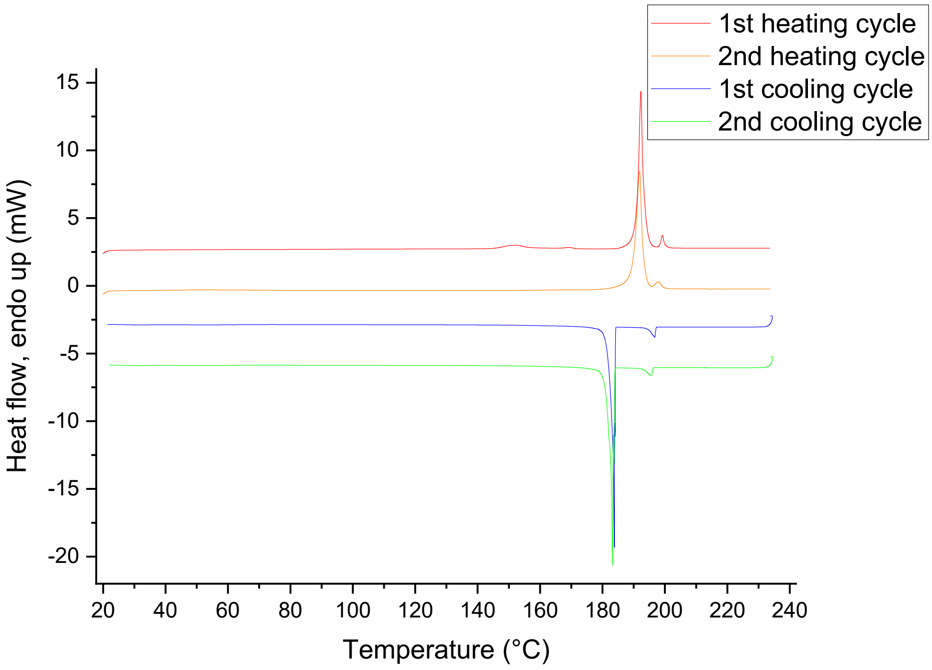

DSC curve for *t*-**Man*-SC10**

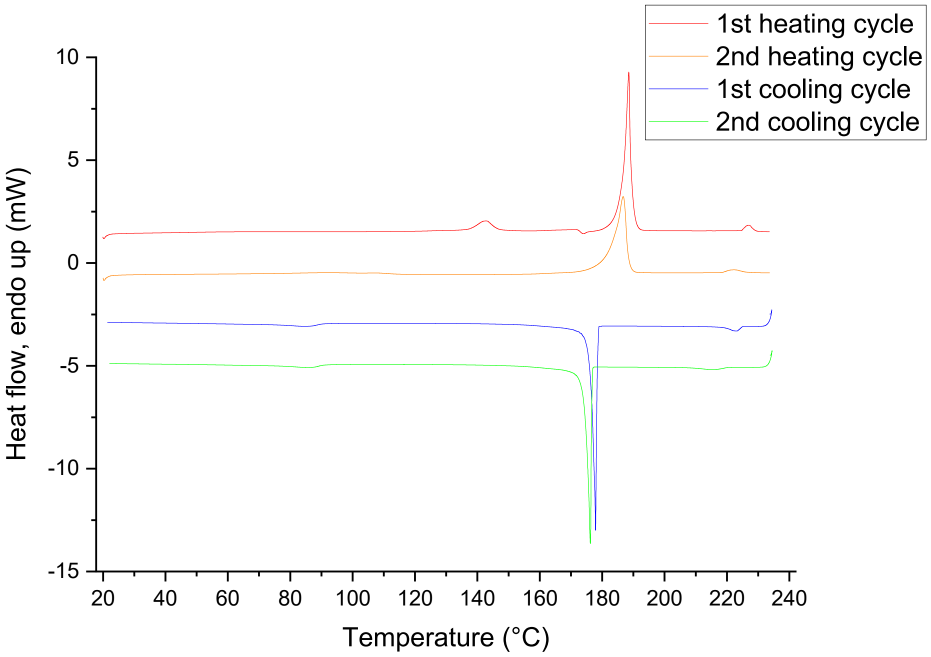

DSC curve for *t*-**Man*-SC14**

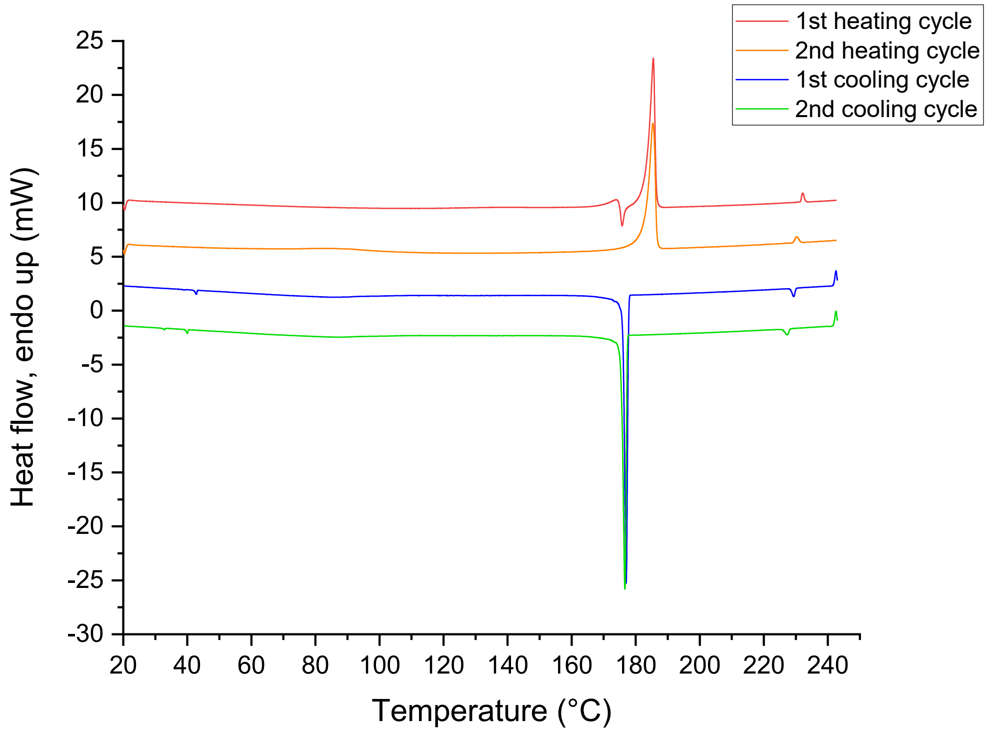

DSC curve for *t*-**Glc*-SC14**

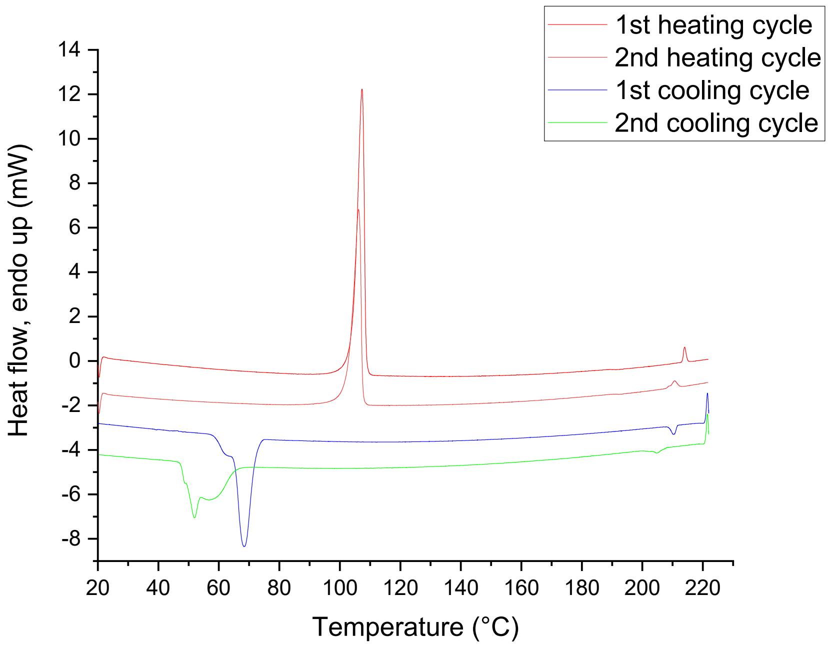

DSC curve for *e*-**Glc*-SC14**

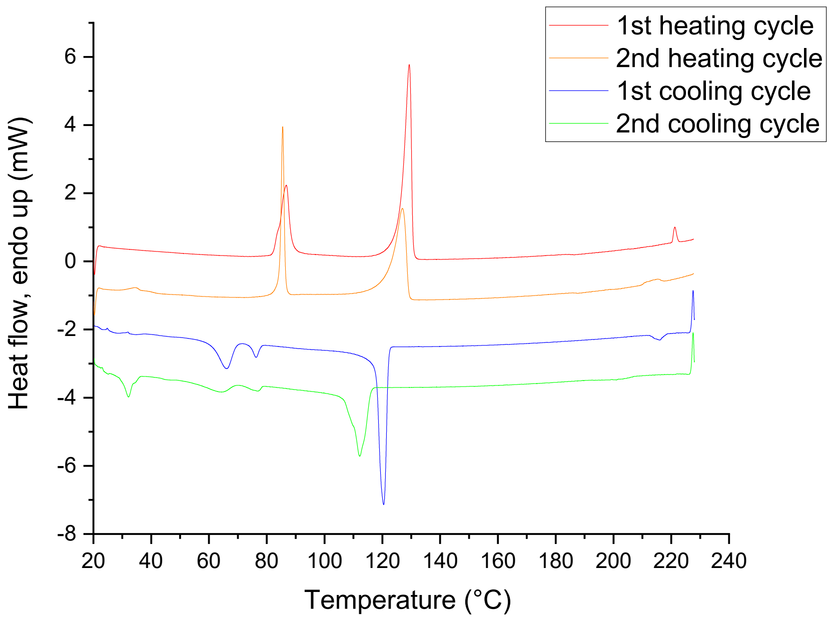

DSC curve for *t*-**Gal*-SC14**

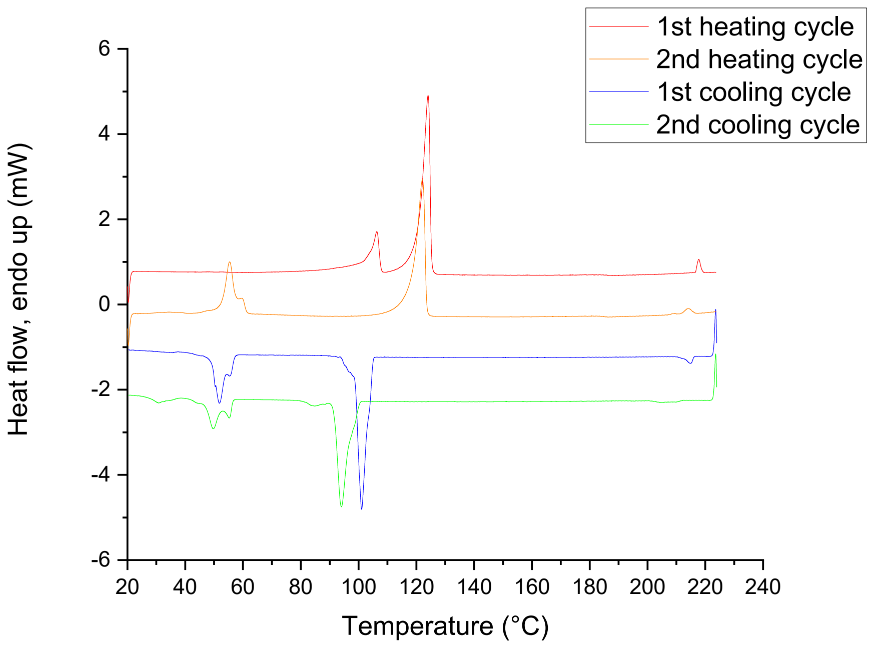

DSC curve for *e*-**Gal*-SC14**

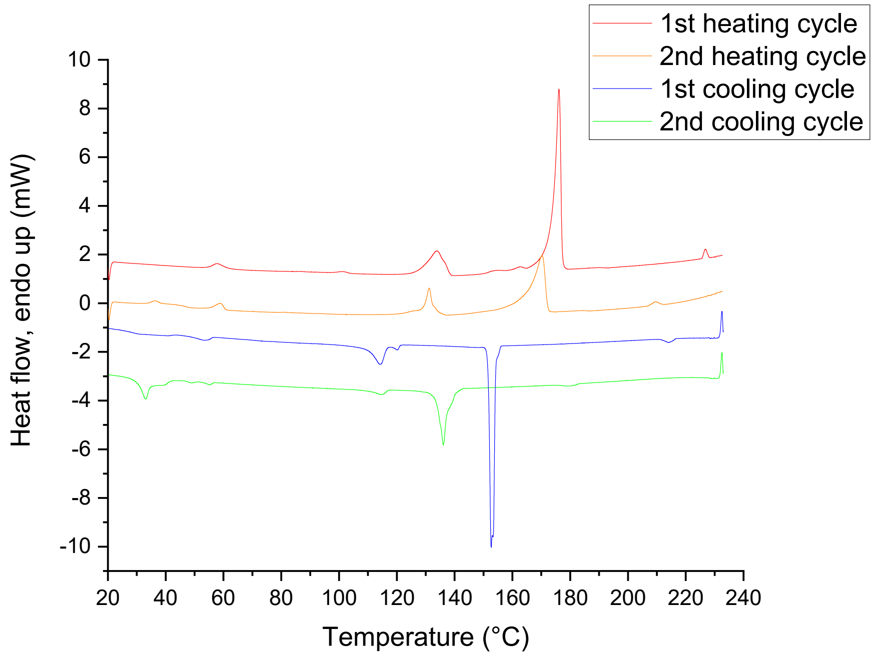

DSC curve for *t*-**Ara*-SC14**

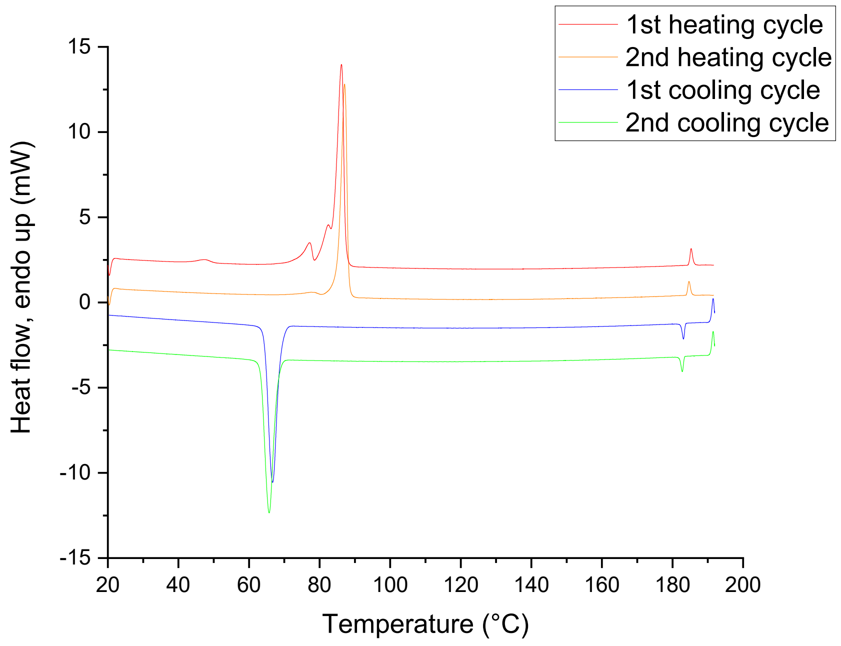

DSC curve for *e*-**Ara*-SC14**

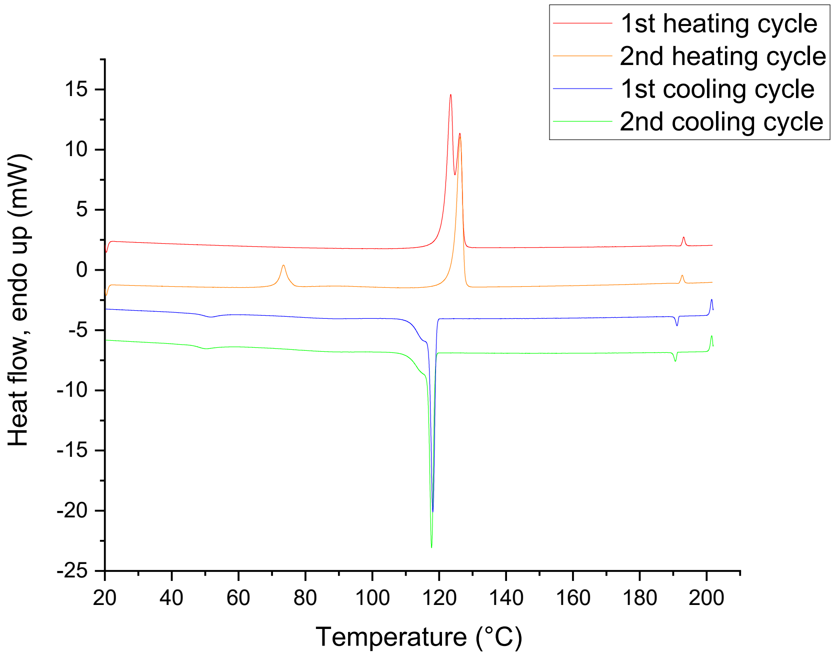

DSC curve for *t*-**Rha*-SC14**

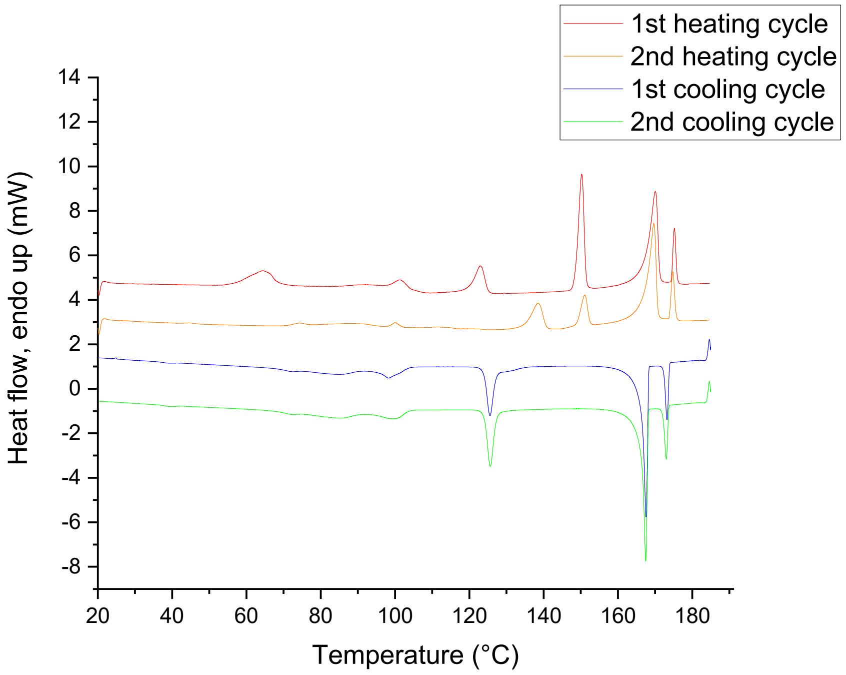

Polarized Optical Microscopy Observations

Mannose-based compound *t*-**Man*-SC6** showed a fairly narrow T_{LC} window (191-198 °C) and the smectic A phase was captured with POM by slight pressing at 198 °C during the 2nd cooling cycle. For *t*-**Man*-SC10**, the smectic A phase was captured at the 2nd heating cycle at 204 °C with slight pressing. Compound *t*-**Man*-SC10** melted slower than *t*-**Man*-SC14** into Sm liquid crystal phase. Compound *t*-**Man*-SC14** melted at around 183 °C without any visually observable solid-state transition. The liquid crystalline phase shows oily streaks and upon cooling the texture remains more or less the same in the solid phase.

Glucose-derived amphiphile *t*-**Glc*-SC14** is thermally the most "simple" of the compounds studied. The only thermal events observed are the crystalline melting and isotropization, with a liquid crystalline window spanning over 100 °C. The texture forms oily streaks. Compound *e*-**Glc*-SC14** exhibits a strong solid-solid transition at around 85 °C, which is observable only in the 2nd heating cycle with POM when the sample is a film. Compound *e*-**Glc*-SC14** melts slowly to the liquid crystalline phase, and the texture is similar to the other compounds studied. Upon cooling, the sample crystallizes clearly and the molten droplets shrink significantly upon crystallization.

The galactose derivative *t*-**Gal*-SC14** displays a solid-solid transition where colorful solid crystals appear at around 100 °C in the 1st heating cycle and at 50 °C in the 2nd heating cycle. The compound melts slowly to the liquid crystalline phase at 120 °C, forming an oily streak texture similar to the other compounds. The *erythro* galactose-derived diastereomer *e*-**Gal*-SC14** shows a solid-solid transition at around 130 °C where shiny crystals appear in POM. The compound melts to the liquid crystalline phase with the texture again identical to the other samples in the series. Upon cooling, however, the compound crystallizes sharply at around 150 °C. The borders of the phase are too sharp to be liquid, and this phase can be compressed and returns to its original shape once the pressure is released. The compound solidifies at 115 °C with a similar texture.

The arabinose-derivative *t*-**Ara*-SC14** melts sharply to liquid crystalline state and has a liquid crystal window of about 100 °C. The liquid crystal texture is similar to the other compounds and upon crystallization back to solid, a small change in the texture is observed. The *erythro* diastereomer *e*-**Ara*-SC14** likely consists of two polymorphs in bulk form. The first DSC heating cycle shows two overlapping peaks which are observable in POM by the disappearance of larger, shiny crystals, which either melt into liquid crystalline state or transform into another polymorph, whereafter the remaining sample melts into liquid crystalline state. The texture is again similar to the other amphiphilic compunds and upon cooling the sample crystallizes sharply to solid form. A solid-solid

transition in the form of a small shoulder in the crystallization peak in DSC can be observed by POM as a slight change in texture upon solidification.

The rhamnose derivative *t*-**Rha*-SC14** shows the most complex thermal behavior of the analyzed samples. Already in the solid state the sample looks different, forming larger crystals in the bulk form compared to the fine powders formed by the other amphiphilic sulfides. Some crystals are sufficiently large to shine under polarized light. These large, shiny crystals disappear in the two solid-solid transitions observed at around 60 and 100 °C in the first heating cycle, indicating the existence of more than one polymorph or other kinetic structures. At around 150 °C, compound *t*-**Rha*-SC14** forms a viscoelastic crystalline state. This state behaves like a highly viscous form of liquid crystal and can be compressed, but the boundaries are too sharp for a liquid. At about 170 °C, this state transforms into liquid crystal state with smectic texture similar to the rest of the samples. The liquid crystalline window is only a few degrees wide but still observable by POM before isotropization. Upon cooling, the sample again shows a narrow liquid crystalline state, whereafter it again adopts the viscoelastic state. The temperature range for this state is significantly wider during the cooling cycles, indicating supercooling of the sample. Upon solidification, the texture remains more or less unchanged.

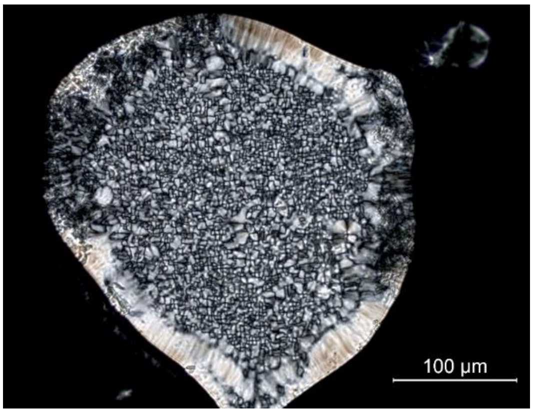

Figure S2. POM (crossed polars) image of the smectic phase (Sm) texture of compound *t*-**Man*-SC6** at 198 °C obtained after cooling and slightly pressing the sample cover glass.

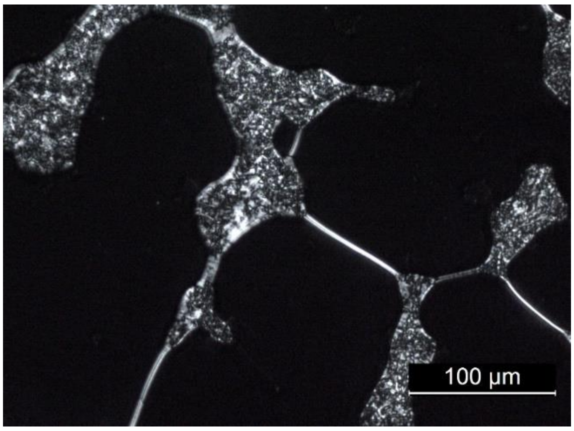

Figure S3. POM (crossed polars) image of the Sm phase of compound *t*-**Man*-SC10** at 204 °C obtained during 2nd heating after slightly pressing the sample cover glass.

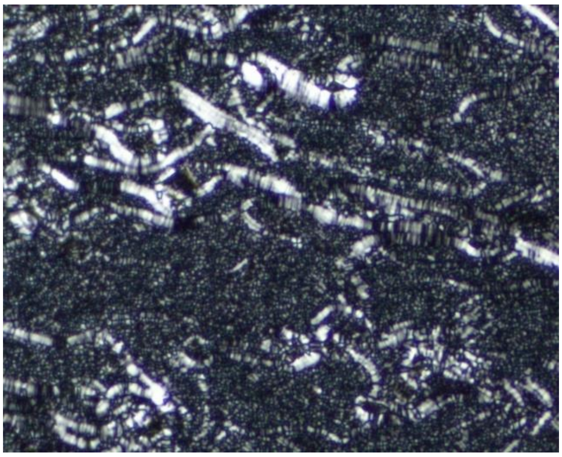

Figure S4. POM (crossed polars) image (zoomed-in from 100x magnification) of the Sm phase of compound *t*-**Man*-SC14** at 200 °C obtained during 2nd heating after slightly pressing the sample cover glass.

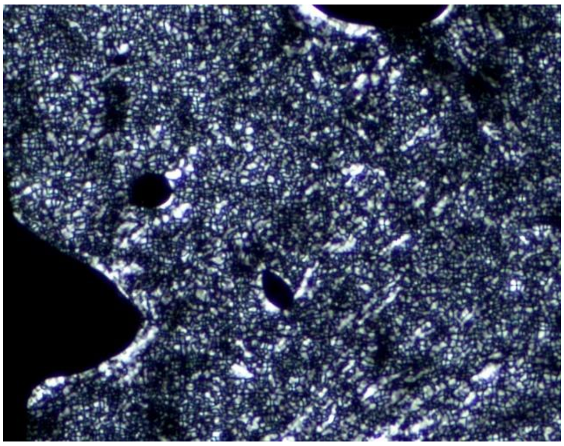

Figure S5. POM (crossed polars) image (zoomed-in from 100x magnification) of the Sm phase of compound *t*-**Glc*-SC14** at 110 °C obtained during 2nd heating after slightly pressing the sample cover glass.

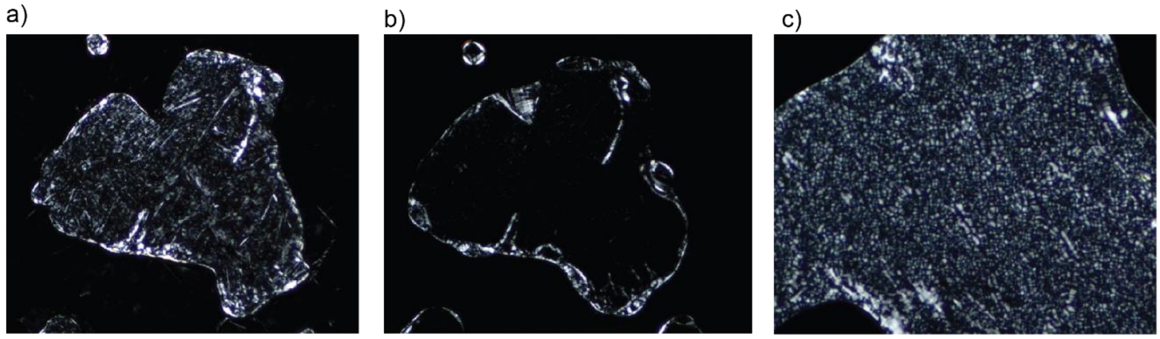

Figure S6. POM (crossed polars) images (zoomed-in from 100x magnification) of compound *e*-**Glc*-SC14**. a) Images taken during 2nd heating to 80 °C and continued to b**)** 130 °C where a decrease in birefringence has developed during heating. c) The Sm phase appears at 130 °C after pressing the sample glass cover.

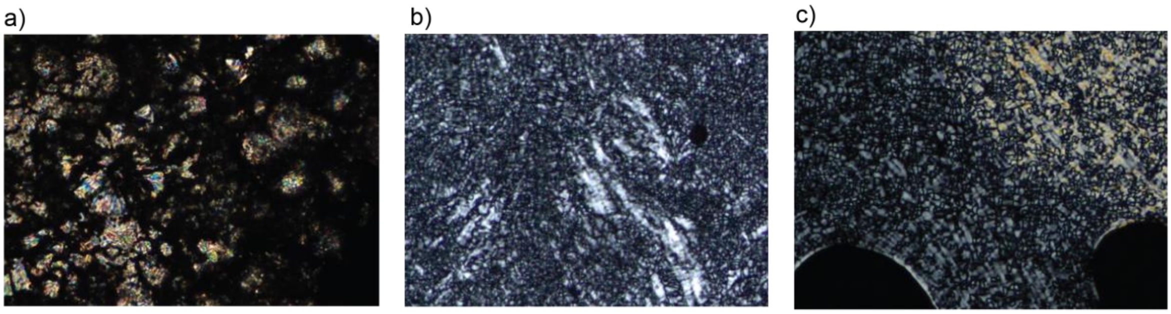

Figure S7. POM (crossed polars) images (zoomed-in from 100x magnification) of compound *t*-**Gal*-SC14**. a) At 114 °C during 1st heating iridescent color appear during melting. b) With continued heating the Sm phase appears at 125 °C after pressing. c) At 110 °C upon 1st cooling a Sm-solid transition appears with changes in birefringence (bright frontier expanses upon cooling).

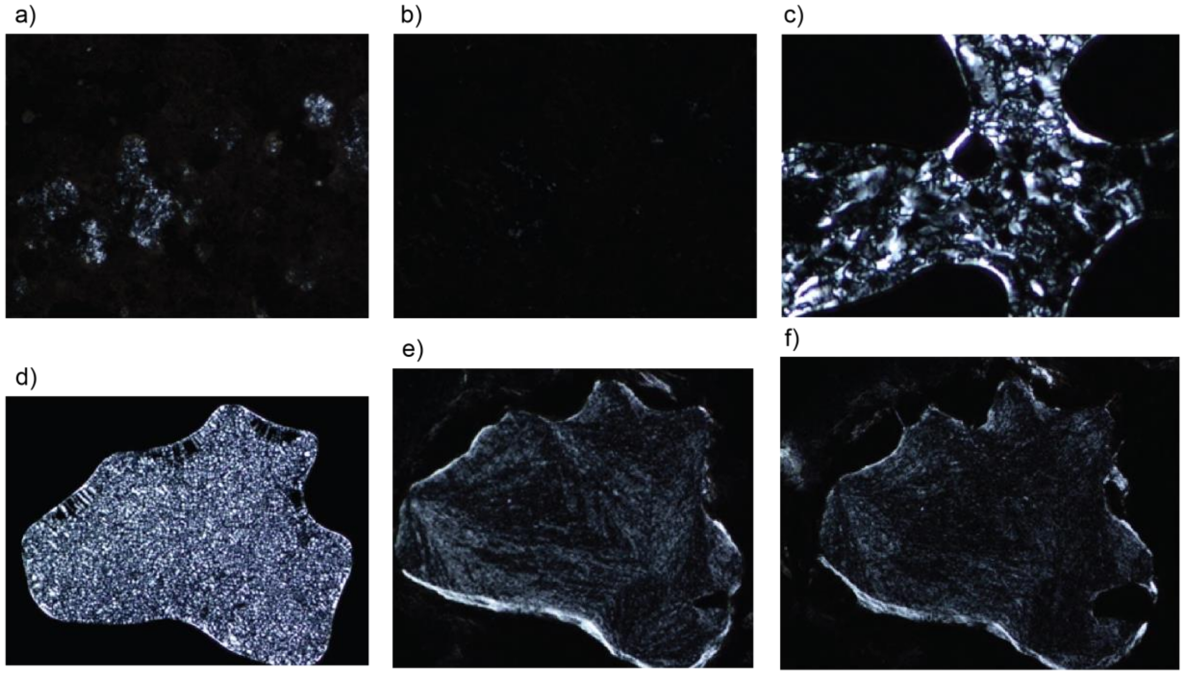

Figure S8. POM (crossed polars) images (zoomed-in from 100x magnification) of compound *e*-**Gal*-SC14.** During 1st heating at a) 146 °C the sample melts, yet at b) 165 °C it also loses birefringence before c) at 180 °C a Sm phase is obtained. During cooling the Sm phase appears at d) 180 °C and the isotropic viscose phase at e) 144 °C. The isotropic viscose phase transition is reproducible and during 2nd heating at f) 150 °C a loss of birefringence is observed and sample compresses with pressing on the glass slide.

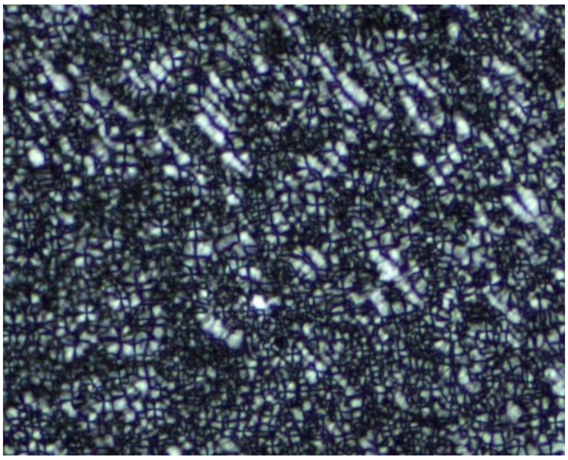

Figure S9. POM (crossed polars) image (zoomed-in from 100x magnification) of compound *t*-**Ara*-SC14** at 160 °C upon cooling showing the Sm phase texture.

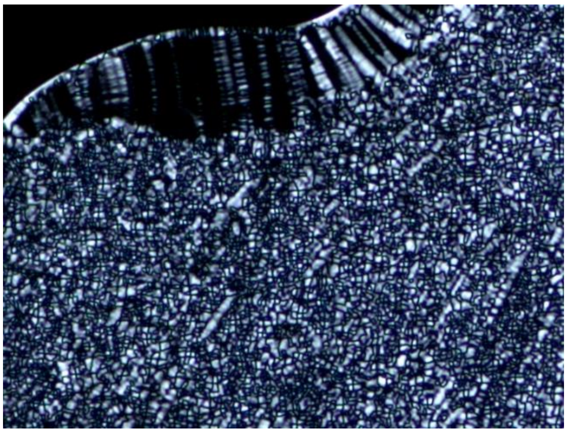

Figure S10. POM (crossed polars) image (zoomed-in from 100x magnification) of compound *e*-**Ara*-SC14** Sm phase at 177 °C upon cooling.

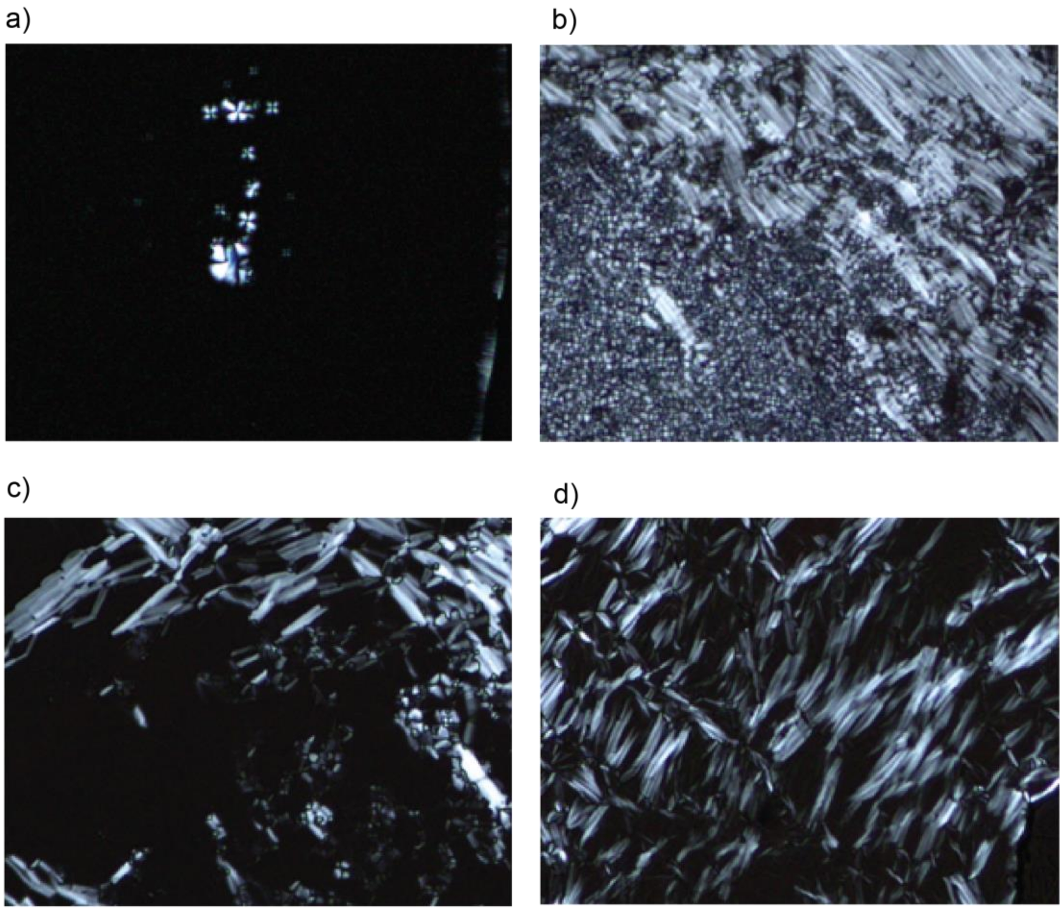

Figure S11. POM (crossed polars) images (zoomed-in from 100x magnification) of compound ***t*-Rha*-SC14** at a) 169 °C upon cooling and b) at 169 °C right after pressing the sample cover glass. c) At 115 °C upon cooling and pressing the sample, the mosaic texture disappears and is replaced by an isotropic phase. d) upon reheating to Sm phase and cooling back to 146 °C, a unique liquid crystalline texture is obtained.

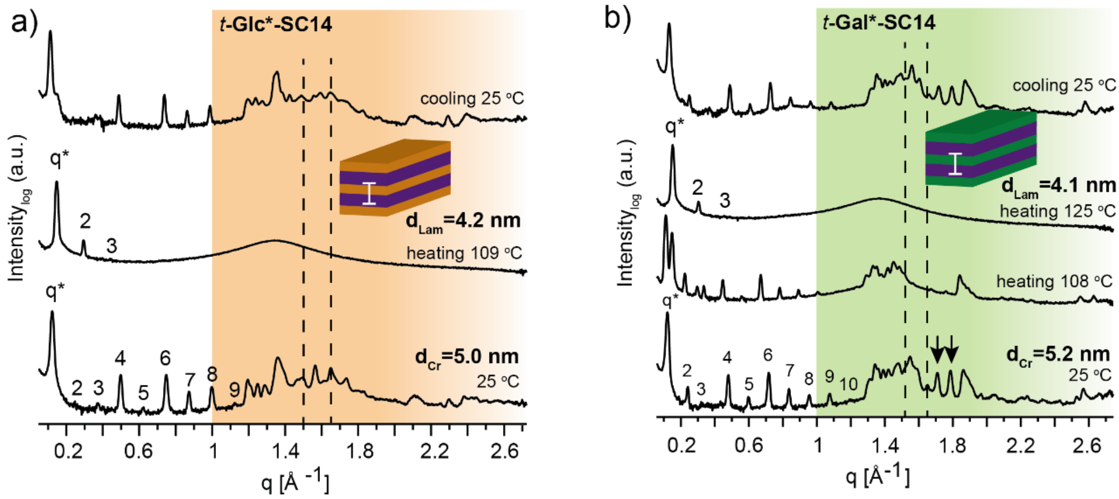

Figure S12. Selected SWAXS profiles for a) ***t*-Glc*-SC14** and b) ***t*-Gal*-SC14** compounds. Dashed lines indicate the position of the crystalline alkyl tail peaks. Colored areas represent the wide-angle regions. Black arrows indicate WAXS peaks for polyol block structure at 25 °C, which intensity decreases upon heating to 108 °C demonstrating the hydrogen bonding rearrangement within the polyol blocks.

Table S4. ***e*-Glc*-SC14** (at 109 °C) cubic phase structure parameter estimation using the Pm3m (simple cubic structure) structure lattice parameters with program Scatter SWAXS data analyzing program. Cubic lattice parameter of $\mathbf{a}_{Cub}$=6.1 nm was calculated with Scatter.

hkl	q/q*	q_{hkl} [nm] Scatter fit
100	1	0.103
110	√2	0.146
111	√3	0.180
200	2	0.207
211	√6	0.255
220	√8	0.292
221	3	0.310
400	4	0.413
500	5	0.517
600	6	0.622
700	7	0.723
800	8	0.820
900	9	0.922

Table S5. ***e*-Gal*-SC14** (at 135 °C) cubic phase structure parameter estimation using the Pm3m (simple cubic structure) structure lattice parameters with program Scatter SWAXS data analyzing program. Cubic lattice parameter of $\mathbf{a}_{Cub}$=5.6 nm was calculated with Scatter.

hkl	q/q*	q_{hkl} [nm] Scatter fit
100	1	0.111
110	√2	0.158
111	√3	0.194
200	2	0.224
222	√12	0.388
311	√11	0.371
400	4	0.448
431	√27	0.571
600	6	0.671
620	√40	0.708
621	√41	0.717
533	√43	0.734
622	√44	0.742
700	7	0.783

Table S6. ***e*-Ara*-SC14** (at 122 °C) cubic phase structure parameter estimation using the Pm3m (simple cubic structure) structure lattice parameters with program Scatter SWAXS data analyzing program. Cubic lattice parameter of $\mathbf{a}_{Cub}$=5.8 nm was calculated with Scatter.

hkl	q/q*	q_{hkl} [nm] Scatter fit
100	1	0.108
110	√2	0.153
111	√3	0.187
200	2	0.216
211	√6	0.265
220	√8	0.306
300	3	0.325
222	√15	0.375
400	4	0.433
411	√18	0.460
600	6	0.650
621	√41	0.693
700	7	0.757
710	√50	0.765
800	8	0.866

Table S7. *t*-**Rha*-SC14** (at 125 °C) cubic phase structure parameter estimation using the Pm3m (simple cubic structure) structure lattice parameters with program Scatter SWAXS data analyzing program. Cubic lattice parameter of $\mathbf{a}_{Cub}$=4.9 nm was calculated with Scatter

hkl	q/q*	q_{hkl} [nm] Scatter fit
100	1	0.127
110	√2	0.180
111	√3	0.221
200	2	0.255
300	3	0.382
400	4	0.509
422	√24	0.624
500	5	0.637
600	6	0.764
700	7	0.891

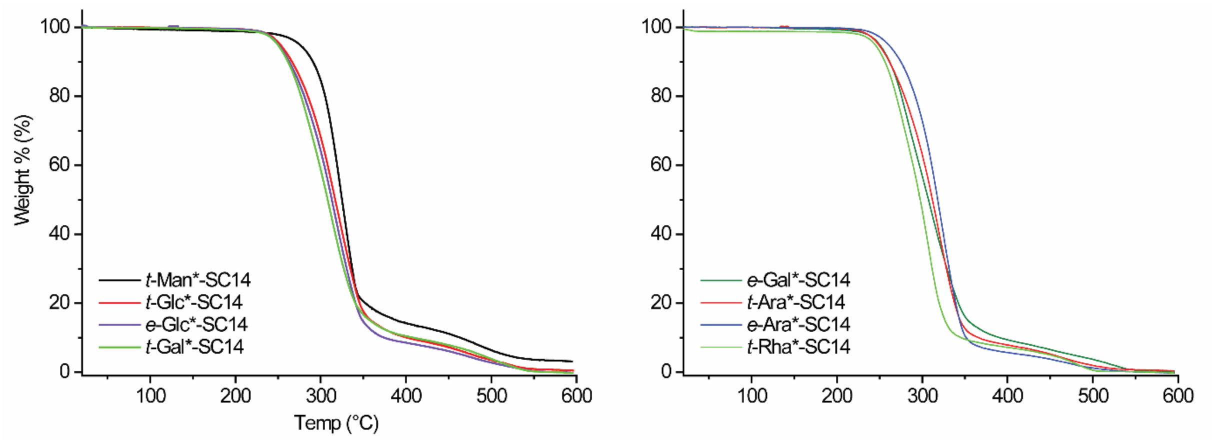

Figure S13. TGA graphs for all compounds studied with SWAXS.

References

1 W. Schmid and G. M. Whitesides, *J. Am. Chem. Soc.*, 1991, **113**, 6674–6675.

2 E. Kim, D. M. Gordon, W. Schmid and G. M. Whitesides, *J. Org. Chem.*, 1993, **58**, 5500–5507.

3 T. Saloranta, A. Peuronen, J. M. Dieterich, J. Ruokolainen, M. Lahtinen and R. Leino, *Cryst. Growth Des.*, 2016, **16**, 655–661.

4 T. Saloranta, C. Müller, D. Vogt and R. Leino, *Chem. - A Eur. J.*, 2008, **14**, 10539–10542.

5 B. Delley, *J. Chem. Phys.*, 1990, **92**, 508–517.

6 B. Delley, *J. Chem. Phys.*, 2000, **113**, 7756–7764.

7 A. D. Bochevarov, E. Harder, T. F. Hughes, J. R. Greenwood, D. A. Braden, D. M. Philipp, D. Rinaldo, M. D. Halls, J. Zhang and R. A. Friesner, *Int. J. Quantum Chem.*, 2013, **113**, 2110–2142.

8 Maestro and Schrödinger, 2019.

9 A. D. Becke, *J. Chem. Phys.*, 1993, **98**, 5648–5652.

10 C. Lee, W. Yang and R. G. Parr, *Phys. Rev. B*, 1988, **37**, 785–789.

11 S. H. Vosko, L. Wilk and M. Nusair, *Can. J. Phys.*, 1980, **58**, 1200–1211.

12 P. J. Stephens, F. J. Devlin, C. F. Chabalowski and M. J. Frisch, *J. Phys. Chem.*, 1994, **98**, 11623–11627.

13 E. R. McNellis, J. Meyer and K. Reuter, *Phys. Rev. B*, 2009, **80**, 205414.

14 B. Delley, *Mol. Simul.*, 2006, **32**, 117–123.
